# Palmitoyltransferase ZDHHC3 Aggravates Nonalcoholic Steatohepatitis by Targeting *S*‐Palmitoylated IRHOM2

**DOI:** 10.1002/advs.202302130

**Published:** 2023-08-06

**Authors:** Minxuan Xu, Jun Tan, Liancai Zhu, Chenxu Ge, Yi Zhang, Fufeng Gao, Xianling Dai, Qin Kuang, Jie Chai, Benkui Zou, Bochu Wang

**Affiliations:** ^1^ Chongqing Key Laboratory of Medicinal Resources in the Three Gorges Reservoir Region School of Biological and Chemical Engineering Chongqing University of Education Chongqing 400067 P. R. China; ^2^ College of Modern Health Industry Chongqing University of Education Chongqing 400067 P. R. China; ^3^ Key Laboratory of Biorheological Science and Technology (Chongqing University) Ministry of Education College of Bioengineering Chongqing University Chongqing 400030 P. R. China; ^4^ Department of Gastrointestinal Surgery Shandong Cancer Hospital and Institute Shandong First Medical University&Shandong Academy of Medical Science Jinan 250117 P. R. China

**Keywords:** hepatosteatosis, inactive rhomboid protein 2 (IRHOM2), nonalcoholic steatohepatitis (NASH), Palmitoylation, zinc finger DHHC‐type palmitoyltransferase 3 (ZDHHC3), tripartite motif containing 31 (TRIM31)

## Abstract

Underestimation of the complexity of pathogenesis in nonalcoholic steatohepatitis (NASH) significantly encumbers development of new drugs and targeted therapy strategies. Inactive rhomboid protein 2 (IRHOM2) has a multifunctional role in regulating inflammation, cell survival, and immunoreaction. Although cytokines and chemokines promote IRHOM2 trafficking or cooperate with partner factors by phosphorylation or ubiquitin ligases‐mediated ubiquitination to perform physiological process, it remains unknown whether other regulators induce IRHOM2 activation via different mechanisms in NASH progression. Here the authors find that IRHOM2 is post‐translationally *S*‐palmitoylated at C476 in iRhom homology domain (IRHD), which facilitates its cytomembrane translocation and stabilization. Fatty‐acids challenge can directly promote IRHOM2 trafficking by increasing its palmitoylation. Additionally, the authors identify Zinc finger DHHC‐type palmitoyltransferase 3 (ZDHHC3) as a key acetyltransferase required for the IRHOM2 palmitoylation. Fatty‐acids administration enhances IRHOM2 palmitoylation by increasing the direct association between ZDHHC3 and IRHOM2, which is catalyzed by the DHHC (C157) domain of ZDHHC3. Meanwhile, a metabolic stresses‐triggered increase of ZDHHC3 maintains palmitoylated IRHOM2 accumulation by blocking its ubiquitination, consequently suppressing its ubiquitin‐proteasome‐related degradation mediated by tripartite motif containing 31 (TRIM31). High‐levels of ZDHHC3 protein abundance positively correlate with the severity of NASH phenotype in patient samples. Hepatocyte‐specific dysfunction of ZDHHC3 significantly inhibits palmitoylated IRHOM2 deposition, therefore suppressing the fatty‐acids‐mediated hepatosteatosis and inflammation in vitro, as well as NASH pathological phenotype induced by two different high‐energy diets (HFHC & WTDF) in the in vivo rodent and rabbit model. Inversely, specific restoration of ZDHHC3 in hepatocytes markedly provides acceleration over the course of NASH development via increasing palmitoylation of IRHOM2 along with suppression of ubiquitin degradation. The current work uncovers that ZDHHC3‐induced palmitoylation is a novel regulatory mechanism and signal that regulates IRHOM2 trafficking, which confers evidence associating the regulation of palmitoylation with NASH progression.

## Graphical abstract

Elevated palmitoylation of IRHOM2 by ZDHHC3 facilitates its membrane localization and accumulation. Excessive IRHOM2 abundance significantly recruits its downstream signaling hyperactivation, thereby accelerating steatohepatitis progression in response to high‐energy diet challenge. In the meantime, palmitoylation‐stabilized IRHOM2 positively blocks its ubiquitination and TRIM31‐mediated proteasome degradation via decreasing ubiquitin of K48 linkage.

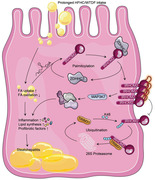

## 1 Introduction

A significant increase in the incidence of nonalcoholic fatty liver disease (NAFLD), nonalcoholic steatohepatitis (NASH), and its associated complications has been regarded as a global epidemic.^[^ ^1^, ^2^ ^]^ With the continuous increase of the global fatty liver population, complications related to steatohepatitis, e.g., hyperlipidemia, hyperglycemia, fibrosis, atherosclerosis, type 2 diabetes (T2D), and stroke have markedly elevated in parallel.^[^ ^2^, ^3^ ^]^ In particular, NASH is fast becoming the major liver disease in the developed world and is recognized as a major driver of hepatocellular carcinoma (HCC).^[^ ^4^ ^]^ The severity of fatty liver pathological process ranges from simple hepatosteatosis of the liver to worsening hepatocellular injury, and necroinflammatory pathology as determined by NASH, indicating that patients are at higher risk of liver fibrosis and HCC, and are more likely to these pathological progressions.^[^ ^4^, ^5^ ^]^ However, the mechanisms of NASH pathogenesis are not fully understood. As of now, it has no approved effective treatment drugs or therapeutic strategies for NASH, and efforts to study the complications associated with NASH have not fully met expectations. Furthermore, a series of potential preclinical drugs for NASH treatment, e.g., obeticholic acid (OCA), emricasan (VAY785), pegbelfermin (BMS‐986036), and selonsertib (GS‐4997), have failed in clinical trials because of unachieved clinical endpoints.^[^ ^6^, ^7^, ^8^, ^9^ ^]^ Recently, resmetirom (MGL‐3196), a liver‐directed, orally active, selective THR‐β agonist designed to improve NASH has reached two main endpoints for the first time in the MEASTRO‐NASH three‐stage clinical trial, showing strong effectiveness, business value, and future expectations.^[^ ^10^ ^]^ But what is undeniable is that the failure of these promising preclinical drugs provides further evidence that the pathogenesis progression of NASH involves complicated molecular mechanisms and may be tracked with many physiological and metabolic processes. Therefore, further in‐depth study is needed to accurately and comprehensively identify the key molecular regulators of NASH development, so as to develop more effective therapeutic regimens.

Inactive rhomboid protein 2 (IRHOM2, also known as RHBDF2) has been determined as a multifunctional regulator in the innate immune response and inflammation‐related diseases.^[^ ^11^, ^12^ ^]^ Its abundance in cells is under the regulation of a series of factors that are still under in‐depth study. Increased cellular IRHOM2 accumulation is capable of cooperating with its known partner proteins such as STING, FRMD8, and 14‐3‐3 to mediate trafficking of targeted factors or themselves, thereby regulating cell physiological activity.^[^ ^13^, ^14^, ^15^ ^]^ Recent studies have indicated the significance of posttranslational modifications in regulating the expression of IRHOM2. Localization of IRHOM2 in the lysosomes by deletion of FRMD8 (iRhom tail‐associated protein) facilitates lysosomal degradation of IRHOM2, while the presence of FRMD8 recruitment to the IRHOM2 retards the above process.^[^ ^14^, ^16^ ^]^ Also, IRHOM2 can act as a bridging factor to interact with STING, and sustain STING stabilization by suppressing its ubiquitination degradation, therefore causing a reaction to viral infections.^[^ ^13^ ^]^ Furthermore, our recent works have revealed that in response to metabolic stress challenges, IRHOM2 was found to largely accumulate in the proteasome, indicating that ubiquitin‐proteasome degradation is another important mechanism for IRHOM2 protein fate regulation.^[^ ^17^, ^18^, ^19^ ^]^ Increased ubiquitination of IRHOM2 by E3 ubiquitin ligases tripartite motif containing 31 (TRIM31) promotes its degradation via ubiquitination of K48‐linkage, while proteasome inhibitors block the above process.^[^ ^17^ ^]^ However, ubiquitin‐specific peptidase 13 (USP13) did destabilize IRHOM2 expression by removing K63‐linked ubiquitination of IRHOM2, and a specific activator treatment targeting USP13 displayed potential treatment effects.^[^ ^18^ ^]^ Interestingly, during prolonged metabolic stress administration, e.g., palmitate & oleic acid, an increase in IRHOM2 abundance in the cytoplasm significantly suppressed ubiquitination degradation triggered by TRIM31, followed by an acceleration of IRHOM2 trafficking and membrane deposition. These findings also further illustrate that under different physiological and pathological conditions, IRHOM2 may have a variety of different posttranslational modifications synergistically or antagonistically determine its protein fate.

Among the many forms of protein chemical modifications, palmitoylation plays a decisive role in the regulation of a series of functional proteins.^[^ ^20^ ^]^ Posttranslational *S*‐palmitoylation binds C_16_ fatty acid palmitate to the cysteine residues of a protein, and regulates diverse functions of proteins under physiological and pathological conditions.^[^ ^20^, ^21^ ^]^ Of note, palmitoylation has previously been shown to regulate the membrane localization and trafficking of targeted proteins.^[^ ^21^, ^22^ ^]^ This biological process is dependent on catalysis by an acyltransferase family known as Zinc finger‐DHHC (Aspartate‐histidine‐histidine‐cysteine)‐CRD (Cysteine rich domain)‐type palmitoyl acyltransferases,^[^ ^23^, ^24^ ^]^ however, it is largely unknown whether palmitoylation participates in post‐translation modification of IRHOM2 and the effects on IRHOM2 function.

In our current work, we reveal that IRHOM2 is posttranslationally *S*‐palmitoylated at the C476 residue by the ZDHHC3 palmitoyltransferase, which promotes its cytomembrane translocation and stabilization. Metabolic insults accelerate ZDHHC3‐mediated palmitoylated IRHOM2 abundance, and this modification blocks IRHOM2 ubiquitin‐proteasome‐related degradation induced by TRIM31 via the removal of ubiquitination chains. Disturbance of the palmitoylation of IRHOM2 by ZDHHC3 deletion in hepatocytes or 2‐bromopalmitate (2‐BP), a general inhibitor of protein *S*‐palmitoylation, mitigates the symptoms in rodent and rabbit models with the NASH phenotype, thus also indicating a potential promising therapeutic strategy for the treatment of NASH.

## 2 Results

### 2.1 IRHOM2 is Palmitoylated, and IRHOM2 Expression is Stabilized by *S*‐Palmitoylation

**Figure 1:**
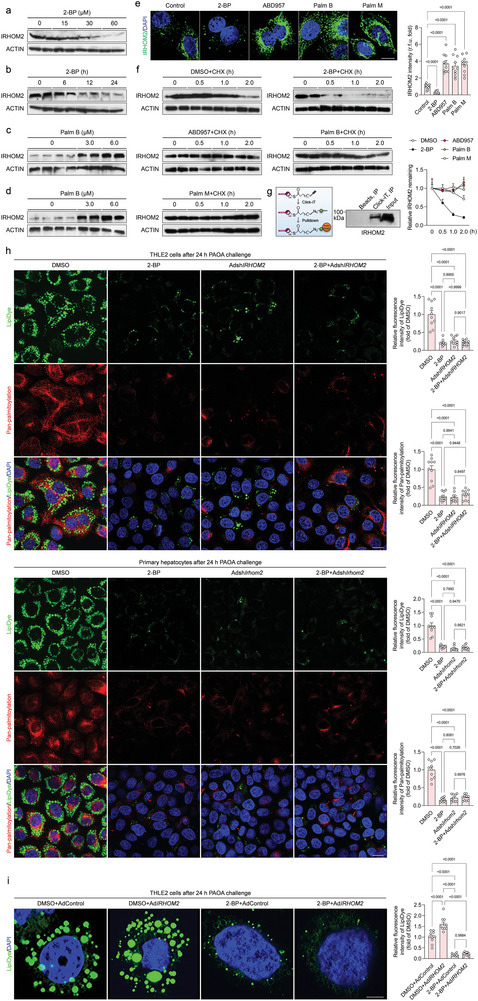
Identification of palmitoylated‐modificated IRHOM2 in hepatocytes. a) Human hepatocyte THLE2 cells were incubated with a gradually increasing dose of 2‐bromopalmitate (2‐BP) for 24 h, and subjected to immunoblotting detection with IRHOM2 and ACTIN antibodies (*n* = 4 per group). b) THLE2 cells were treated with 60 µM 2‐BP for 0, 6, 12, and 24 h and then subjected to immunoblotting assay with IRHOM2 and ACTIN antibodies (*n* = 4 per group). c, d) THLE2 cells (c) and primary human hepatocyte, PHH (d) were incubated with 0, 3, or 6 µM palmostatin B (Palm B), an inhibitor of depalmitoylase enzymes, for 24 h, and subjected to immunoblotting analysis with IRHOM2 and ACTIN antibodies (*n* = 4 per group). e) THLE2 cells were treated with 60 µM 2‐BP, 1 µM ABD957, 6 µM Palm B, and 10 µM palmostatin M (Palm M) for 24 h, respectively. Left, the fixed sections were subjected to immunofluorescent staining with IRHOM2 (green) and DAPI (blue). Right, the bar graph shows IRHOM2 fluorescence intensity in the indicated group (*n* = 10 images per group; *P* < 0.05 versus Control). Scale bars, 10 µm. f) THLE2 cells were preincubated with DMSO, 2‐BP, ABD957, Palm B, or Palm M as a baseline, then treated with cycloheximide (CHX) in the time‐course frame, respectively. The collected cell lysates were subjected to immunoblotting detection with IRHOM2 and ACTIN antibodies. The right curve graph shows the relative IRHOM2 remaining ratio in the indicated time point (*n* = 4 per group). g) Sketch map of the Click‐iT assay used for IRHOM2 palmitoylation analysis. THLE2 cells were incubated with 120 µM Click‐iT palmitic acid‐Azides for 8 h, and corresponding collected lysates were subjected to Click‐iT detection in accordance with product instruction, followed by western blotting analysis with IRHOM2 antibody. The right western blotting bands show the IRHOM2 expression in the indicated group. h, Representative images of immunofluorescent staining showing the lipid droplets formation and deposition (LipiDye, green) and universal palmitoylation levels (red) in THLE2 cells (upper) and mouse primary hepatocytes (lower) after transfection with indicated vectors in response to PAOA treatment for 24 h (*n* = 10 images per group; *P* < 0.05 versus DMSO). Scale bars, 5 µm. i) Representative images of immunofluorescent staining showing the lipid droplets formation and accumulation (LipiDye, green) in Ad*IRHOM2*‐ or 2‐BP‐treated THLE2 cells after 24 h PAOA challenge (*n* = 10 images per group; *P* < 0.05 versus DMSO). Scale bars, 10 µm. Data are expressed as mean ± SEM. The relevant experiments presented in this part were performed independently at least three times. Significance is determined by one‐way analysis of variance (ANOVA) followed by Dunnett's multiple comparisons test analysis (e, h, i). The *P*‐value < 0.05 was considered as significant difference.

To investigate whether palmitoylated IRHOM2 was involved in fatty liver diseases, we first examined its expression profile alterations upon 2‐bromopalmitate (2‐BP), a general inhibitor of protein palmitoylation challenge.^[^ ^25^ ^]^ When human hepatocyte THLE2 cells were incubated with a concentration gradient of 2‐BP to downregulate palmitoylated proteins (Supplementary Figure S1a), the IRHOM2 protein expression was markedly reduced in both dose‐dependent (**Figure** **1**a) and time‐dependent manners (Figure 1b). Similar decreased effects were also observed in mouse primary hepatocytes over the course of a specified time or concentration of 2‐BP treatment (Figure S1b, Supporting Information). Conversely, significant upregulation in palmitoylation by palmostatin B (Palm B) or ABD957, the inhibitor of depalmitoylase enzymes elevated IRHOM2 protein levels in THLE2 cells and primary human hepatocyte (PHH) (Figure 1c,d), and mouse primary hepatocytes (Figure S1b, Supporting Information). Such effects of 2‐BP, ABD957, Palm B, and Palm M on IRHOM2 expression were further determined by immunofluorescence analysis (Figure 1e). Also, cultured cells with cycloheximide (CHX) treatment confirmed that blockade of palmitoylation by 2‐BP challenge promoted IRHOM2 degradation, therefore reducing its protein abundance amplitude, while ABD957, Palm B, and Palm M sufficiently maintained IRHOM2 abundance (Figure 1f and Figure S1c, Supporting Information). Considering the effects of 2‐BP on the reduction of IRHOM2 expression, a click chemistry assay was employed to visualize the palmitoylated IRHOM2 (Figure 1g). As expected, conspicuous palmitoylation of IRHOM2 was confirmed by the streptavidin beads‐mediated pull‐down and western blotting analysis (Figure 1g). In addition, to preferentially study the effect of palmitoylation on lipid metabolism, human and mouse hepatocytes were cotreated with 0.5 mM palmitic acid+1.0 mM oleic acid (PAOA) mixture or serum from NASH patients, accompanied by different inhibitors, respectively. Unsurprisingly, lipid accumulation was significantly lower during 2‐BP treatment of both THLE2 cells (Figure S1d, Supporting Information) and mouse primary hepatocytes (Figure S1e, Supporting Information) that were incubated with PAOA and NASH serum for 24 h than in the corresponding controls. Oppositely, inhibitor of depalmitoylase enzymes (ABD957, Palm B, and Palm M) intervention is capable of facilitating lipid deposition in hepatocytes and intracellular triglyceride (TG) contents.

Furthermore, given that we have determined the IRHOM2 *S*‐palmitoylation occurred and exhibited a positive correlation with dysregulated lipid metabolism during NASH, to additionally confirm PAOA‐induced increase of IRHOM2 *S*‐palmitoylation contributed to abnormal lipid metabolism, adenovirus‐mediated IRHOM2 knockdown (Adsh*IRHOM2*) transfected‐, 2‐BP treated‐ or 2‐BP+Adsh*IRHOM2* cotreated‐THLE2 cells were used respectively to suppress palmitoylation, IRHOM2 expression levels, and palmitoylated IRHOM2 expression, followed by detection of intracellular lipid deposition. Inevitably, as shown in Figure 1h, using adenovirus‐mediated suppression of IRHOM2 expression to simultaneously decrease IRHOM2 protein expression and its palmitoylation modification, consistent with our above findings, both 2‐BP, Adsh*IRHOM2*, and 2‐BP+Adsh*IRHOM2* cotreatment intervention did significantly reduce lipid deposition in human THLE2 cells and mouse primary hepatocytes during PAOA challenge. Compared to the DMSO group, the lipid levels in 2‐BP+Adsh*IRHOM2* cotreatment resemble the intracellular lipid contents observed in cells with only 2‐BP or Adsh*IRHOM2* intervention, suggesting that 2‐BP‐mediated blockage of IRHOM2 palmitoylation did contribute to the interruption of lipid metabolism dysfunction in response to lipotoxicity challenge. Besides, to deeply determine the palmitoylated IRHOM2 directly promoted dysregulated lipid metabolism, the adenovirus‐mediated IRHOM2 overexpression and 2‐BP cotreatment were also used to analyze the alteration of lipid contents in the presence of PAOA (Figure 1i). In agreement with our other data, palmitoylated IRHOM2 is required for its biological function in acceleration of dysregulated lipid metabolism. Undoubtedly, 2‐BP intervention considerably blocked IRHOM2 overexpression‐mediated increase in lipid accumulation in vitro, as compared to DMSO+AdControl group and DMSO+Ad*IRHOM2* group, indicating that IRHOM2 palmitoylation did conduce to the dysregulated lipid metabolism, and that 2‐BP‐triggered restrained palmitoylation of IRHOM2 significantly protected against disturbance of lipid metabolism. The above results indicated that IRHOM2 expression is stabilized by *S*‐palmitoylation, and increased palmitoylation contributed to dysregulated lipid metabolism in vitro.

### 2.2 Identification of the Palmitoylation Site in IRHOM2

**Figure 2:**
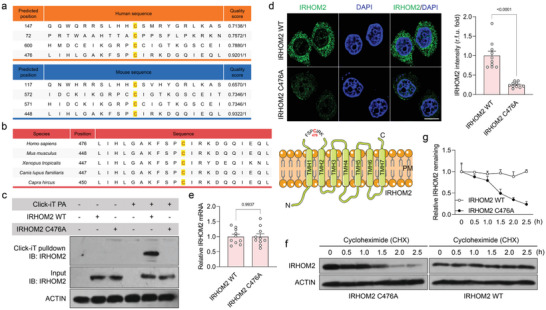
Identification of the palmitoylation site on IRHOM2 at evolutionarily conserved cysteine residues. a) Predicted position of palmitoylation site on IRHOM2 in *Homo sapiens* (upper) and *Mus musculus* (lower) using GPS‐Palm software (MacOS_20 200 219) (The CUCKOO Workgroup, http://gpspalm.biocuckoo.cn/), and MDD‐Palm algorithm (http://csb.cse.yzu.edu.tw/MDDPalm/). b) Left, palmitoylation site of IRHOM2 with conserved cysteine residues in *Homo sapiens*, *Mus musculus*, *Xenopus tropicalis*, *Canis lupus familiaris*, and *Capra hircus*. Right, schematic diagram showing the IRHOM2 wild‐type (WT) structure of *Homo sapiens* and possible palmitoylation site. c) THLE2 cells with IRHOM2 WT or IRHOM2 C476A mutant overexpression were incubated with Click‐iT palmitic acid‐Azides for 8 h, and corresponding collected lysates were subjected to Click‐iT detection in accordance with product instruction. The palmitoylated proteins were placed onto the pull‐down detection by streptavidin‐sepharose bead conjugate, followed by western blotting analysis with IRHOM2 and ACTIN antibodies. The palmitoylation of IRHOM2 WT was observed in top gel, lane 5, but not for the IRHOM2 C476A in top gel, lane 6, or control groups. This experiment was repeated 3 times independently. d) THLE2 cells were overexpressed with IRHOM2 WT or IRHOM2 C476A mutant, respectively. Left, the fixed sections were subjected to immunofluorescent staining with IRHOM2 (green) and DAPI (blue). Right, the bar graph shows IRHOM2 fluorescence intensity in the indicated group (*n* = 10 images per group; *P* < 0.05 versus IRHOM2 WT). Scale bars, 10 µm. e) qPCR analysis showing the IRHOM2 mRNA levels in IRHOM2 WT or IRHOM2 C476A mutant (*n* = 10 per group; *P* < 0.05 versus IRHOM2 WT). f, g) THLE2 cells with IRHOM2 WT or IRHOM2 C476A mutant overexpression were incubated with CHX in a time‐course frame, respectively. The collected cell lysates were subjected to immunoblotting detection with IRHOM2 and ACTIN antibodies (f). The curve graph (g) shows the relative IRHOM2 remaining ratio in the indicated time point (*n* = 4 per group). Data are expressed as mean ± SEM. The relevant experiments presented in this part were performed independently at least three times. Significance determined by 2‐sided Student's *t*‐test (d, e). The *P*‐value < 0.05 was considered as significant difference.

Considering the importance of palmitoylation in the regulation of IRHOM2 stabilization, this forced us to identify the definite palmitoylated site of IRHOM2. With this function investigation, predicted positions of palmitoylation sites on IRHOM2 for *Homo sapiens* and *Mus musculus* were performed by conjoint analysis of GPS‐Palm software (MacOS_20 200 219) (The CUCKOO Workgroup, http://gpspalm.biocuckoo.cn/), and MDD‐Palm algorithm (http://csb.cse.yzu.edu.tw/MDDPalm/).^[^ ^26^, ^27^ ^]^ Both algorithms coincidentally predicted and provided the Top 4 palmitoylation sites for IRHOM2 with different confidence intervals and quality scores. Of note, cysteine 476 (C476) of human IRHOM2 and cysteine 448 (C448) of mouse Irhom2 in iRhom homology domain (IRHD) were predicted to be the most likely and reliable protein palmitoylation modification sites (**Figure** **2**a). Also, this cysteine residue site showed strongly conserved in different species (Figure 2b). To confirm the speculation, IRHOM2 protein remaining in IRHOM2 WT, C147A, C72A, C600A, and C476A mutants in human hepatocyte THLE2 cells, and Irhom2 WT, C117A, C572A, C571A, and C448A mutants in mouse primary hepatocytes were detected after CHX administration, respectively. Indeed, consistent with prediction, only C476A and C448A mutations exhibited a marked reduction in IRHOM2 abundance, while other mutations did not show significant alteration (Figure S2a,b, Supporting Information). The effects of a series of mutations on IRHOM2 remaining in indicated transfected hepatocytes were also confirmed by immunofluorescence (Figure S2c,d, Supporting Information). Meanwhile, the replacement of the C476 residue by alanine strictly blocked the palmitoylation of IRHOM2, as indicated by a the Click‐iT chemistry assay (Figure 2c). The C476A mutant dramatically reduced IRHOM2 protein expression profiles, while not influencing its mRNA levels (Figure 2d,e). Also, accelerated degradation of IRHOM2 (C476A) was significantly observed in the presence of CHX treatment in the time frame (Figure 2f), accompanied by a decrease in IRHOM2 remaining (Figure 2g). Given the determination of C476 as the key site for IRHOM2 palmitoylation, we wonder whether C_16_‐catched *S*‐palmitoylation primarily contributed to chemical modification for IRHOM2. Thus, a series of alkyl‐labeled fatty acylations including Alk14, Alk16, Alk18, and Alk20 were used to study this biological process. IRHOM2 can be marked effectively by palmitoylation (with Alk16) labels, but much less efficiently marked by labels with chain lengths of Alk14, stearoylation (Alk18) or Alk20 (Figure S3a,b, Supporting Information), which indicates that C_16_‐catched *S*‐palmitoylation is the major acyl groups for IRHOM2 chemical modification. Having identified the C476A and C448A mutation as the key site of IRHOM2 palmitoylation, we next examined the functional effects of these mutants on C_16_‐catched *S*‐palmitoylation over the course of alkynyl palmitic acid (Alk16) treatment. Expectedly, IRHOM2 with human C476A or mouse C448A mutation significantly abolished palmitoylation (with Alk16) labels, as supported by streptavidin pull‐down analysis, which revealed that human C476A or mouse C448A was required for *S*‐palmitoylation of IRHOM2 protein (Supplementary Figure S3c,d). Additionally, previous reports demonstrated that *S*‐palmitoylation contributed to targeted protein trafficking and cytomembrane translocation, and IRHOM2 reached the plasma membrane to function as an adaptor, recruitment, and shedding protein.^[^ ^13^, ^16^, ^21^, ^22^ ^]^ We accordingly investigated whether *S*‐palmitoylation affects IRHOM2 membrane translocation. IRHOM2 WT phenotype was mainly localized at the plasma membrane and endomembrane. Indeed, treatment of PAOA in THLE2 cells increased the membrane localization of IRHOM2 but decreased its nuclear localization (Figure S3e, Supporting Information), indicating that *S*‐palmitoylation accelerates the membrane localization of IRHOM2.

### 2.3 Palmitoylation‐Stabilized IRHOM2 Suppresses its Proteasome Degradation

**Figure 3:**
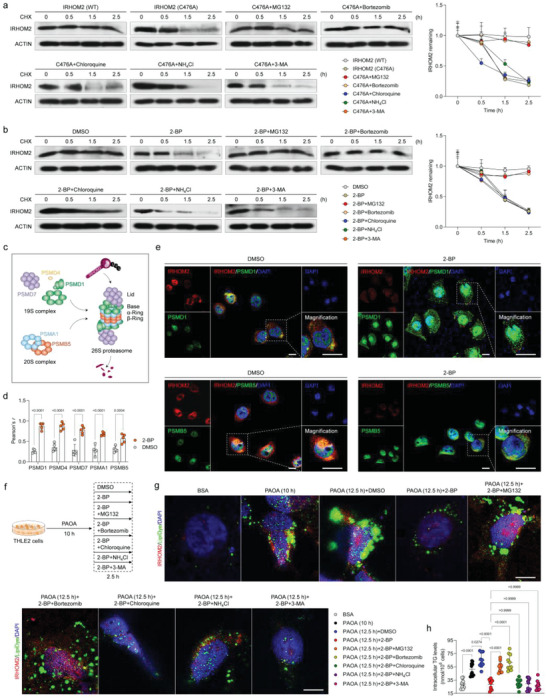
Palmitoylated IRHOM2 increases its endogenous retention by restraining proteasome signals. a) The THLE2 cells were transfected with IRHOM2 or IRHOM2 C476A mutant overexpression, followed by detection of IRHOM2 degradation under time‐gradient CHX treatment, in the presence of proteasome inhibitor (MG132, bortezomib), lysosome inhibitor (chloroquine, NH_4_Cl) or autophagy inhibitor (3‐MA). The right curve graph showing the relative IRHOM2 remaining ratio in the indicated time point (*n* = 4 per group). b) Time‐gradient CHX treatment detection showing the effects of 2‐BP on IRHOM2 degradation in THLE2 cells with/without different inhibitors. The right curve graph showing the relative IRHOM2 remaining level in the indicated time point (*n* = 4 per group). c) Sketch map showing the structure of the 26S proteasome and corresponding representative markers PSMD1, PSMD4 and PSMD7 for 19S complex, and PSMA1 and PSMB5 for 20S complex. d) Statistical data of the colocalization of IRHOM2 and PSMD1, PSMD4, PSMD7, PSMA1 and PSMB5 in THLE2 cells incubated with DMSO or 2‐BP (*n* = 5 per group; *P* < 0.05 versus DMSO group). e) Representative immunofluorescence pictures showing the colocalization of ectopically expressed IRHOM2 and specific marker of the assembled proteasome, PSMD1& PSMB5 in THLE2 cells treated with DMSO or 2‐BP. Scale bars, 50 µm. The white arrow indicates colocalization. f) Experimental design outline showing indicated treatment of different inducers in THLE2 cells. g, h) Representative immunofluorescence pictures showing the IRHOM2 expression (red) and lipid droplets formation (green) coexpression (g) in response to different treatments in indicated groups (*n* = 10 per group; *P* < 0.05 versus BSA group). Scale bars, 10 µm. The right scatter diagram shows the intracellular triglyceride (TG) contents in indicated groups (h). Data are expressed as mean ± SEM. The relevant experiments presented in this part were performed independently at least three times. Significance is determined by a 2‐sided Student's *t*‐test (d) or one‐way analysis of variance (ANOVA) followed by Dunnett's multiple comparisons test analysis (h). The *P*‐value < 0.05 was considered as significant difference.

Previous studies have revealed that the marked increase in IRHOM2 protein abundance dramatically abolished ubiquitination degradation in response to long‐term metabolic insults challenge.^[^ ^17^, ^18^ ^]^ We suspected that IRHOM2 may have a variety of different post‐translation modifications that synergistically or antagonistically determine its protein fate over the course of distinct physiological and pathological conditions. Accordingly, in these regards, we investigated the effects of palmitoylated IRHOM2 on its ubiquitin‐proteasome degradation progression. The facilitated degradation of palmitoylation of IRHOM2 with the C476A mutant could be blocked by the proteasome inhibitors MG132, bortezomib (**Figure** **3**a), and oprozomib (Figure S4a, Supporting Information), but not by the lysosomal inhibitors chloroquine, NH_4_Cl or the autophagy inhibitor 3‐methyladenine (3‐MA). To determine the proteasome‐dependent manner of progress, palmitoylation inhibitor 2‐BP was used to interrupt the endogenous IRHOM2 palmitoylation in THLE2 cells and primary hepatocytes (Figure 3b and Figure S4b, Supporting Information), ectopically expressed IRHOM2 (Figure S4c, Supporting Information) in primary hepatocytes, and evaluated the effects of a series of different indicated inhibitors on distinct degradation signals. Consistently, reduced stabilization of IRHOM2 induced by 2‐BP‐triggered depalmitoylation could be significantly abolished by MG132 and bortezomib, but not chloroquine, NH_4_Cl, 3‐MA, and Pepstatin A. Of note, IRHOM2 has been confirmed to be degraded by ubiquitin‐proteasome previously, thereby blocking its trafficking and translocation.^[^ ^17^, ^18^, ^19^ ^]^ To explore the functional effects of depalmitoylation on IRHOM2 cellular translocation, major elements for proteasome assembly including PSMD1, PSMD4, PSMD7, PSMA1, and PSMB5 (Figure 3c) were employed to investigate its colocalization changes with IRHOM2 over the course of depalmitoylation in IRHOM2. Unsurprisingly, administration of 2‐BP largely promoted the colocalization of IRHOM2 on the 26S proteasome, while upregulating its colocalization with 19S subunit and 20S subunit aggregation (Figure 3d,e and Supplementary Figure S4d). On the other hand, interruption of palmitoylated IRHOM2 with C476A or C448A mutant significantly increased the colocalization of IRHOM2 with proteasome (Figure S4e, Supporting Information). In addition, we also studied the effects of depalmitoylation‐associated proteasome degradation on lipid metabolism during metabolic stresses (PAOA) challenge (Figure 3f). Consistent with the above findings, destabilization of IRHOM2 mediated by 2‐BP was negatively correlated with lipid accumulation in PAOA‐treated cultured cells, as evidenced by decreased IRHOM2 expression and LipiDye (specific lipid droplet labeling) density. Reduced IRHOM2 abundance and lipid deposition could be markedly reversed by MG132 and bortezomib intervention, but not chloroquine, NH_4_Cl, and 3‐MA (Figure 3g,h). These data suggested that depalmitoylated IRHOM2 reduced its stabilization and accelerated proteasome‐related degradation progression.

### 2.4 ZDHHC3 is the Major Palmitoyltransferase for IRHOM2, and ZDHHC3 Activity is Positively Correlated with NASH Severity in Human and Rodent

**Figure 4:**
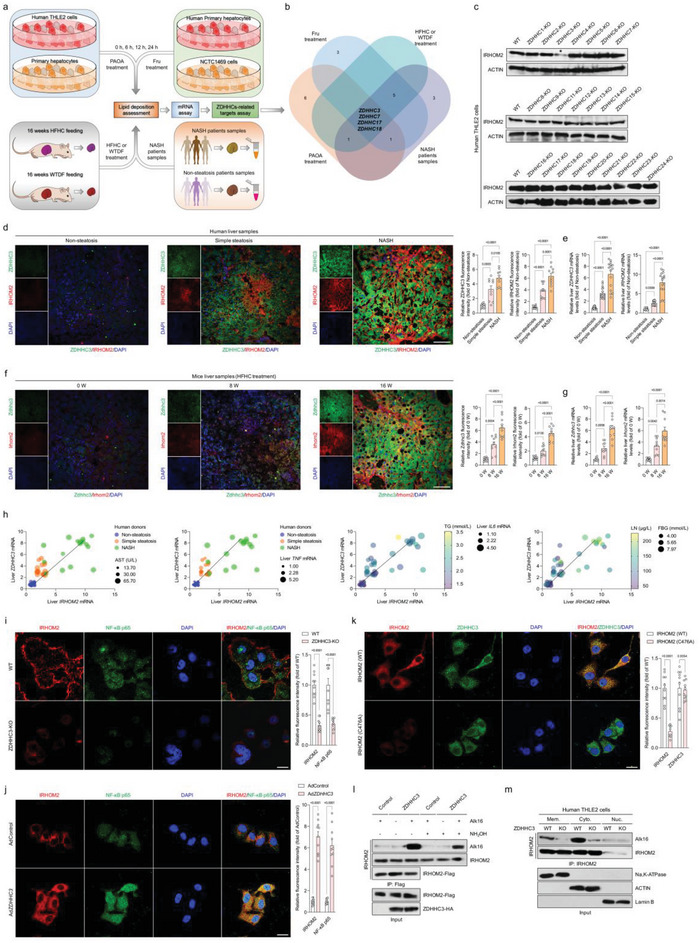
Hepatocyte IRHOM2 is palmitoylated by palmitoyltransferase ZDHHC3. a) Experimental design showing the protocol of identifying ZDHHCs‐related targets in response to a time‐course of 0.5 mM palmitic acid+1.0 mM oleic acid (PAOA) mixture, 5 mM fructose (Fru) in human THLE2 cells, mouse primary hepatocytes, human primary hepatocytes or 16‐weeks HFHC‐ or WTDF‐fed WT mice or in NASH patients samples. b) Venn diagram showing the Top 4 distinguishable expressed ZDHHC candidates in intersection of PAOA treatment group, HFHC‐/WTDF‐fed mice, Fru treatment group, and NASH patients samples. c) Representative western blotting bands showing the IRHOM2 expression changes in different ZDHHCs‐deleted THLE2 cells. The asterisk indicates the group with the most change among all the candidates (*n* = 4 per group). d, e) Representative immunofluorescence images of IRHOM2 and ZDHHC3 coexpression in lives of human donors with nonsteatosis phenotype, simple steatosis phenotype, and NASH phenotype with fluorescence intensity evaluation (d) (*n* = 10 samples per group; *P* < 0.05 versus nonsteatosis group), and corresponding liver *IRHOM2* and *ZDHHC3* mRNA expression profiles (e) (*n* = 10 samples for non‐steatosis phenotype; *n* = 17 samples for simple steatosis phenotype; *n* = 16 samples for NASH phenotype; *P* < 0.05 versus nonsteatosis group). Scale bars, 50 µm. f, g) Representative immunofluorescence images of Irhom2 and Zdhhc3 coexpression in lives of 0–16 weeks HFHC‐fed mice with fluorescence intensity evaluation (f) (*n* = 10 samples per group; *P* < 0.05 versus 0 week), and corresponding liver *Irhom2* and *Zdhhc3* mRNA expression profiles (g) (*n* = 10 samples; *P* < 0.05 versus 0 week). Scale bars, 50 µm. h) Pearson multiple correlation analysis for human subjects exhibiting the comprehensive correlation between liver *IRHOM2*, *ZDHHC3* mRNA expression and indicated parameter indexes (*n* = 49 indices per parameter). i) Representative immunofluorescence images of IRHOM2 and NF‐κB p65 coexpression in THLE2 cells with WT phenotype and ZDHHC3‐KO phenotype and corresponding fluorescence density analysis (*n* = 10 samples per group; *P* < 0.05 versus WT group). Scale bars, 50 µm. j) THLE2 cells were transfected with adenovirus‐loading ZDHHC3 overexpression vector (Ad*ZDHHC3*), followed by immunofluorescence analysis using IRHOM2 and NF‐κB p65 antibodies. Cells with empty adenovirus (AdControl) transfection served as a control group (*n* = 10 samples per group; *P* < 0.05 versus AdControl). Scale bars, 50 µm. k) The THLE2 cells with IRHOM2 WT or IRHOM2 C476A mutant transfection were incubated with IRHOM2 and ZDHHC3 antibodies, and corresponding fixed sections were then subjected to immunofluorescence analysis (*n* = 10 samples per group; *P* < 0.05 versus AdControl). Scale bars, 50 µm. l) The THLE2 cells were transfected with IRHOM2‐Flag and ZDHHC3‐HA vectors. Palmitoylated IRHOM2 levels were exhibited using Alk16 labeling in the presence or absence of hydroxylamine (NH_2_OH) administration. m) The wild‐type THLE2 cells or ZDHHC3‐deficient THLE2 cells were transfected with IRHOM2‐Flag, followed by labelling with Alk16. Subcellular fraction was collected and IRHOM2 protein levels were modulated to confirm that there were equal amount of IRHOM2 in the wild type and knockout cell component for input. Palmitoylated IRHOM2 levels in cell membrane (Mem.), cell cytoplasm (Cyto.), and cell nucleus (Nuc.) components were observed by immunoblotting analysis. Data are expressed as mean ± SEM. The relevant experiments presented in this part were performed independently at least three times. Significance is determined by one‐way analysis of variance (ANOVA) followed by Dunnett's multiple comparisons test analysis (e, g) or 2‐sided Student's *t*‐test (i‐k). The *P*‐value < 0.05 was considered as significant difference.

Having identified palmitoylation as a critical posttranslational modification of IRHOM2 in response to metabolic stresses challenge, we next inquired which palmitoyltransferase plays a major role in regulation of palmitoylated IRHOM2 in the setting of NASH. A series of in vivo and in vitro model‐simulated NASH pathological phenotypes were used to screen potential ZDHHC members (**Figure** **4**a). Indeed, in response to stimulants challenge, ZDHHCs‐related targets analysis of the top 4 distinguishable expressed candidates including ZDHHC3, ZDHHC7, ZDHHC17, and ZDHHC18 was highlighted at the intersection of these experiment groups (Figure 4b; Figure S5a,b). To further determine this, human hepatocyte THLE2 cells with *ZDHHC1*‐*ZDHHC24* knockout cells were established, respectively, to evaluate IRHOM2 protein expression profile changes. As expected, a significant decrease in IRHOM2 could be observed in *ZDHHC3*‐deficient cells, but not in the other ZDHHCs members (Figure 4c). A similar experimental phenomenon could also be exhibited in mouse primary hepatocytes with different adenovirus‐mediated Zdhhcs (Ad*Zdhhc*s) transfection (Figure S5c, Supporting Information). Consistently, contrastive analysis confirmed the expected IRHOM2 protein abundance alterations in the in vivo model with specific knockout or overexpression of *Zdhhc3* in hepatocytes, or in the in vitro model with adenovirus‐mediated *ZDHHC3* overexpression in THLE2 cells and mouse primary hepatocytes (Figure S5d–f, Supporting Information), as evidenced by the immunoblotting assay. Besides, the dynamic expression profiles of human liver samples and mice with 0–16 weeks of HFHC treatment suggested that IRHOM2 and ZDHHC3 protein and corresponding mRNA expression were gradually upregulated in the livers (Figure 4d–g). Also, the additional multiple Pearson correlation analysis for human patients with the NASH phenotype highlighted the role of ZDHHC3 in NASH progression, and its correlation with IRHOM2 and other NASH‐associated indicators (Figure 4h, Figure S5g,h, Supporting Information). Intriguingly, ZDHHC3 was identified as the major palmitoyltransferase for IRHOM2, which did affect not only IRHOM2 stabilization and palmitoylation over the course of ZDHHC3 deficiency or restoration, but also the NF‐κB p65 activation, a critical downstream cascade signal of IRHOM2 (Figure 4i,j, Figure S5i–k, Supporting Information). Coincidentally, the invalidation of palmitoylation by C476A decreased the co‐expression of IRHOM2 and ZDHHC3 (Figure 4k). Also, ectopically expressed ZDHHC3 in different hepatocytes elevated IRHOM2 protein expression and accelerated its cellular surface accumulation (Figure S5l,m, Supporting Information). Considering the determination of interaction between ZDHHCs and their targeted substrates, we inquired whether ZDHHC3 interacts with IRHOM2 in hepatocytes. Indeed, the Co‐IP assay indicated that direct binding between ZDHHC3 and IRHOM2 could be remarkably observed in the ectopic expression of human hepatocyte THLE2 cells (Figure S5n,o, Supporting Information). In addition, to further visualize IRHOM2 palmitoylation in the presence of ZDHHC3, similar to Figure S3, Supporting Information, we used Alk16 as a metabolic sign to examine the effects of ZDHHC3 on palmitoylated IRHOM2 and its cellular translocation. Incubation with NH_2_OH markedly suppressed palmitoylation levels on IRHOM2, indicating that upregulation of palmitoylated IRHOM2 mainly occurred on cysteine and was induced by ZDHHC3 (Figure 4l). Fractionation analysis in ZDHHC3 WT or *ZDHHC3*‐deficient THLE2 cells further suggested that ZDHHC3‐mediated palmitoylation of IRHOM2 elevated the amount of the modified IRHOM2 protein in the plasmolemma but not in the nuclear components (Figure 4m). The above findings consistently indicated that ZDHHC3 is a major palmitoyltransferase involved in the occurrence of palmitoylated IRHOM2, and its activity was positively correlated with NASH severity in human subjects and rodent models.

### 2.5 Dysfunctional ZDHHC3 Confers Protection Against NASH Progression

**Figure 5:**
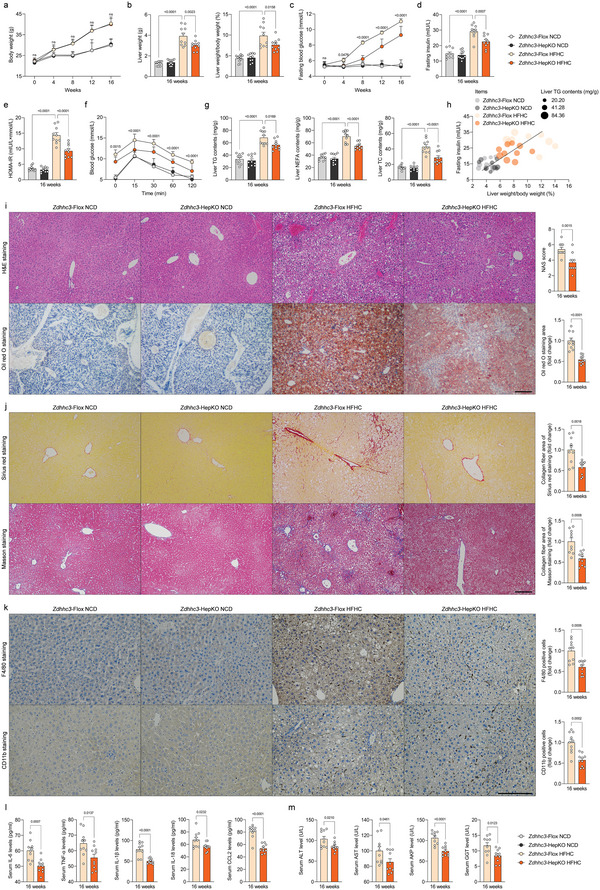
Hepatocyte‐specific loss of Zdhhc3 protects against HFHC‐induced NASH pathogenesis. a‐g) Records for the body weight (a), liver weight and the ratio of liver weight/body weight (%) (LW/BW) (b), fasting blood glucose levels (c), fasting insulin levels (d), HOMA‐IR index (e), glucose tolerance test (GTT) (f) and liver TG, NEFA and TC contents (g) of the HFHC‐fed *Zdhhc3*‐Flox mice and *Zdhhc3*‐HepKO mice; NCD diet‐fed mice were treated as corresponding control (*n* = 10 mice per group; *P* < 0.05 versus *Zdhhc3*‐Flox HFHC group). h) Pearson analysis indicating the correlations between fasting insulin, ratio of liver weight/body weight (%), and liver TG contents in indicated groups (*n* = 10 per parameter; *P* < 0.001 for all of these correlations). i‐k) Representative pictures of H&E staining, oil red O staining, histological NAS score (i) changes, sirius red staining, masson staining (j), and F4/80 and CD11b positive cells expression (k) in indicated groups (magnification, 100× for H&E staining, oil red O staining, masson staining and sirius red staining; magnification, 200× for F4/80 and CD11b staining; *n* = 10 images per group; *P* < 0.05 versus *Zdhhc3*‐Flox HFHC group). l, Records for inflammation‐related cytokines profiles including IL‐6, TNF‐α, IL‐1β, IL‐18 and CCL2 in serum from HFHC‐fed HepKO or Flox mice (*n* = 10 mice per group; *P* < 0.05 versus *Zdhhc3*‐Flox HFHC group). m, Records for liver function‐related indicators including ALT, AST, AKP, and GGT in serum from HFHC‐fed HepKO or Flox mice (*n* = 10 mice per group; *P* < 0.05 versus *Zdhhc3*‐Flox HFHC group). Data are expressed as mean ± SEM. The relevant experiments presented in this part were performed independently at least three times. Significance is determined by two‐way analysis of variance (ANOVA) followed by multiple comparisons test analysis (a‐g) or 2‐sided Student's *t*‐test (i‐m). The *P*‐value < 0.05 was considered as significant difference.

On top of being a significant palmitoyltransferase in regulation of IRHOM2 palmitoylation, we accordingly inquired whether ZDHHC3 would also contribute to NASH development in vivo. We then constructed hepatocyte‐specific *Zdhhc3*‐deficient mice (*Zdhhc3*‐HepKO) (Figure S6a–d, Supporting Information), followed by 16‐week HFHC diet feeding. The increases in pan‐palmitoylation levels were markedly enhanced by the HFHC challenge in livers from WT mice, but mitigated by *Zdhhc3* deletion in livers from transgenic mice compared to NCD‐administrated WT mice or HFHC‐fed Flox mice (Figure S6e, Supporting Information). Consistently, the *Zdhhc3*‐HepKO mice exhibited lower liver weights and LW/BW ratios than those in HFHC‐treated *Zdhhc3*‐Flox mice (**Figure** **5**a,b). Also, the *Zdhhc3*‐HepKO mice displayed lower fasting blood glucose, fasting insulin, and its corresponding HOMA‐IR index (Figure 5c–f) than control mice. The HFHC‐treated *Zdhhc3*‐HepKO mice further had less hepatosteatosis and hepatocellular injury than those in HFHC‐treated *Zdhhc3*‐Flox mice, as indicated by hepatic TC, NEFA, and TG levels (Figure 5g), Pearson correlation analysis (Figure 5h), H&E staining and oil red O staining (Figure 5i). According to Sirius red staining and Masson staining analysis, liver collagen accumulation was lower in liver samples from HFHC‐fed *Zdhhc3*‐HepKO mice than in control mice (Figure 5j). Consistently, hepatic inflammation was also significantly decreased in *Zdhhc3*‐deleted mice compared to Flox mice, as evidenced by positive CD11b‐ and F4/80‐associated inflammatory cell infiltration in liver samples (Figure 5k). The serum contents of pro‐inflammatory cytokines (i.e., IL‐6, TNF‐α, IL‐1β, IL‐18, and CCL2), hepatic function indicators (i.e., ALT, AST, AKP, and GGT) (Figure 5l,m) and liver gene expression profile alterations associated with inflammation (Figure S7a, Supporting Information), fatty acid metabolism (Figure S7b, Supporting Information), and profibrotic genes (Figure S7c, Supporting Information) were markedly downregulated by *Zdhhc3*‐HepKO mice in the context of NASH.

Of note, the pathogenesis of NASH is complicated, and is regarded as a heterogeneous disease, the pathogenesis of which is mediated by both external environmental factors and individual genetic determinants, which cannot be fully replicated in animal models.^[^ ^28^, ^29^ ^]^ Thus we further studied ZDHHC3 function on a WTDF diet containing 15% w/v fructose‐drinking water‐mediated NASH model that as a complementary model simulates NASH pathogenesis. In line with the findings obtained in HFHC‐fed *Zdhhc3*‐HepKO mice, indeed, the deficient mice did not render marked changes in body weight, but displayed lower liver weight and LW/BW ratio during the 16‐week WTDF diet challenge (Figure S7d, Supporting Information). In addition, the abnormal fasting blood glucose, fasting insulin contents, HOMA‐IR index, hepatic lipid contents (i.e., TG, NEFA, and TC), as well as increased NAS score, inflammation, collagen accumulation, and hepatic function indicators (i.e., ALT, AST, AKP, and GGT) (Figure S7e–r, Supporting Information) were also markedly mitigated by *Zdhhc3*‐HepKO mice, compared to controls. In light of significant upregulation of ZDHHC3 in fatty liver, consistent with the method of Supplementary Figure S1d,e, we next constructed a special in vitro model using adenovirus‐mediated ectopic expression to knockdown or overexpress ZDHHC3 in human or mouse hepatocytes, and investigated the function of ZDHHC3 in regulating the key hallmarks of lipid accumulation and inflammation. The transfected mouse primary hepatocytes were treated with PAOA, or medium supplemented with serum harvested from NASH patients (NASH serum) or nonsteatosis subjects (Nonsteatosis serum) for 24 h, respectively. Unsurprisingly, the Nile red staining assay and intracellular TG contents determination suggested that both PAOA‐ and NASH serum‐treated primary hepatocyte lipid deposition in Adsh*Zdhhc3* groups were markedly downregulated compared to those in Nonsteatosis serum or BSA controls, and were followed by decreased inflammation‐ and lipid synthesis‐related gene expression profiles (Figure S8a–f, Supporting Information). Also, consistent results could be observed in ectopically expressed human hepatocyte THLE2 cells during PAOA or NASH serum challenge (Figure S8g–l, Supporting Information). These findings indicated that liver functional loss of ZDHHC3 mitigated hepatic lipid dysregulation, inflammation, and collagen deposition, resulting in improvement of steatohepatitis and early hepatic fibrosis in mice.

Having the positive relevance of ZDHHC3 expression with the severity of NASH in mice model with *ZDHHC3* loss‐of‐function experiments, ground on *Rosaβgeo26* conditional and/or inducible transgenesis(hereunder named as Rosa*^Zdhhc3^*) (Figure S9a,b, Supporting Information). As, expected, the upregulation of the pan‐palmitoylation contents were significantly promoted by hepatocyte‐specific *Zdhhc3* overexpression compared to the *Zdhhc3*‐HepNTG mice after HFHC diet consumption for 16 weeks (Figure S9c, Supporting Information). Also, the Rosa*^Zdhhc3^* mice injected with AAV8‐TBG‐Cre were used to specifically overexpress Zdhhc3 in hepatocytes (*Zdhhc3*‐HepTG), followed by 16‐week HFHC diet administration (Figure S10a, Supporting Information). In contrast to *Zdhhc3*‐HepKO mice, the liver weight and LW/BW of the *Zdhhc3*‐HepTG mice were significantly increased, however, there was no statistically significant difference in body weight compared to *Zdhhc3*‐HepNTG groups (Figure S10b, Supporting Information). Furthermore, insulin levels, fasting blood glucose levels, HOMA‐IR index, hepatic TG, NEFA and TC contents, hepatosteatosis, inflammation, and fibrosis‐related indicators were concurrently upregulated by Zdhhc3 overexpression (Figure S10b–j, Supporting Information), as evidenced by serum biochemical analysis, H&E staining, oil red O staining, Masson staining, Sirius red staining and inflammatory infiltration with F4/80 & CD11b positive cells assay. The dysregulated liver metabolism, lipid deposition, hepatosteatosis, and profibrosis, followed by elevated pro‐inflammatory cytokines and abnormal liver functional indicators were further observed by *Zdhhc3*‐HepTG over the course of HFHC‐induced NASH phenotype (Figure S10k,l, Supporting Information). Inversely, in line with the similar protocols mentioned in Figure S8, Supporting Information, overexpression of ZDHHC3 induced by adenovirus in THLE2 cells and mouse primary hepatocytes, a significant increase in lipid deposition, and inflammation in vitro upon challenge with PAOA or NASH serum could be visualized by ectopically overexpressed ZDHHC3 cells (Figure S11a–h, Supporting Information). These results revealed that ZDHHC3 is an effective accelerator of NASH and its associated metabolic disorder in mouse model. The ZDHHC3 targeting could be an effective way to mitigate NASH progression.

### 2.6 IRHOM2 is Required for ZDHHC3 Targeting Function Over the Course of NASH Pathogenesis

**Figure 6:**
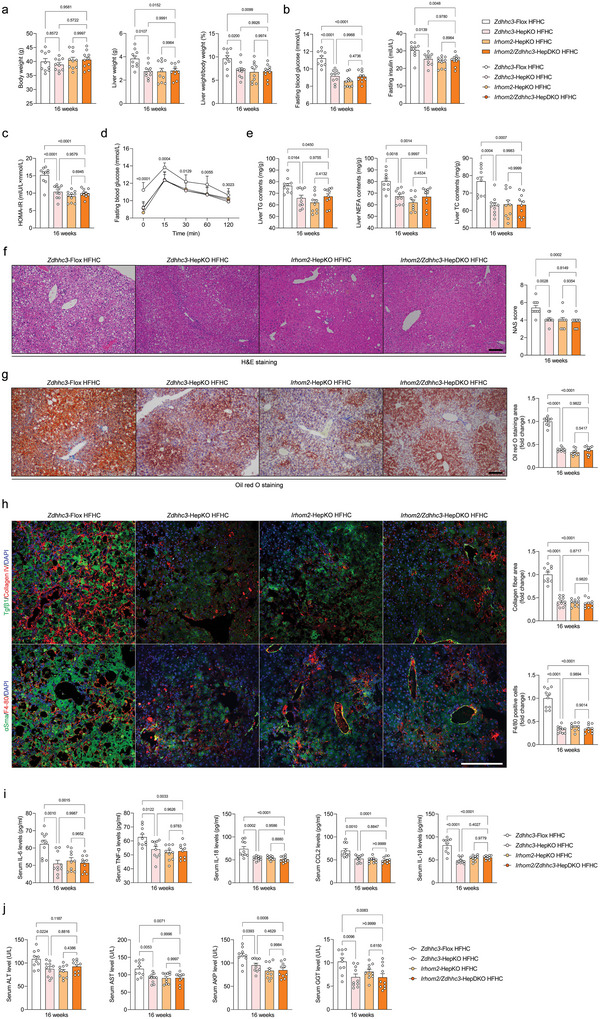
IRHOM2 is required for ZDHHC3 function over the course of NASH pathogenesis. a‐d) Records for body weight, liver weight, and liver weight/body weight ratio (%) (a), fasting blood glucose levels, fasting insulin levels (b), HOMA‐IR index (c), and glucose tolerance test (GTT) analysis (d) of the *Zdhhc3*‐Flox mice, *Zdhhc3*‐HepKO mice, *Irhom2*‐HepKO mice, and *Irhom2*/*Zdhhc3*‐HepDKO mice after 16‐weeks HFHC diet challenge (*n* = 10 mice per group; *P* < 0.05 versus *Zdhhc3*‐Flox HFHC group). e) Liver lipid contents including TG, TC, and NEFA in the indicated group (*n* = 10 mice per group; *P* < 0.05 versus *Zdhhc3*‐Flox HFHC group). f,g) Representative pictures of H&E staining (f), Oil red O staining (g), and corresponding NAS score in an indicated group (magnification, 100×; *n* = 10 images per group for each staining). h) Representative pictures of immunofluorescence analysis of Tgfβ & Collagen IV, and αSma & F4/80 coexpression, respectively (*n* = 10 images per group; *P* < 0.05 versus *Zdhhc3*‐Flox HFHC group). Scale bars, 100 µm. i,j) Records for serum pro‐inflammatory cytokines IL‐6, TNF‐α, IL‐18, CCL2, and IL‐1β contents (i) and liver function indicators including serum ALT, AST, and AKP contents (j) in indicated groups (*n* = 10 mice per group; *P* < 0.05 versus *Zdhhc3*‐Flox HFHC group). Data are expressed as mean ± SEM. The relevant experiments presented in this part were performed independently at least three times. Significance is determined by one‐way analysis of variance (ANOVA) followed by Dunnett's multiple comparisons test analysis. The *P*‐value < 0.05 was considered as significant difference.

Considering the potent accelerated effects of ZDHHC3 on NASH development and its related pathological processes, the above data forced us to investigate the molecular mechanisms of ZDHHC3 and its inherent function. On the basis of confirmation of IRHOM2 as a potential target and substrate of ZDHHC3, we next established hepatocyte‐specific *Zdhhc3* and *Irhom2* dual deficiency mice (*Irhom2*/*Zdhhc3*‐HepDKO), as described in the Methods section. As expected, *Irhom2* deletion completely displaced *Zdhhc3* ablation‐triggered improvement of steatohepatitis in the context of HFHC‐induced NASH phenotype. Importantly, all the NASH pathological symptoms that were alleviated by *Zdhhc3* deficiency including elevated liver weight gain, glucose abnormality, liver lipid accumulation, increased liver pro‐inflammatory cytokines, proinflammation‐related gene expression profiles, liver collagen deposition, and hepatocellular injury were also significantly observed in *Irhom2*/*Zdhhc3*‐HepDKO mice (**Figure** **6**a–j). Furthermore, to deeply reveal the functional effects of Zdhhc3 relying on palmitoylated Irhom2 in the setting of NASH pathogenesis in mice, we performed additional experiments using AAV‐TBG‐*Irhom2* (mutation)‐mediated hepatocytes specific Irhom2 protein expression restoration in *Irhom2*/*Zdhhc3*‐HepDKO mice (Figure S12a, Supporting Information). The *Irhom2/Zdhhc3*‐HepDKO mice were co‐injected with AAV‐TBG‐*Irhom2* (WT)+AAV‐TBG‐*Zdhhc3* or AAV‐TBG‐*Irhom2* (C448A)+AAV‐TBG‐*Zdhhc3* to restore Irhom2 (WT)+Zdhhc3 or Irhom2 (C448A)+Zdhhc3 protein expression, followed by administration of HFHC diet for 16 weeks. Unsurprisingly, compared to mice with AAV‐TBG‐*Irhom2* (WT)+AAV‐TBG‐*Zdhhc3* restoration groups, gain‐of‐function of Irhom2 (C448A)+Zdhhc3 in mice failed to exert their acceleration over the course of HFHC‐induced NASH pathogenesis. Irhom2 (C448A) and Zdhhc3 corestoration in hepatocytes did not upregulate LW/BW ratio, increase dysregulated glucose metabolism, lipid deposition contents, serum pro‐inflammatory cytokines production and liver enzymes levels, compared to Irhom2 (WT) and Zdhhc3 co‐injected mice during NASH development, suggesting that Irhom2 (C448) was indeed the primary site of palmitoylation modification in *Mus musculus*, and Irhom2 with this mutated site directly lost its promoting effect on NASH progression and functioned as a catalytic substrate of Zdhhc3 (Figure S12b–j, Supporting Information). On the other hand, consistent with other results, the role of Zdhhc3 in metabolic regulation was strongly correlated with Irhom2. Thus, the effects of ZDHHC3 on exacerbation of steatohepatitis largely depend on its ability to positively regulate IRHOM2.

**Figure 7:**
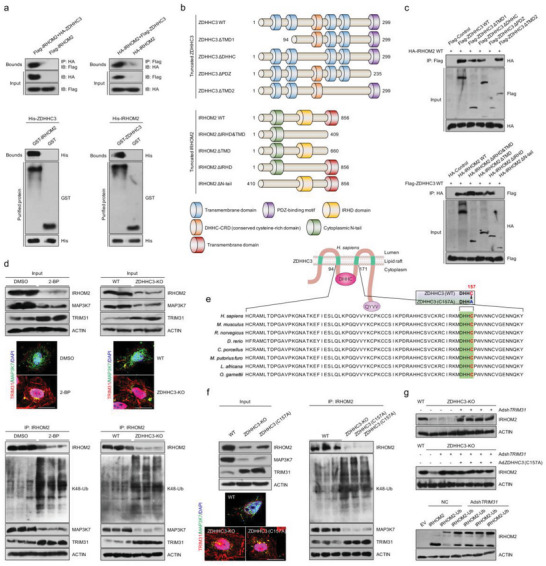
Palmitoylation‐stabilized IRHOM2 suppresses its ubiquitination and TRIM31‐triggered degradation. a) The THLE2 cells were transfected with indicated vectors for following coimmunoprecipitation (Co‐IP) assay. Co‐IP detection of the interaction of IRHOM2 with ZDHHC3 in THLE2 cells transfected with Flag‐IRHOM2 and HA‐ZDHHC3 or HA‐IRHOM2 and Flag‐ZDHHC3 plasmids. Immunoblotting probed with anti‐HA or anti‐Flag antibody (upper). Representative immunoblotting bands for GST precipitation showing ZDHHC3‐IRHOM2 binding by treating purified His‐ZDHHC3 with purified GST‐IRHOM2 or by treating His‐IRHOM2 with purified ZDHHC3‐GST in vitro (lower). Purified GST was regarded as a control. b,c) Schematic of human full‐length and truncated ZDHHC3 and IRHOM2 (b), and representative western blotting mapping assay indicating the interaction domains of ZDHHC3 and IRHOM2 (c). d) IP detection in THLE2 cells showing the effects of palmitoylation inhibitor 2‐BP challenge and ZDHHC3 deficiency on the binding between IRHOM2 and other interested proteins with known function, and representative immunofluorescence images of TRIM31 and MAP3K7 coexpression (upper), and K48‐linked ubiquitination of IRHOM2 (lower) (*n* = 5 images per group). Scale bars, 10 µm. e) ZDHHC3 structure showing the catalytic activity motif mutant (C157A) of DHHC domain in different species. f) IP detection in THLE2 cells showing the effects of ZDHHC3 knockout and ZDHHC3 with C157A mutant on the binding between IRHOM2 and IRHOM2 downstream signaling factors, i.e., MAP3K7 and TRIM31, and representative immunofluorescence images of TRIM31 and MAP3K7 coexpression (left), and K48‐linked ubiquitination of IRHOM2 (right) (*n* = 5 images per group). Scale bars, 10 µm. g) Immunoblotting assay showing the involvement of TRIM31 in depalmitoylation‐triggered proteasome degradation of IRHOM2 in ZDHHC3‐deleted THLE2 cells (upper) or ZDHHC3 (C157A)‐transfected THLE2 cells (middle) (*n* = 4 samples per group). THLE2 cells expressing IRHOM2 or IRHOM2‐Ub were transfected with Adsh*TRIM31*, followed by a specific antibody for IRHOM2 used to detect the IRHOM2 or IRHOM2‐Ub expression. Negative control (NC) or empty vector (EV) were used as control. This experiment was repeated three times.

Given the accelerated role of ZDHHC3 in regulating IRHOM2 signal, the above obtained in vivo and in vitro results forced us to study another critical concern, whether and how ZDHHC3 interacted with IRHOM2 in the setting of NASH. In line with prime binding analysis in Figure S5n,o, Supporting Information, following Co‐IP and GST pulldown assays indicated that exogenous expression of ZDHHC3 did interact with IRHOM2 and vice versa, in transfected THLE2 cells (**Figure** **7**a). Interaction analysis also exhibited that the PDZ domain of ZDHHC3 was essential for binding to IRHOM2. The Co‐IP analysis with corresponding ZDHHC3 mutants further revealed that ZDHHC3 without PDZ domain lost its ability to interact with IRHOM2. Thus, the PDZ domain of ZDHHC3 contributed to the protein interaction with IRHOM2 (Figure 7b,c).

### 2.7 Palmitoylation‐Stabilized IRHOM2 Suppresses its Ubiquitination Degradation Triggered by TRIM31

To further clarify how palmitoylation regulates proteasome‐related degradation of IRHOM2, in light of the tight relationship between IRHOM2 and its partner functional proteins, we accordingly investigated its effect on the interaction between IRHOM2 and a series of proteins directly involved in IRHOM2. Previous reports have suggested that 14‐3‐3, FRMD8, and STING contributed to IRHOM2 trafficking through binding to cytoplasmic N‐terminal of IRHOM2, thereby partly sustaining its protein stabilization.^[^ ^13^, ^14^, ^15^ ^]^ A tripartite motif‐containing protein TRIM31 was confirmed to be an E3 ubiquitin ligase targeting IRHOM2 ubiquitin‐proteasome degradation and critical suppressor of IRHOM2‐MAP3K7 signal axis in our previous works.^[^ ^11^, ^17^ ^]^ Also, the C476 site was identified as a key palmitoylation site of IRHOM2 in our above experiments. Therefore, we blocked palmitoylation of IRHOM2 by C476A mutant, and examined the interaction with 14‐3‐3, FRMD8, and STING by Co‐IP of IRHOM2. Surprisingly, no marked alterations were observed for these protein‐protein bindings (Figure S13a, Supporting Information). The suppression of palmitoylated IRHOM2 via 2‐BP or *ZDHHC3* deletion resulted in a significant decrease in MAP3K7 (a critical downstream signal of IRHOM2) and an increase in TRIM31 (a definite E3 ubiquitin ligase for IRHOM2 degradation) protein abundance (Figure 7d). These results revealed that depalmitoylation of IRHOM2 with the C476A mutant did not affect its interaction with other partner proteins, but it could affect the IRHOM2‐MAP3K7 signal and its ubiquitination degradation process. To better explain this issue, interactions between IRHOM2 and more partner factors in the presence of ZDHHC3 were analyzed by Co‐IP assay. In line with the above findings, except for binding to reduced TRIM31, we did not observe remarkable changes in the interaction between IRHOM2 and other candidates (Figure S13b, Supporting Information). The additional Co‐IP assay also indicated that dysregulated palmitoylation of IRHOM2 with C476A mutant was capable of increasing its ability to interact with TRIM31 (Figure S13c,d, Supporting Information). These subsection results consistently suggested that palmitoylation‐stabilized IRHOM2 by ZDHHC3 interrupted IRHOM2‐TRIM31 binding, whereas destabilized IRHOM2 with C476A mutant could markedly lose its ability to interact with TRIM31.

Of note, palmitoylation has been shown to coordinate targeted proteins stabilization by obstructing ubiquitination‐related degradation,^[^ ^20^ ^]^ and our recent work demonstrated that IRHOM2 could be delivered to K48‐linked polyubiquitination modification, therefore increasing proteasome degradation.^[^ ^17^ ^]^ Thus, we inquired whether ubiquitination of IRHOM2 could be interrupted by ZDHHC3‐mediated palmitoylation, followed by MAP3K7 signaling hyperactivation. As expected, the reduction of palmitoylation by 2‐BP dramatically upregulated the ubiquitination of IRHOM2 through K48 linkage, and the ablation of ZDHHC3 in THLE2 cells led to a similar observation (Figure 7d). Moreover, to accurately investigate the manner of ZDHHC3‐affected IRHOM2 ubiquitination, the ubiquitin mutants vectors K48O, K63O, K33O, K27O, K29O, and K11O were used for in vitro transfection assay. Interestingly, PAOA‐triggered reduction in ubiquitination of IRHOM2 could be markedly found in the presence of a K48‐loading vector, but not with other vectors in both the transfected THLE2 cells (Figure S13e, Supporting Information) and Adsh*Zdhhc3*‐transfected mouse primary hepatocytes (Figure S13f, Supporting Information). Subsequently, given the important role of ZDHHC3 in the regulation and catalyzation of palmitoylation, we screened amino acid sequences of the DHHC domain of ZDHHC3 in various species and underlined a very conserved site ZDHHC3 (C157) in this domain. Indeed, in THLE2 cells, ZDHHC3 with C157A mutant did lose its catalytic function in suppression of K48‐linked IRHOM2 ubiquitination and MAP3K7 activation (Figure 7e,f and Figure S13g, Supporting Information), and this process of ubiquitination was also significantly downregulated by Adsh*TRIM31* or *TRIM31* knockout (Figure 7g andFigure S13h, Supporting Information). The above results did reveal that C157 site in DHHC domain of ZDHHC3 is required for its biological catalytic function in modulating IRHOM2 palmitoylation. In addition, since IRHOM2 C476A/C448A mutant or ZDHHC3 C157A mutant exhibited marked effects on IRHOM2 palmitoylation and its cellular translocation in the presence of metabolic stresses, we accordingly inquired whether palmitoylated IRHOM2 may influence its interaction with IRHOM2. Therefore, in response to PAOA challenge, cells were transfected with indicated vectors as described in Figure S13i–k, Supporting Information, followed by treatment with recombinant human or mouse IRHOM2 chimeric protein that accessed immunofluorescence visual labeling. Expectedly, blockade of palmitoylated IRHOM2 by its C476A/C448A mutant or ZDHHC3 C157A mutant preponderantly reduced IRHOM2 binding on the plasma membrane distribution, while restoration of ZDHHC3 WT was capable of reversing these observations. The above consequences revealed that ZDHHC3‐stabilized IRHOM2 palmitoylation dramatically interrupted its ubiquitination‐proteasome degradation induced by TRIM31.

### 2.8 ZDHHC3 Accelerates NASH Progression by Activating IRHOM2‐MAP3K7 Signaling

Having the tight correlation of IRHOM2 destabilization with catalytic function‐associated DHHC domain activity of ZDHHC3, to further confirm whether ZDHHC3 with C157A mutant in DHHC domain was required for the accelerated role of ZDHHC3 in the setting of NASH, we then subjected AAV‐TBG‐*Zdhhc3*, AAV‐TBG‐*Zdhhc3* (C157A), or AAV‐TBG‐Blank to *Zdhhc3*‐HepKO mice, accompanied by 16‐weeks HFHC‐triggered NASH model in vivo (Figure S14a,b, Supporting Information). Indeed, except for the mice injected with AAV‐TBG‐*Zdhhc3*, groups injected with AAV‐TBG‐*Zdhhc3* (C157A), DHHC domain mutants, were not capable of promoting NASH diet‐triggered progression of steatohepatitis compared to those in corresponding controls, as evidenced by biochemical and immunohistochemistry analysis (Figure S14c–g, Supporting Information). Additionally, after 16‐weeks HFHC administration, the inflammation‐associated indicators, collagen accumulation, hepatic function indicators, and aberrant lipid metabolism‐related gene expression profiles did not display upregulation by AAV‐TBG‐*Zdhhc3* (C157A) injection, compared with AAV‐TBG‐*Zdhhc3* groups (Figure S15a–d, Supporting Information). Also, *Zdhhc3* with C157A mutant restoration in DHHC domain did not promote steatohepatitis by activating liver Irhom2‐Map3k7 signaling axis, and did not exhibit a marked change compared with *Zdhhc3*‐HepControl HFHC mice groups (Figure S15e, Supporting Information).

Consistent with findings from AAV‐TBG‐*Zdhhc3* (C157A) injection in vivo, *ZDHHC3*‐deficient THLE2 cells transfected with Ad*ZDHHC3*, Ad*ZDHHC3* (C157A) or empty controls were accordingly employed to restore ZDHHC3 expression in the additional in vitro assay. Undoubtedly, cells with Ad*ZDHHC3* (C157A) expression failed to facilitate lipid deposition during PAOA treatment compared with Ad*ZDHHC3* expression groups (Figure S16a, Supporting Information). Also, increased lipid synthesis‐related gene expression profiles and activated IRHOM2‐MAP3K7 signaling axis could not be significantly observed in Ad*ZDHHC3* (C157A)‐transfected THLE2 cells (Figure S16b–d, Supporting Information). These additional consequences further revealed that the evolutionary conserved C157 site of DHHC domain in ZDHHC3 is required for its functional effects on the regulation of NASH pathogenesis.

### 2.9 Pharmacological *S*‐Palmitoylation Inhibition Mitigates NASH phenotype in mice and rabbits

After demonstrating the role of ZDHHC3 in the aggravation of steatohepatitis and its potential molecular mechanisms, competitive inhibition was possibly regarded as an efficient strategy for targeting indicated enzymes. Since no detailed therapeutic strategies targeting palmitoyltransferase have been widely reported, with the palmitoylated IRHOM2 identified, we inquired whether 2‐BP, a well‐characterized suppressor of protein *S*‐palmitoylation, was capable of protecting against steatohepatitis in mice and rabbit model. To this end, we treated WT mice with 0, 2.5, 10.0, and 20.0 mg kg^−1^ 2‐BP, i.h., every 2 days during 16‐week HFHC diet‐mediated NASH pathogenesis (Figure S17a, Supporting Information). As expected, HFHC‐fed mice with long‐term 2‐BP treatment did show lower liver weights and LW/BW ratio than those without 2‐BP treatment mice, and this phenomenon was in a 2‐BP dose‐dependent manner (Figure S17b, Supporting Information). Also, the concentration gradient of 2‐BP‐treated mice exhibited lower fasting blood glucose, fasting insulin, and its corresponding HOMA‐IR index (Figure S17c, Supporting Information) than control mice. Intervention with 2‐BP further had less hepatosteatosis and hepatocellular injury than those in 0 mg kg^−1^ 2‐BP‐treated mice, as indicated by liver TC, NEFA, and TG levels (Figure S17d, Supporting Information), H&E staining, and oil red O staining (Figure S17e, Supporting Information). On the basis of co‐expression immunofluorescence analysis, liver collagen deposition presented a significant gradient reduction in liver samples between 0, 2.5, 10.0, and 20.0 mg kg^−1^ 2‐BP‐administrated mice (Figure S17f and Figure S18a,b, Supporting Information). Consistently, the serum contents of pro‐inflammatory cytokines, hepatic function indicators, activated liver Zdhhc3, and a significant increase in pan‐palmitoylation contents along with Irhom2‐Map3k7 signaling axis were markedly suppressed by dose‐dependent 2‐BP treatment in the context of NASH (Figure S18c–f, Supporting Information). As an additional complementary experiment, we treated WT rabbits with 40 mg kg^−1^ 2‐BP, *i.p*., every 2 days upon 8‐weeks HFHC diet‐induced NASH pathogenesis (Figure S17g, Supporting Information). In line with the observations made in the above mice model with NASH, 2‐BP treatment did not alter rabbits body weight, but as expected from dose‐dependent 2‐BP‐treated mice, it far lowered serum free fatty acid (FFA) levels (Figure S17h, Supporting Information), abnormal serum lipid contents (i.e., TG, TC, LDLC, and HDLC) (Figure S17i, Supporting Information), dysregulated blood glucose and insulin levels (Figure S17j, Supporting Information). On top of that, rabbits that were treated with 2‐BP had a substantial decrease in liver lipid deposition and hepatic dysfunction, a marked improvement in liver NAS score, TG, TC contents, and a remarkable reduction in AST, ALT, AKP and hydroxyproline levels after an 8‐week HFHC diet challenge (Figure S17k–m, Supporting Information). These proof‐of‐principle works echo results from our above genetic mice models with NASH, indicating that, again, the ZDHHC3‐IRHOM2 axis is central to the progression of NASH, and thus targeting hepatocyte ZDHHC3 and development of ZDHHC3‐based specific drugs may have the ability to retard NASH progression and its associated complications.

## 3 Discussion

Metabolism dysregulation‐associated liver disease develops commonly in excessive energy intake‐triggered obesity and type 2 diabetes (T2D), and initially identifies as NAFLD which can dramatically boost this course to NASH pathological phenotypes.^[^ ^3^, ^4^, ^5^ ^]^ Ultimately, it has to be accepted that our lack of understanding and undervaluation of the complex pathogenesis of NASH are the main reasons for the failure of innovation and the slow development of NASH clinical therapeutic drugs and corresponding therapeutic strategies.^[^ ^30^ ^]^ Also, a comprehensive and insightful understanding of molecular pathogenesis and pathological principles of NASH, as well as the search for potential therapeutic targets and effective drugs, are undoubtedly becoming the top priorities in NASH treatment and the focus of global scholars. Therefore, relevant research hotspots have entered the molecular target point‐breaking stage. Recently, a sequence of ZDHHC members has received increasing attention owing to its special palmitoylation‐related enzymatic catalytic function.^[^ ^31^ ^]^ Our current study highlighted 4 of the most dramatically changed palmitoyltransferases (i.e., ZDHHC3, ZDHHC7, ZDHHC17, and ZDHHC18) in different models in vivo or in vitro upon metabolic insult challenge. IRHOM2, although not an absolutely perfect hallmark for NASH pathogenesis, predisposes to hepatosteatosis, early liver fibrosis, and even hepatocellular carcinoma (HCC), and was previously treated as an important influencing factor that served as an inflammatory promoter in the long‐term mortality of patients with NASH. Also, we revealed a critical role of palmitoylation in regulating the stabilization of IRHOM2 protein, which is associated with the suppression of an inherent ubiquitin‐proteasome protein degradation signal of IRHOM2. ZDHHC3 further exhibited promoted effects on NASH progression by activating IRHOM2 and its downstream MAP3K7‐JNK cascade signaling, thereby increasing hepatosteatosis, inflammation, and collagen accumulation. Admittedly, upregulated chronic inflammation action is the primary pathway for NASH progression, however, the process of endogenous suppression of inflammation‐associated signaling via blockers may be quite different. This study extends and suggests candidates for possible pharmacological targets, conferring potential experimental evidence for NASH treatment and drug development.

Studies during the past several decades on preclinical drugs and therapeutic strategies for NAFLD/NASH treatment have been extensively investigated.^[^ ^7^, ^8^, ^9^, ^10^ ^]^ With the complicated NASH pathogenesis identified as an ingravescent liver disease with increasing insulin resistance, hepatosteatosis, glucose intolerance, and liver inflammation, a series of potential targeted drugs in preclinical or clinical trials did not meet the expected requirements. Recently, IRHOM2, also called as RHBDF2, a key member of rhomboid family, acts as a critical initiator over the course of NASH by directly recruiting MAP3K7 signal to hyperactivate downstream MAP3K7‐JNK and NF‐κB p65 cascade pathways.^[^ ^11^, ^12^, ^17^ ^]^ Excessive high‐energy diet intake did significantly upregulate IRHOM2 protein expression, and increased IRHOM2 also exhibits a positive correlation with NASH severity in human and rodents. However, in response to metabolic stresses challenge, elevated amplitude of intracellular IRHOM2 markedly suppressed ubiquitination degradation, followed by the promotion of IRHOM2 trafficking and membrane distribution. The distinct phenomena indicate that IRHOM2 may have a variety of different chemical modifications that synergistically or competitively regulate NASH progression by other unknown factors and molecular mechanisms. Indeed, at many posttranslational modification forms, palmitoylation has been exhibited to modulate protein stabilization by blocking ubiquitination, our recent studies have determined a series of endogenous indicators (i.e., TRIM31 and USP13) that promoted the ubiquitinated or deubiquitinated IRHOM2 and the resulting blockade of downstream cascade signaling, leading to mitigation of animal models with severe NASH pathological phenotypes.^[^ ^17^, ^18^ ^]^ With this regard, we confirmed for the first time that deterioration of NASH pathogenesis could be facilitated by increased IRHOM2 abundance through upregulation of *S*‐palmitoylation, accompanied by suppression of TRIM31‐triggered ubiquitin‐proteasome degradation of IRHOM2.

Zinc finger DHHC‐type palmitoyltransferase 3 (ZDHHC3), a critical member of Zinc finger‐DHHC (Aspartate‐histidine‐histidine‐cysteine)‐CRD (Cysteine rich domain)‐type palmitoylacyltransferases, is regarded as a “Janus‐faced” factor involved in immune response, nervous diseases, metabolism, tumorigenesis and development by catalyzing palmitoylation modification of targeted protein, leading to targeted substrate trafficking or translocation.^[^ ^32^, ^33^, ^34^, ^35^ ^]^ Previous reports have indicated that increased ZDHHC3 was capable of promoting metabolic diseases‐related cognition impairment during high‐fat diet administration, and the additional studies further found that ZDHHC3 could also mediate T helper cell differentiation‐related colitis or cancer cells growth by pro‐inflammation signaling pathway, leading to more severe pathological phenotypes.^[^ ^22^, ^32^, ^33^ ^]^ Meanwhile, ZDHHC3 was positively correlated with tumorigenesis, and deletion of ZDHHC3 conferred anti‐tumor cell activities via upregulating oxidative stress, thereby reducing cell proliferation and tumor growth.^[^ ^26^ ^]^ Of note, PD‐L1‐triggered immune evasion in tumor could be significantly observed by ZDHHC3 in vivo and in vitro.^[^ ^33^ ^]^ These findings collectively suggested that ZDHHC3 served as its distinct function in different diseases, even tissue or cell types. On top of being a marked promoter in these diseases, our current study did provide a novel role for ZDHHC3 in NASH development by targeting IRHOM2 palmitoylation. In animal models with NASH, ZDHHC3 performed acceleration of pro‐inflammation, steatosis, and early fibrosis formation by increasing *S*‐palmitoylation‐stabilized IRHOM2 along with decrease in proteasome degradation of IRHOM2. The aggravated effects of ZDHHC3 on IRHOM2 and its downstream signaling cascade tightly depend on its DHHC domain activity. ZDHHC3 activity is positively correlated with IRHOM2 abundance, upregulated inflammation, and serum lipid levels in both human subjects with NASH and rodent NASH model. In keeping with these findings, mice with *Zdhhc3* deficiency exhibited a decreased severity of liver injury. While deactivation of other ZDHHC3 substrates involved in inflammation (e.g., STAT3, GluA1, and STING) may also contribute to the reduced inflammatory action we detect with ZDHHC3 ablation, none of these targets is indicated to dramatically suppress inflammation as ZDHHC3 does. Notably, we also determined that IRHOM2 is a direct and novel interaction protein partner of ZDHHC3. ZDHHC3 accelerated IRHOM2 expression by reducing its degradation via upregulating palmitoylation‐stabilized IRHOM2, followed by suppressing TRIM31‐mediated ubiquitination of K48 linkage, further promoting IRHOM2‐MAP3K7 signaling hyperactivation and downstream events. In the meantime, prolonged over‐nutrition intake not only promotes ZDHHC3 expression and IRHOM2‐MAP3K7 signaling activation, but also accelerates lipid accumulation in hepatocytes. On the other hand, the formed intracellular lipid (TG) pools, in turn, provide more 16‐carbon saturated fatty acids as special feed‐stock for ZDHHC3 to catalyze IRHOM2 palmitoylation. This process ultimately creates a vicious cycle and positive feedback that accelerates NASH progression.

In addition, ZDHHC3 exerts its catalytic function at the molecular levels by primarily relying on specific DHHC domain.^[^ ^37^ ^]^ Evidence‐based studies have suggested that the DHHC domain of ZDHHCs is evolutionary conserved and is required for interaction with the potential targeted proteins. Actually, in our work, PDZ domain of ZDHHC3 is obligatory for interaction with IRHOM2, while the C157 site of DHHC domain in ZDHHC3 is responsible for enzymatic activity and catalytic function. Moreover, the importance of the N‐terminal of IRHOM2 for its binding to other partner factors has been widely determined. It was not clear, however, whether the iRhom homology domain (IRHD) of IRHOM2 region had other functions beyond the known forward trafficking for TNF‐α shedding and EGF receptor signaling. Interestingly, cysteine 476 (C476) of human IRHOM2 IRHD domain is predicted to be the most likely and reliable protein palmitoylation modification site by algorithm analysis. Indeed, in the presence of ZDHHC3, IRHOM2 with C476A mutant still normally interacts with its known partner proteins including 14‐3‐3, FRMD8, and STING, but it markedly increases direct interaction with TRIM31, followed by a decrease in IRHOM2‐MAP3K7 signal activation, and increase in K48‐linked ubiquitination‐proteasome degradation of IRHOM2. These also show that the C476 site of IRHOM2 is necessary for ZDHHC3‐induced palmitoylation. The mutation reduces the stability of IRHOM2, and blocks the function of ZDHHC3 to use IRHOM2 as the main catalytic substrate, ultimately leading to enhanced degradation of IRHOM2. Besides, as a well‐characterized suppressor of protein *S*‐palmitoylation, 2‐bromopalmitate (2‐BP) has been exhibited to have anti‐inflammatory effects in mitigation of neuropathic pain and bone cancer pain. However, the role of 2‐BP in inhibiting the development of NASH remains unknown. Our additional results further uncover that in response to HFHC diet challenge, 2‐BP can be beneficial to the mitigation of pathological symptoms of NASH at appropriate doses, which also provides the possibility for the development of 2‐BP as a drug with potential pharmacological efficacy in the treatment of NASH via targeting palmitoylation.

**Figure 8:**
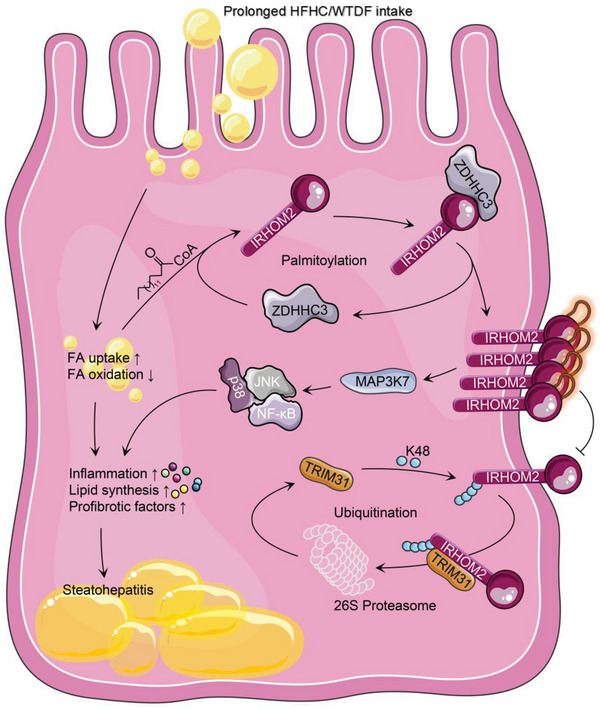
Palmitoylation maintains IRHOM2 by intercepting its ubiquitination and proteasome degradation. Working model of the regulation of palmitoylated IRHOM2 by palmitoyltransferase ZDHHC3 retards ubiquitin‐proteasome degradation of IRHOM2. Prolonged HFHC or WTDF intake dramatically increases circulating free fatty acid levels and palmitate (C16:0) to promote formation of palmitoylated IRHOM2. Elevated palmitoylation of IRHOM2 by ZDHHC3 facilitates its membrane localization and accumulation, and stabilization. Excessive IRHOM2 abundance significantly recruits its downstream signaling MAP3K7‐JNK‐NF‐κB p65 axis hyperactivation, thereby accelerating steatohepatitis progression in response to high‐energy diet challenge. In the meantime, palmitoylation‐stabilized IRHOM2 positively blocks its ubiquitination and TRIM31‐mediated proteasome degradation via decreasing ubiquitin of K48 linkage. Targeting palmitoylation using a pharmacological inhibitor (2‐BP) provides a potential therapeutic strategy to regain IRHOM2 ubiquitination and proteasome degradation.

In summary, as shown in **Figure** **8**, our current study provides a new insight into steatohepatitis pathogenesis and targeting IRHOM2 palmitoylated regulation by over‐nutrition intake‐associated signals, and proposes a novel molecular vicious circle mediated by ZDHHC3‐IRHOM2 axis and involving posttranslational regulation of IRHOM2, potentially further linking metabolic syndrome and its complications. These findings offer a possibly novel target for NASH treatment and theoretical guidance on drug development for this worldwide disease.

## 4 Experimental Section

### Study Design and Ethical Statement

The goal of this study was to 1) identify the correlation of ZDHHC3 expression in livers with severity of NASH in patients, 2) identify what role of ZDHHC3 activity alteration plays in NASH‐related lipid deposition, inflammation, and fibrosis progress by combining a series of dietary rodent models of NASH with functional loss of *ZDHHC3* and gain‐of‐function transgenic expression mice, 3) determine the role of *ZDHHC3*‐regulated IRHOM2 palmitoylation in regulation of NASH pathogenesis, and 4) palmitoylation‐stabilized IRHOM2 suppresses its ubiquitin‐proteasome degradation triggered by TRIM31. The whole experimental protocols regarding animals used in this study were permitted by the *Guide for the Care & Use of Laboratory Animals* (8^th^ edition NIH, in Chinese) and permitted by the *Institutional Animal Use &Care Committee (IACUC)* in Chongqing University of Education (20200018CQUE). The approaches and procedures involved in current work were used in line with the *Regulations of the People's Republic of China on the Administration of Experimental Animals (Revised & Exposure Draft)*, issued by the *Ministry of Science and Technology of the People's Republic of China* (http://www.most.gov.cn).

### Human Donors Liver Samples

Human donors’ liver specimens were harvested from adult donors with NAFLD who underwent biopsy tissue samples or liver transplantation samples. The relevant nonsteatotic liver tissues were obtained from donors who were not eligible for liver transplantation for nonliver reasons. Nonsteatosis samples (*n* = 16), Simple steatosis samples (*n* = 17), and NASH phenotype liver samples (*n* = 16) were obtained and included in this study. Of note, steatotic liver samples from patients with any of the following conditions were excluded from the study: excessive drinking (alcohol >70 g for females or alcohol >140 g for males, per week), viral infection, or drug abuse (including hepatitis B & C virus infection). Also, prior to this study, the samples of nonsteatosis and simple steatosis in this cohort were collected from patients without taking statins or insulin. The NASH phenotype liver samples (*n* = 16) in this cohort were from patients who had taken pioglitazone (15–30 mg per day) for no more than 24 months. Liver sample donors and their families agree & sign written informed consent. Physiological characteristics of patients and hepatic injury‐related serology are shown in Table S1, Supporting Information. All protocols involving human donors in this work were grounded on *the Ethical Principles for Medical Research Involving Human Subjects, Declaration of Helsinki (64th WMA general assembly)*, and totally approved by the *Academic Committee of Experimental Animal Ethics, Use &Care Union* in Chongqing University of Education and other participating units.

### Antibodies, Reagents, and Chemicals

Corresponding primary antibodies used in the study and against the following indicated proteins were purchased from Abcam Inc: anti‐ACTIN (#ab179467, 1/2500 dilution), anti‐IRHOM2 (#ab116139, 1/250‐1/1000 dilution), anti‐ZDHHC3 (#ab31837, 1/150‐1/1000 dilution), anti‐p38 (#ab182453, 1/1000 dilution), anti‐phosphorylated p38 (#ab4822, 1/1000 dilution), anti‐NF‐κB p65 (#ab32536, 1/1000 dilution), anti‐phosphorylated NF‐κB p65 (#ab76302, 1/1000 dilution), anti‐F4/80 (#ab16911, 1/100 dilution), anti‐ubiquitin (linkage‐specific K48) (#ab140601, 1/200‐1/1000 dilution), anti‐Ub (#ab134953, 1/200‐1/1000 dilution) and anti‐Lamin B1 (#ab229025, 1/1000 dilution). Antibodies of anti‐MAP3K7 (#4505, 1/1000 dilution), anti‐phosphorylated MAP3K7 (#9339, 1/1000 dilution), anti‐JNK (#9258, 1/1000 dilution), anti‐phosphorylated JNK (#4668, 1/1000 dilution), anti‐HA (#3724, 1/250‐1/1000 dilution), anti‐Myc (#2276, 1/250‐1/1000 dilution), anti‐Flag (#14 793, 1/250‐1/1000 dilution), anti‐His (#12 698, 1/250‐1/1000 dilution) were obtained from Cell Signaling Technology(CST). The additional antibodies including anti‐TGF‐beta 1 (#NBP1‐80289, 1/200 dilution), anti‐alpha‐SMA (#NB300‐978, 1/200 dilution), anti‐Collagen IV (#NBP1‐26549, 1/200 dilution), anti‐TRIM31 (#NBP2‐88468, 1/200‐1/600 dilution), anti‐14‐3‐3 (#NBP2‐27202, 1/1000 dilution), anti‐FRMD8 (#H00083786‐B01P, 1/1000 dilution), anti‐STING (#NBP2‐48684, 1/1000 dilution) and anti‐Na, K‐ATPase (#H00000483‐B01P, 1/1000 dilution) were purchased from Novus Biologicals, LLC. Moreover, antibody against IRHOM2 (#orb386934, 1/200‐1/1000 dilution, Biobyt) were also used in the current study.

The Novagen BCA protein quantification kits (#712 853, MilliporeSigma) was used to determine samples’ protein concentration. The HRP‐tagged secondary antibodies (Thermo Fisher Scientific) with 1/10000‐1/15000 dilution was used for visualization in western blotting analysis. The palmitate (PA) (#HY‐N0830), oleic acid (OA) (#HY‐N1446), MG132 (#HY‐13259), fructose (#HY‐N7092), cycloheximide (#HY‐12320), dimethylsulfoxide (#HY‐Y0320), ABD957 (#HY‐142161), 3‐methyladenine (#HY‐19312), bortezomib (#HY‐10227), chloroquine (#HY‐17589A) and pepstatin A (#HY‐P0018) were purchased from MedChemExpress (MCE), Shanghai, China. Biotin picolyl azide (#900 912), alkynyl myristic acid (Alk14) (#1164), alkynyl palmitic acid (Alk16) (#1165), and alkynyl stearic acid (Alk18) (#1166) were obtained from Click Chemistry Tools. The alkynyl arachidic acid (Alk20) were synthesized and produced in our lab (>98% purity). The 2‐bromopalmitate (2‐BP) (#21 604), palmostatin B (Palm B) (#178 501), NH_4_Cl (#A9434), and BSA (#V900933) were obtained from Merck KGaA. The palmostatin M (Palm M) (#565 407) was purchased from MedKoo Biosciences, Inc. TaqMan Universal PCR Master Mix (#P/N4304437), and PowerUp SYBR Green (#A25742) were purchased from Applied Biosystems. The LipiDye Lipid Droplet Green Kit (#FDV‐0010) was obtained from Whatman.

The specific antibody for pan palmitoylation detection was produced according to previous studies and patent (CN106153941).^[^ ^33^, ^38^, ^39^ ^]^ Briefly, to produce antibody targeting pan palmitoylation, a short peptide with only 2 Cys C‐C (pal) (98.5%), in which 1 cysteine was palmitoylated, synthesized, and used as the hapten according to previous protocols and procedures.^[^ ^33^, ^38^, ^39^ ^]^ Meanwhile, the obtained peptide C‐C (pal) was conjugated with keyhole limpet haemocyanin (KLH) as the additional antigen to immunize New Zealand white rabbits. Anti‐serum was harvested after 3 doses of immunization. Use ammonium sulfate precipitation to purify this anti‐serum before using. To better verify this antibody, we have used several additional verification strategies in accordance with the previous report.^[^ ^33^ ^]^ In brief, according to the thioester bonds between the protein and palmityls, free sulfhydryl groups can be produced by the hydroxylamine buffer liquid. Thiopropylsepharose 6B (TS‐6B) resin (a kind of thiol‐reactive group‐containing resin) was used to purify the hydroxylamine buffer‐incubated palmitoylated valosin‐containing (transitional endoplasmic reticulum ATPase) protein, followed by immunoblotting assay with anti‐valosin‐containing protein antibody. On the other hand, the obtained antibody was further used to observe the small c‐terminal domain phosphatase 1 (SCP1) palmitoylation, as described in the previous study.^[^ ^39^ ^]^ Additionally, in this work, this ready‐made antibody was used to observe the palmitoylation of proteins collected from cells treated with different compounds such as Palm B, Palm M, or 2‐BP. All the above protocols synergistically supported the sensitivity and specificity of the antibody to the proteins palmitoylation reaction.

### Cell Culture and Administration

Human THLE2 (#CRL‐2706), HepG2 (#HB‐8065), and NCTC1469 (#CCL‐9.1) cells were purchased from the American Type Culture Collection (ATCC). Isolation and protocols for the human primary hepatocytes culture were performed according to our previous reports.^[^ ^17^, ^18^ ^]^ All cell lines associating with corresponding in vitro or in vivo experiments were compulsorily examined for *mycoplasma* interference via polymerase chain reaction detection, and then cultured in indicated corresponding medium. The cell lines were respectively cultured in Dulbecco's Modified Eagle Medium (Cat: BC‐M‐005, Bio‐Channel Biotechnology Co., Ltd., China) or Roswell Park Memorial Institute (RPMI) 1640 medium (Cat: G4534, Servicebio, Wuhan, China) containing 1% penicillin+streptomycin (Cat: BL505A; Biosharp Life Sciences), 10% premium quality FBS (Cat: 085–150, WISENT), and were maintained in a 5% CO_2_, 37 °C directly‐heated type cell incubator (SANYO). Mouse primary cultured hepatocytes used in this experiment were isolated and concentrated from indicated experiments’ animals by liver perfusion. In brief, after anesthesia with sodium pentobarbital, mice abdominal cavity was opened. Then, the liver samples were tardily perfused with 1×liver perfusion working solution (Cat: 17 701 038, Gibco) and 1×liver digest working solution (Cat: 17 703 034, Gibco) via the portal vein. Then, the digested liver tissue was filtered using a 100 µm steel mesh. The primary isolated hepatocytes were produced by harvesting the filter solution after 800 rpm centrifugalization (4 °C, 5–10 min), and next purified using percoll‐solution (Cat: 40501ES60, YEASEN, Shanghai, China). The isolated hepatocytes were maintained in corresponding DMEM medium (10% FBS & 1% penicillin‐streptomycin) and then cultured in a 37 °C, 5% CO_2_ condition. To mimic hepatic lipid deposition and steatosis of in vivo experiments, the THLE2 cells, NCTC1469 cells, human primary hepatocytes, and mouse primary isolated hepatocytes were administrated with the indicated dose of PAOA or fructose to investigate the ZDHHCs protein expression and lipid accumulation.

### Construction of the Knockout Cell and Knockdown Lines

The establishment method of targeted gene‐deletion cell lines that participated in this study were constructed as described previously.^[^ ^17^, ^29^ ^]^ Briefly, THLE2 cell lines with *ZDHHC1‐ZDHHC24* ablation were established by CRISPR‐Cas9 system. The designed small guide RNA (sgRNA) for human *ZDHHCs* gene were established and cloned into lentiCRISPR v2 vectors(Addgene, Watertown, MA, USA) to create the Cas9/sgRNA‐loaded lentivirus. Small guide RNA (sgRNA) for establishment of knockout THLE2 cells were obtained from Santa Cruz Biotechnology, Inc., as described in Table S2, Supporting Information. Oligonucleotide for the sgRNAs were packaged into lentiCRISPR v2 vectors digested with BsmBI restriction enzyme. The obtained cell clones‐containing gene deficiency were distinguished by western blotting. Mouse primary hepatocyte with *Zdhhc3* deletion cells were directly isolated from global *Zdhhc3*‐deficient mice. For the establishment of mouse primary hepatocytes with *Zdhhc1‐Zdhhc25* knockdown, short hairpin RNA (shRNA) for knockdown expression of *Zdhhcs* were obtained from Santa Cruz Biotechnology, Inc., as described in Table S3, Supporting Information, were packed into adenovirus (Addgene, Watertown, MA, USA), respectively.

### Vectors Preparation and Transfection

To overexpress *ZDHHC3*, *Homo sapiens* full‐length *ZDHHC3* cDNA expression vectors were constructed by PCR‐based cDNA amplification, and then packed into the pcDNA3.1 3×Flag‐tagged plasmids and the pcDNA3.1 3×HA‐tagged plasmid (Addgene, Watertown, MA, USA), respectively.Abridged ZDHHC3 and IRHOM2 tiny fragments expression plasmids including Flag‐ZDHHC3 WT, Flag‐ZDHHC3 △TMD1, Flag‐ZDHHC3 △DHHC, Flag‐ZDHHC3 △PDZ, Flag‐ZDHHC3 △TMD2, HA‐IRHOM2 WT, HA‐IRHOM2 △IRHD&TMD, HA‐IRHOM2 △TMD, HA‐IRHOM2 △IRHD and HA‐IRHOM2 △N‐tail were accordingly prepared. The ubiquitin with Myc‐tag expression plasmids were prepared ground on pcDNA3.1 plasmid. Next, ubiquitin was then inserted into the pcDNA3.1 Myc‐tag vector (Addgene, Watertown, MA, USA). Vectors were transfected into THLE2 cells via ViaFect Transfection Reagent (Cat: E4982, Promega, Beijing, China) according to the product specification. Moreover, to further study the biological effects of ZDHHC3 in vitro, we have constructed corresponding adenovirus (Ad)‐packaged targeted gene expression plasmids. *Homo sapiens* full‐length *ZDHHC3* sequences and corresponding ready‐made short hairpin RNA (shRNA) targeting human *ZDHHC3* (sh*ZDHHC3*), adenovirus expressing shRNA for silencing of *Mus musculus Zdhhc3*, human WT *ZDHHC3* sequences with C157A mutant, *Homo sapiens* full‐length *IRHOM2* and *TRIM31* sequences, and adenovirus expressing ready‐made shRNA for silencing of *Mus musculus Irhom2* and *Trim31* were respectively packaged into adenovirus by Adeno‐X Adenoviral System 3 Kit (Cat: 632 269, Takara Bio Inc.). The empty adenovirus (AdshRNA or AdGFP) was regarded as controls for expression suppression (knockdown) or overexpression, respectively. The achieved adenovirus (Ad) vectors were purified and titrated to 5 × 10^10^ PFU by Vivapure AdenoPACK (Cat: VS‐AVPQ022, Sartorius, Shanghai, China) according to the product specification.

### Metabolic Factors and Serum Cytokines Parameters

Triglyceride (TG) contents were quantified using Triglyceride (TG) Content Detection Kit (Cat: D799795‐0050, Sangon Biotech, Co., Ltd., Shanghai, China) or Triglyceride (TG) Assay Quantification Kit (Cat: KA0847, Novus Biologicals) based on the product specification. The contents of blood glucose after the experimental period were examined using universal glucose test strips (glucose oxidase method) (Lot: 3 558 469, ONETOUCHUltra, LifeScan, Inc., CA, USA). Homeostasis Model Assessment for Insulin Resistance (HOMA‐IR) index was quantified from fasting insulin and fasting blood glucose, respectively. The HOMA‐IR index was exhibited and calculated as following formula: [HOMA‐IR = fasting insulin × fasting blood glucose/22.5]. The GTT detection protocols were performed according to our recent reports.^[^ ^17^, ^18^ ^]^ Determination of mouse chemokines and cytokines were performed using corresponding commercially‐available ELISA kits. The TNF‐α (Cat: ab100747), IL‐1β (Cat: ab197742), IL‐6 (Cat: ab222503), CCL2 (Cat: ab208979), IL‐18 (Cat: ab216165) and insulin (Cat: ab285341) ELISA kits were purchased from Abcam. The contents of serum or tissue glutamic pyruvic transaminase (GPT/ALT) (Cat: C009‐2‐1), glutamic oxalacetic transaminase (GOT/AST) (Cat: C010‐1‐1), alkline phosphatase (ALP/AKP) (Cat: A059‐1‐1), total cholesterol (TC) (Cat: A111‐1‐1), hepatic triglyceride (TG) (Cat: A110‐1‐1), γ‐Glutamyl Transferase (GGT) (Cat: C017‐1‐1) and nonesterified fatty acids (NEFA) (Cat: A042‐2‐1) were quantified by commercially‐available kits (Nanjing Jiancheng Bioengineering Institute, China) in the indicated experiment’ groups based on the manufacturer's instructions. Liver hydroxyproline and collagen were detected using Hydroxyproline Assay Kit (Cat: MAK008, Sigma‐Aldrich), Collagen Assay Kit (Cat: MAK322, Sigma‐Aldrich) and Soluble Collagen Quantification Assay Kit (Cat: CS0006, Sigma‐Aldrich) according to the product instructions.

### Histopathological Analysis

To perform histopathologic and immunohistochemical assay, the tissue was consequently fixed with 4% formaldehyde‐tissue fixative solution (Cat: 80 096 618, Sinopharm Chemical Reagent Co., Ltd., China), embedded in paraffin wax (Cat: 69 018 961, Sinopharm Chemical Reagent Co., Ltd., China), and then sectioned transversely. The tissue slices were subjected to H&E staining (Cat: G1120, Hematoxylin‐Eosin/HE Staining Kit, Solarbio Life Sciences, Beijing, China) to visualize the degree of lipid accumulation and hepatic inflammation. To further analyze hepatic lipid deposition, the slices were frozen in tissue optimum cutting temperature (O.C.T)‐freeze medium (Cat: C1400, Applygen Technologies, Inc., China) and then subjected to oil red O Kit (Cat: PH1226, Scientific Phygene, China) for 10–15 min. After being washed with 60% isopropanol (Cat: 40 064 360, Sinopharm Chemical Reagent Co., Ltd., China), the tissue slices were re‐stained with haematoxylin. Moreover, to visualize collagen contents in liver tissue, slices were subjected to masson staining (Cat: abs9348, Masson's Trichrome Stain Kit, Absin, Shanghai, China) and sirius red staining (Cat: PH1099, Enhanced Sirius Red Staining Kit, Scientific Phygene, China). To perform immunohistochemical analysis, paraffin‐embedded slices were dewaxed prior to incubation with indicated primary antibodies at 4 °C refrigeration overnight. The corresponding anti‐rabbit IgG, anti‐mouse IgG, or anti‐goat IgG antibodies were used as the secondary antibody (Thermo Fisher Scientific). All histological experiments were performed according to standard protocols presented in the reagent instructions and operation manual, and were performed by three histologists blinded to treatment procedures. Pictures were displayed using a light microscope (Leica, Germany) for samples section detection and a confocal laser microscopy system (FV3000, Olympus, Japan) for immunofluorescence section detection.

### Glutathione S‐Transferase (GST) Pull‐Down Detection

Direct protein binding between ZDHHC3 and IRHOM2 was performed using the GST pull‐down analyses. The MagneGST Pull‐Down System Kit (#V8870, Promega, Beijing, China) was used to detect the protein binding in this regard of the experiment. Briefly, the *Rosetta*‐DE3 competent cells were transformed with the plasmid pGEx‐4T‐1/GST‐*ZDHHC3* or pGEx‐4T‐1/GST‐*IRHOM2* and then induced vectors expression by treating with 0.5 m Misopropyl β‐D‐thiogalactoside (IPTG) (Cat: HY‐15921, MedChemExpress, Shanghai, China). Then, the extractions were co‐treated with corresponding GST particles for 1 h, 4 °C. The GST particles were then coincubated with His‐tagged *ZDHHC3* or His‐tagged *IRHOM2*, which were provided by immunoprecipitation (IP) for the additional 5 h. Interacting proteins were eluted in elution buffer followed by western blotting analysis using an anti‐Flag antibody. The *Rosetta*‐DE3 competent cells expressing only GST‐tag were regarded as the control.

### In Vivo *and* In Vitro Binding Ubiquitination Detection

The in vivo or in vitro binding ubiquitination detection were respectively operated in the current experiments according to the previous protocols and methods.^[^ ^17^, ^18^, ^29^ ^]^ Ubiquitination were investigated with a VIVAlink Ubiquitin Kit (Cat: VB2952‐50, Viva Bioscience, Exeter, UK) following procedures and protocols of the product manual.

### RNA Extraction, Quality Control, and Quantitative PCR (qPCR) Assay

The whole RNA from indicated liver tissue or cells were separated by TRNzol Universal agentia (Cat: DP424, TIANGEN^®^, Beijing, China) according to protocols recommended by product specification. The obtained RNA were kept at −80 °C for no more than 14 days. The absorption of RNA contents at 260 nm was confirmed using a Nanodrop photometer (Tecan) analysis. The best RNA purity was guaranteed by confirmation of the 260 nm/280 nm adsorption ratio (values >2.00). Next, 1 µg of extracted RNA was inverse transcripted using a universal RT‐PCR Kit (M‐MLV) (#RP1100, Solarbio Life Sciences, Beijing, China) and TaqMan Universal PCR Master Mix (#P/N 4 304 437, Applied Biosystems). The inverse transcription procedure was carried out at 42 °C for 1 h, followed by enzyme inactivation at 70 °C for 10 min. PCR process was carried out using PowerUp SYBR Green (#A25742, Thermo Fisher Scientific) and SensiMix SYBR Master Mix Kits (#QP100001, OriGene Technologies) in ABI PRISM 7900HT systems (Applied Biosystems). The specific primer sequences for lipid metabolism, inflammation, and fibrosis‐related key genes were produced by Sangon Biotech (Shanghai, China) or ready‐made primers obtained from OriGene Technologies, Inc. The fold difference values were counted based on the 2^(‐ΔΔ^ *^C^_t_* ^)^ expression, where Δ*C_t_* represents the difference in cycle thresholds between GAPDH and the target gene, and ΔΔ*C_t_* represents the relative alteration in the differences between indicated experimental groups and control groups. The sequences of primer were included in Table S4, Supporting Information.

### Immunoblotting Detection

To perform immunoblotting analyses, liver tissue or cells were subjected to RIPA (radio immunoprecipitation assay) lysis buffer (Cat: PH0317, Scientific Phygene) to yield lysates. Then, the final supernatant was compressed by centrifugation at 4°C, 13 000 rpmfor 30 min. Protein concentration of obtained supernatant was confirmed by BCA Protein Quantification Kit with fat‐free BSA as a control. The total extracted protein samples were then processed to immunoblotting assay. The same amounts of total protein isolated from the indicated cells or liver samples were processed to 10% or 12% SDS/PAGE gel (Cat: 20328ES50, SDS/PAGE Gel Preparation Kit, YEASEN, Shanghai, China) and then subjected to a 0.45 µM Immun‐Blot polyvinylidene fluoride (PVDF) membrane (Cat: 10 600 023, AmershamHybond, GE Healthcare Life Science, Germany) via wetting transfer method, followed by western blotting using the assigned primary antibodies. Subsequently, the immunoblotting membranes were incubated with blocking buffer (5% nonfat‐dried milk) (Cat: LP0033B, Biosharp Life Science, Beijing, China) in 1×TBS working buffer solution (Cat: PH1402, Scientific Phygene) containing 0.1% Tween‐20 (Cat: 9005‐64‐5, Sinopharm Chemical Reagent Co., Ltd., China) (1×TBST working buffer solution) for 1 h, and mingled with the assigned primary antibodies at 4 °C refrigeration overnight. Then, the PVDF membranes were washed in 1×TBST working buffer solution for 3 times, followed by cotreated with horseradish peroxidase‐tagged goat anti‐rabbit IgG (H+L) or anti‐mouse IgG (H+L) (Cat: 33201ES60; Cat: 33101ES60, YEASEN, Shanghai, China) for 1 h–2 h at 25 °C—30 °C. Immunoblotting membranes were visualized by New‐SUPER (Hypersensitivity Type) ECL Kit (Cat: KGP1128, KeyGenBioTECH, China) and exposed to FUJI Medical X‐ray film (Cat: 4 741 023 952, FUJIFILM, China). Corresponding protein levels were then calculated as gray‐scale score(Version 1.8.0, Microsoft Windows, Image J, NIH, USA) and normalized to GAPDH, and standardized as a fold change of controls.

### Labelling, Click Chemistry, Palmitoylation Identification, and Streptavidin Pulldown

With this experiment and procedures, the Click‐iT Palmitic Acid, Azide Kit (Cat#: C10265), Click‐iT™ Protein Reaction Buffer Kit (Cat#: C10276), and Click‐iT Cell Reaction Buffer Kit (Cat#: C10269) was purchased from Thermo Fisher Scientific, and then used to perform click chemistry and identification of palmitoylated IRHOM2 according to product instruction. After the indicated transfection of IRHOM2 WT and IRHOM2 C476A mutant, cell medium was incubated with 150 µm Click‐iT palmitic acid, azide and maintained in a 5% CO_2_, 37 °C condition for 6 h. After 6 h treatment, the cells were collected and then washed for 3 times with Hanks' Balanced Salt Solution (HBSS) (Cat#: C0218, Beyotime Biotechnology, China), followed by a mixture with lysis buffer (1% SDS in 50 mMTris‐HCl, pH 8.0)containing 1×protease and phosphatase inhibitor cocktail (EDTA free) (Cat#: HY‐K0013, MedChemExpress, China). The lysates were accordingly maintained in 4 °C for 30 min, then moved the lysis to a 1.5 ml trace centrifugal tube. The final cell lysates were compressed by centrifugation at 4 °C, 15 000 rpm for 5 min. Protein concentration of collected supernatant was confirmed by EZQ Protein Quantitation Kit (Cat#: R33200, Thermo Fisher Scientific) in accordance with product manuals. The total extracted protein samples were then incubated with biotin‐alkyne using Click‐iT Protein Reaction Buffer Kit, as described in procedures of production instruction specification. After that, the corresponding biotin‐alkyne‐palmitic acid, azide‐protein complexes were processed to streptavidin‐mediated pulldown assays via Pierce™ Streptavidin Magnetic Beads (Cat#: 88 817, Thermo Fisher Scientific), followed by western blotting analysis with IRHOM2 antibody.

In addition, to further identify palmitoylation of IRHOM2, a complementary method was also used in this section. The cells also were incubated and lysed by 0.2% SDS, 1% Triton X‐100, 50 mM TEA‐HCl, 150 mM NaCl, pH 7.4 buffer containing 1×protease and phosphatase inhibitor cocktail (EDTA free), accompanied by click reaction with biotin azide. Cell proteins were harvested using 10 volumes of 100% methyl alcohol for 2 h at −80 °C, and then re‐collected by centrifugation at 13 000 rpm for 10 min. The precipitates were accordingly re‐suspended in 100 ml suspension buffer and then diluted to 10‐fold immunoprecipitation buffer containing 150 mM NaCl, 0.5% NP40, 50 mM Tris‐HCl, pH 7.4, and 5 mM EDTA. Labeled cell proteins were enriched by streptavidin agarose at 25 °C with a gentle vortex for 2–3 h. Protein‐bounds streptavidin agarose beads were washed 3 times with immunoprecipitation buffer and protein‐bounds were eluted with elution buffer (95% formamide and 10 mM EDTA, pH 8.2) for 10 min at 95 °C. Samples were subsequently subjected to immunoblotting detection.

### Fractionation Assay

Fractionation of cell proteins was performed using the Subcellular Protein Fractionation Kit (Cat#: NBP2‐47659, Novus Biologicals). The nucleus was lysed with lysis buffer (50 mM TEA, pH 7.3, 150 mM NaCl, 4% SDS, 1×protease inhibitor cocktail (Roche, EDTA free), 1500 units ml^−1^ benzonase nuclease and 2 mM PMSF). EDTA was added to a final concentration of 5 mM after solution removal. The cytoplasmic proteins were diluted with the lysis buffer containing 5 mM EDTA before APE assay.

### Mouse Strains

To obtain practicable mice with a conditional deletion of *Zdhhc3*, the *Zdhhc3^flox/flox^* mice based on C57BL/6 strain were created by Clustered Regularly Interspersed Short Palindromic Repeats‐CRISPR‐associated protein 9 (CRISPR‐Cas9)genetic engineering editing system. The exons 4 & 5 of *Zdhhc3* was targeted as conditional deletion sites to perform conditional knockout. In short, the targeted exons of *Zdhhc3* were flanked by 2 sites of *loxP*, and thus 2 single guide RNAs, guide RNA1 and guide RNA2, targeting *Zdhhc3* introns were demarcated. The packaged plasmids containing 2 *loxP* sites‐flanked *Zdhhc3* exons and the following 2 homology arms were regarded as corresponding template. The targeting vector, guide RNA1 and guide RNA2, and together with Cas9 were coinjected into zygotes for conditional deletion mice establishment. The achieved pups, which had targeted exons flanked by 2 *loxP* sites on 1 allele, were subjected to produce *Zdhhc3^flox/flox^* mice. Hepatocyte‐specific *Zdhhc3* ablation (HepKO) offspring were generated by crossing *Zdhhc3^flox/flox^* offspring with albumin‐cre tool transgenic mice (Alb‐Cre) (Cyagen Biosciences, Guangzhou, China). Moreover, hepatocyte‐specific *Irhom2* deletion mice were produced and used in this work according to our previous study.^[^ ^18^ ^]^

To obtain mice with conditional overexpression of *Zdhhc3*, the Rosa*^Zdhhc3^* mice ground on C57BL/6 strain were constructed by *Zdhhc3* conditional knock‐in at the mouse locus of *Rosaβgeo26* by CRISPR‐Cas9 genetic engineering editing system. Briefly, the designed cassette of *Rosaβgeo26*‐(pCAG)‐*loxp*‐STOP‐*loxp*‐m*Zdhhc3*‐pA box was inserted into 1^st^ intronof *Rosaβgeo26*. Subsequently, the targeting vector, guide RNA, and Cas9 were co‐injected into eggs for Rosa*^Zdhhc3^* mouse establishment. In the indicated experiments, the conditional overexpression of *Zdhhc3* in hepatocytes (HepTG) were triggered by injection of adeno‐associated virus serotype‐8 (AAV8)‐thyroxine‐binding globulinpromoter (TBG)‐recombinase Cre vector (AAV8‐TBG‐Cre) via intravenous injection and then determined by immunoblotting assay. Rosa*^Zdhhc3^* littermates with AAV‐blank injection were used as corresponding controls for the procured HepTG mice.

Additionally, the hepatocyte‐specific *Zdhhc3*&*Irhom2* double‐deficiency (HepDKO) mice were produced by mating *Zdhhc3^flox/flox^* mice with *Irhom2*‐HepKO mice. The obtained offspring without Zdhhc3 and Irhom2 protein expression were identified and selected by western blotting assay and used for further indicated experiments. Besides, wild‐type C57BL/6N strain mice (WT, male, 6‐8‐week‐old) used in this work were purchased from Beijing Vital River Laboratory Animal Technology Co., Ltd., (Beijing, China).

### Construction of Nonalcoholic Steatohepatitis (NASH) Animal Model

Two different mouse NASH models and one rabbit NASH model were constructed and used in the current study for the corresponding experiments.

### High Fat+High Cholesterol Diet (HFHC)‐Induced NASH Model

A rodent model with NASH phenotype was generated by feeding the mice a HFHC fodder (HFHC) (42% saturated‐fat, 14% protein, 44% carbohydrates, and 0.2% cholesterol w/w) for 16 weeks.^[^ ^17^, ^18^ ^]^ The experimental mice were treated with a standard normal chow diet (NCD) (Cat: D12450J; Research Diets, USA) for consecutive 16 weeks to be regarded as corresponding controls for the procured HFHC model. For the rabbit in vivo experiment, according to a previous report with certain modification,^[^ ^40^, ^41^, ^42^ ^]^ male New Zealand white rabbits were treated with corresponding HFHC diet (standard diet with an additional 2% maltodextrin, 2% cholesterol, and 10% saturated fats, Cat: 621 079; Dyets, Bethlehem, Pa) for 8 weeks to establish rabbit NASH model.

### Western‐Type Diet+15% Fructose (w/v)‐Drinking Water (WTDF)‐Induced NASH Model

The second mice model with NASH phenotype was obtained by feeding the corresponding mice a WTDF diet (Cat: D12079B; Research Diets, Inc., New Brunswick, USA) supplemented with 15% w/v fructose (Cat: 630 030 391, Sinopharm Chemical Reagent Co., Ltd., China)‐drinking water (Cat: 1 010 004 000, C'estbon, China) for 16 weeks.^[^ ^17^, ^18^ ^]^ Control mice received a normal chow diet.

### Animals Treatment and Model Design

To minimize the effects of hormones oscillationon metabolism, only male animals (6–8 weeks old) were used for all experiments in this work. Before the experiment starts, animals participating in the corresponding experiment were forced to accommodate to their living environment for 7 days. The animals were kept at a steady temperature, moisture capacity (governed by Haier central air conditioning, Cat: RFC140MXSCVD/G, China), and aseptic conditions‐controlled environment (25 °C, 55%−60%) cage with a constant and standard 12/24 h‐12/24 h light/dark circle, unlimited pathogen‐free‐drinking water (Cat: 1 010 004 000, C'estbon, China) and fodder in their houses.

**Animal study design 1#**: To estimate the effects of Zdhhc3 on HFHC diet‐triggered NASH pathologies, hepatocyte‐specific *Zdhhc3* deletion (HepKO) mice were treated with HFHC or WTDF diet for 16 weeks to induce NASH phenotype, respectively. To obtain the conditional *Zdhhc3* gain‐of‐function mice, the HFHC diet‐fed Rosa*^Zdhhc3^* mice were injected with a 1.5 × 10^12^ genome copies (gc) dose of an AAV8‐TBG‐Cre vectors (#SL101528, SignaGen Laboratories) via the tail vein to induce hepatocytes‐specific *Zdhhc3* overexpression (HepTG HFHC). Rosa*^Zdhhc3^* mice given an equal dose of AAV empty vector were treated as controls (HepNTG HFHC).

**Animal study design 2#**: To investigate the function of Zdhhc3 in NASH progression, full‐length of mouse Zdhhc3 sequences or mouse Zdhhc3 sequences with C157A in DHHC domain mutant were loaded in AAV8 vector to create AAV‐TBG‐*Zdhhc3* or AAV‐TBG‐*Zdhhc3* (C157A) vectors. The *Zdhhc3*‐HepKO mice were then injected with a 1.5 × 10^12^ genome copies (gc) dose of corresponding vectors to produce Zdhhc3 gain‐of‐function mice. These mice were further fed with a 16‐week HFHC diet to induce NASH phenotype. The corresponding mice were injected with blank vectors were used as controls.

**Animal study design 3#**: The male *Irhom2*/*Zdhhc3*‐HepKO mice, *Irhom2*‐HepKO mice, *Zdhhc3*‐HepKO mice, and *Zdhhc3*‐Flox mice were treated with 16‐weeks HFHC diet to produce NASH phenotype and estimate the physiopathological changes.

**Animal study design 4#**: For the rabbit in vivo experiment, according to the previous report with certain modification,^[^ ^40^, ^41^, ^42^ ^]^ Male 20 New Zealand white rabbits (1.75–2.00 kg BW) were treated with corresponding HFHC diet (standard diet with an additional 2% maltodextrin, 2% cholesterol, and 10% saturated fats, Cat: 621 079; Dyets, Bethlehem, Pa) for 8 weeks to establish rabbit NASH model. Pharmacological function evaluation of HFHC‐fed rabbits and WT mice were performed accordingly. Rabbits with NASH phenotype were treated with 40 mg kg^−1^ 2‐BP, *i.p*., every 2 days for 8 weeks. Male WT mice with NASH phenotype were injected with 0, 2.5, 10, and 20 mg kg^−1^ 2‐BP, *i.h*., every 2 days for 16 weeks. After the experiment, the liver tissue was harvested from indicated animals model to examine corresponding signal indicators.

### Statistical Analysis

The associated results showed in this work were processed independently at least 3 times. All raw data involving this work were independently analyzed by suitable statistic protocols, as assigned in the figures legends. Unless otherwise stated, quantitative scores of data are presented as mean ± SEM. ANOVA was used for the comparison approach, multiple groups were performed using *Dunnett's* multiple comparison tests, and two groups comparison was performed using 2 tailed Student's *t*‐test. GraphPad Prism Software (Version 9.4.1.681 for Microsoft Windows; GraphPad Software, San Diego, USA), the R packages, and IBM SPSS Statistics (Version 25.0, Microsoft Windows, IBM, USA) were performed for the final data presentation. *P* < 0.05 means statistically significant difference.

## Conflict of Interest

The authors declare no conflict of interest.

## Author Contributions

M.X., J.T., L.Z., C.G., Y.Z., and F.G. contributed equally to this work. M.X., J.T., C.G., and B.W. performed the conceptualization and methodology; M.X., J. T., C.G., X.D., Q.K., Y.Z., L.Z., and F.G. performed investigations; M.X., J.T., L.Z., and C.G. performed data analysis; M.X., J.T., B.W., J.C., B.Z. acquired funding, project administration, and supervised the study; : M.X., J.T., C.G., B.W., and J.C. wrote the original draft; M.X., B.W., J.T., L.Z., and B.Z. reviewed and edited the final manuscript.

## Supporting information

Supporting Information

Supporting Information

## Data Availability

The data that support the findings of this study are available from the corresponding author upon reasonable request.
