# Supplementary material for: Palmitoyltransferase ZDHHC3 Aggravates Nonalcoholic Steatohepatitis by Targeting S‐Palmitoylated IRHOM2
Source: Adv Sci (Weinh). 2023 Aug 6;10(28):2302130. doi: 10.1002/advs.202302130 (PMC10558657; doi:10.1002/advs.202302130)
Supplement: Supplementary file 1 — Supporting Information [file ADVS-10-2302130-s002.pdf]

ADVANCED SCIENCE
Open Access

Supporting Information

for *Adv. Sci.*, DOI 10.1002/advs.202302130

Palmitoyltransferase ZDHHC3 Aggravates Nonalcoholic Steatohepatitis by Targeting *S*-Palmitoylated IRHOM2

Minxuan Xu, Jun Tan*, Liancai Zhu, Chenxu Ge, Yi Zhang, Fufeng Gao, Xianling Dai, Qin Kuang, Jie Chai, Benkui Zou* and Bochu Wang**

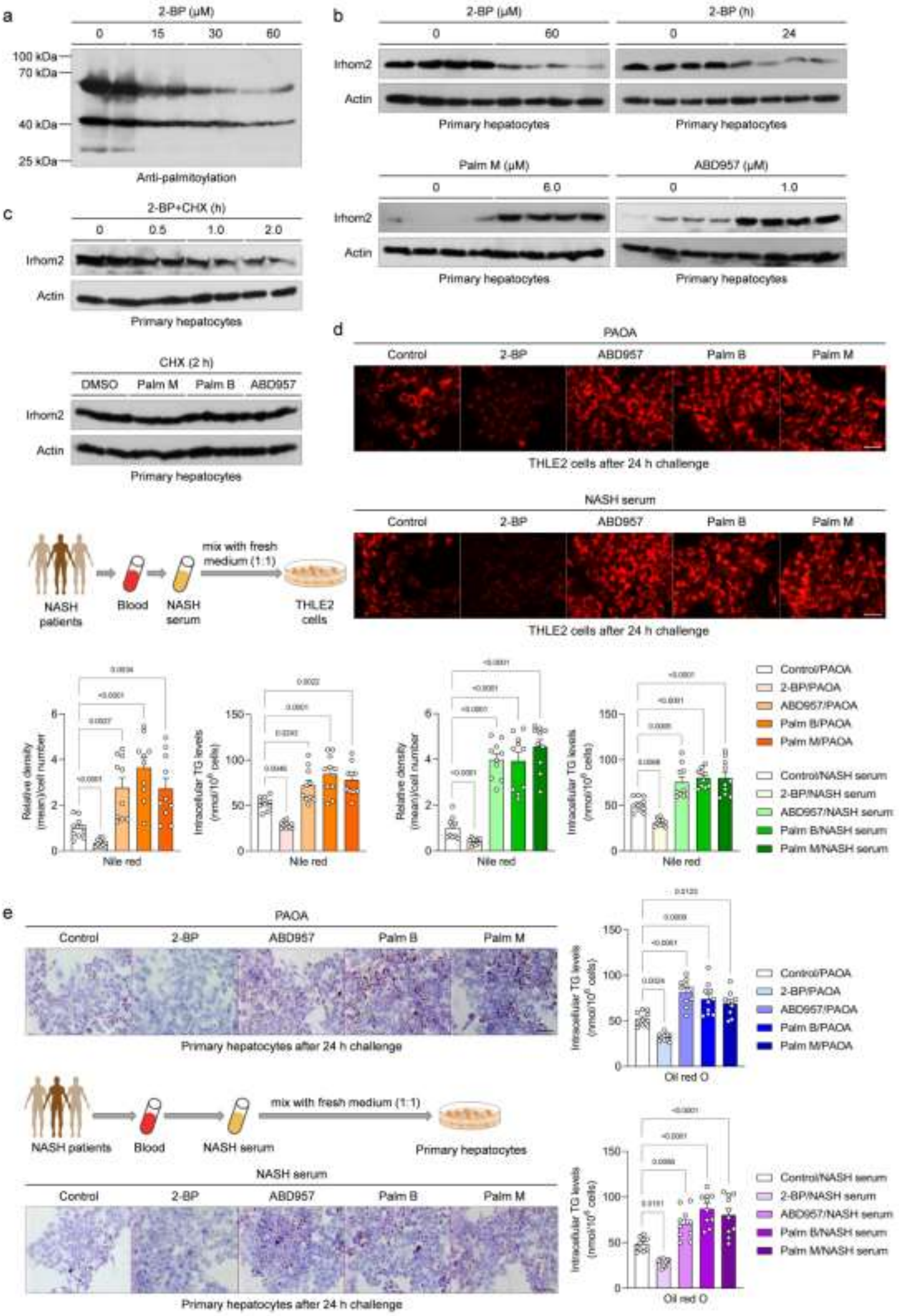

Supplementary figure 1. Palmitoylated IRHOM2 is positively correlated with lipid accumulation.

a, Human hepatocyte THLE2 cells were incubated with concentration-gradient of 2-bromopalmitate (2-BP) for 24 h, and subjected to immunoblotting detection with palmitoylation antibody, antibody, suggesting that 2-BP decreased proteins palmitoylation abundance in the whole cell lysates. (*n*=4 per group). **b**, Mouse primary hepatocytes were treated with 60 μM 2-BP, 1 μM ABD957 and 6 μM Palm B for 24 h, respectively. The lysates were then subjected to immunoblotting assay with Irhom2 and Actin antibodies (*n*=4 per group). **c**, Upper, mouse

primary hepatocytes were pre-incubated with 2-BP as a baseline, then treated with cycloheximide (CHX) in the time-course frame. Lower, mouse primary hepatocytes were pre-treated with DMSO, 1 μM ABD957, 6 μM Palm B and 10 μM Palm M for 24 h, followed by treatment with cycloheximide (CHX) for 2 h. The collected cell lysates were subjected to immunoblotting detection with Irhom2 and Actin antibodies (*n*=4 per group). **d**, Human hepatocyte THLE2 cells were co-treated with 60 μM 2-BP, 1 μM ABD957, 6 μM Palm B or 10 μM Palm M and 0.5 mM palmitic acid+1.0 mM oleic acid (PAOA) mixture (upper) or NASH serum (lower) for 24 h, as indicated the experimental design outline. The collected fixed sections were subjected to Nile red staining analysis to observe lipid accumulation *in vitro* (*n*=10 images per group; $P < 0.05$ vs. Control/PAOA or Control/NASH serum group). Scale bar, 100 μm. **e**, Mouse primary hepatocytes were co-treated with 60 μM 2-BP, 1 μM ABD957, 6 μM Palm B or 10 μM Palm M and PAOA (upper) or NASH serum (lower) for 24 h, as indicated the experimental design outline. The collected fixed sections were subjected to Oil red O staining analysis to detect lipid accumulation *in vitro* (magnification, 100×. *n*=10 images per group; $P < 0.05$ vs. Control/PAOA or Control/NASH serum group). Data are expressed as mean ± SEM. The relevant experiments presented in this part were performed independently at least three times. Significance determined by one-way analysis of variance (ANOVA) followed by Dunnett's multiple comparisons test analysis (**d**, **e**). The *P* value less than 0.05 was considered as significant difference.

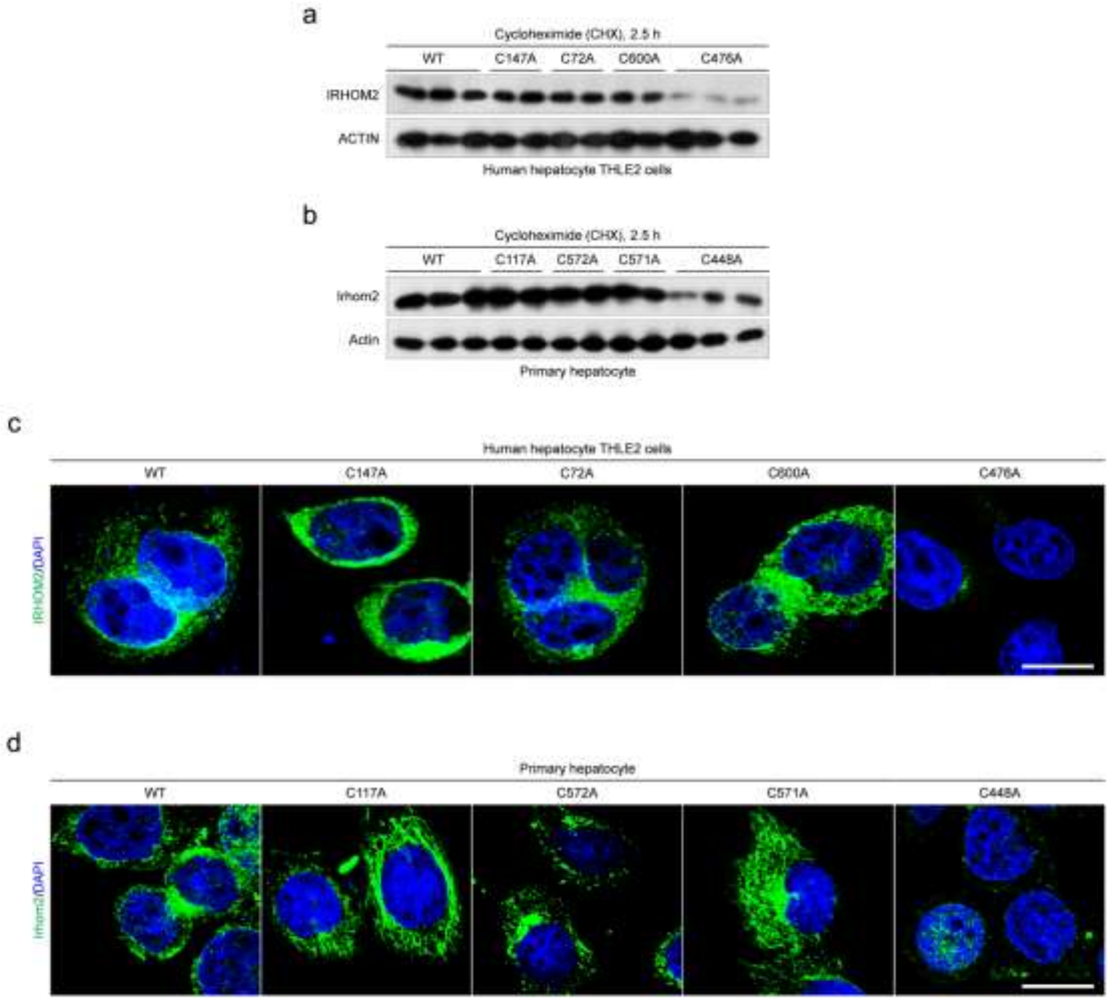

Supplementary figure 2. Identification of cysteine 476 for *Homo sapiens* and cysteine 448 for *Mus musculus* as the palmitoylation site on IRHOM2.

a, The degradation of IRHOM2 WT, IRHOM2 C147A mutant, IRHOM2 C72A mutant, IRHOM2 C600A mutant and IRHOM2 C476A mutant in human hepatocyte THLE2 cells were detected after 2.5 h CHX treatment (*n*=3 samples per group). **b**, The degradation of Irhom2 WT, Irhom2 C117A mutant, Irhom2 C572A mutant, Irhom2 C571A mutant and Irhom2 C448A mutant in mouse primary hepatocytes were detected after 2.5 h CHX treatment (*n*=3 samples per group). **c**, **d**, THLE2 cells (**c**) or mouse primary hepatocytes (**d**) transfected with indicated mutant vectors were then subjected to immunofluorescence analysis with to detect IRHOM2 protein abundance. The fixed sections were subjected to immunofluorescent staining with IRHOM2 (green) and DAPI (blue) (*n*=10 images per group). Scale bars, 10 μm.

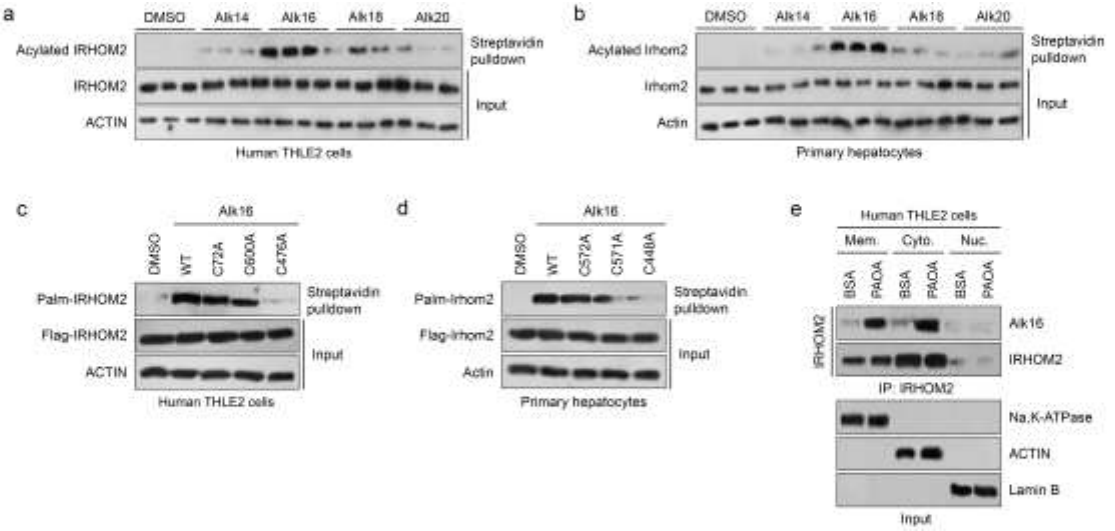

Supplementary figure 3. IRHOM2 is *S*-palmitoylated at evolutionarily conserved cysteine residues over the course of fatty acid challenge.

a, Detection of IRHOM2 *S*-palmitoylation in human hepatocyte THLE2 cells using different alkyl-labeled fatty acylation including Alk14, Alk16, Alk18 and Alk20. Acylated IRHOM2 were detected by streptavidin bead pulldown, followed by immunoblotting assay with IRHOM2 and ACTIN antibodies (*n*=6 samples per group). **b**, The same protocol similar with (**a**) was used to detect acylated IRHOM2 in primary hepatocytes. The lysates were then subjected to western blotting analysis with Irhom2 and Actin antibodies (*n*=6 samples per group). **c**, **d**, Palmitoylation abundance of Flag-labeled IRHOM2 WT, IRHOM2 C72A, IRHOM2 C600A and IRHOM2 C476A mutants (**c**) for human, and Irhom2 WT, Irhom2 C572A, Irhom2 C571A and Irhom2 C448A mutants for mouse (**d**) were analyzed by labelling with Alk16 via CLICK reaction -associated streptavidin pulldown. The corresponding lysates were subjected to immunoblotting assay with Flag and ACTIN antibodies (*n*=3 samples per group). **e**, In response to PAOA challenge, the wild-type THLE2 cells were transfected with IRHOM2-Flag, followed by labelling with Alk16. Subcellular fraction was collected and IRHOM2 protein levels were modulated to confirm that there were equal amount of IRHOM2 in the indicated cell component for input. Palmitoylated IRHOM2 levels in cell membrane (Mem.), cell cytoplasm (Cyto.) and cell nucleus (Nuc.) component were observed by immunoblotting analysis. Data are expressed as mean ± SEM. The relevant experiments presented in this part were performed independently at least three times.

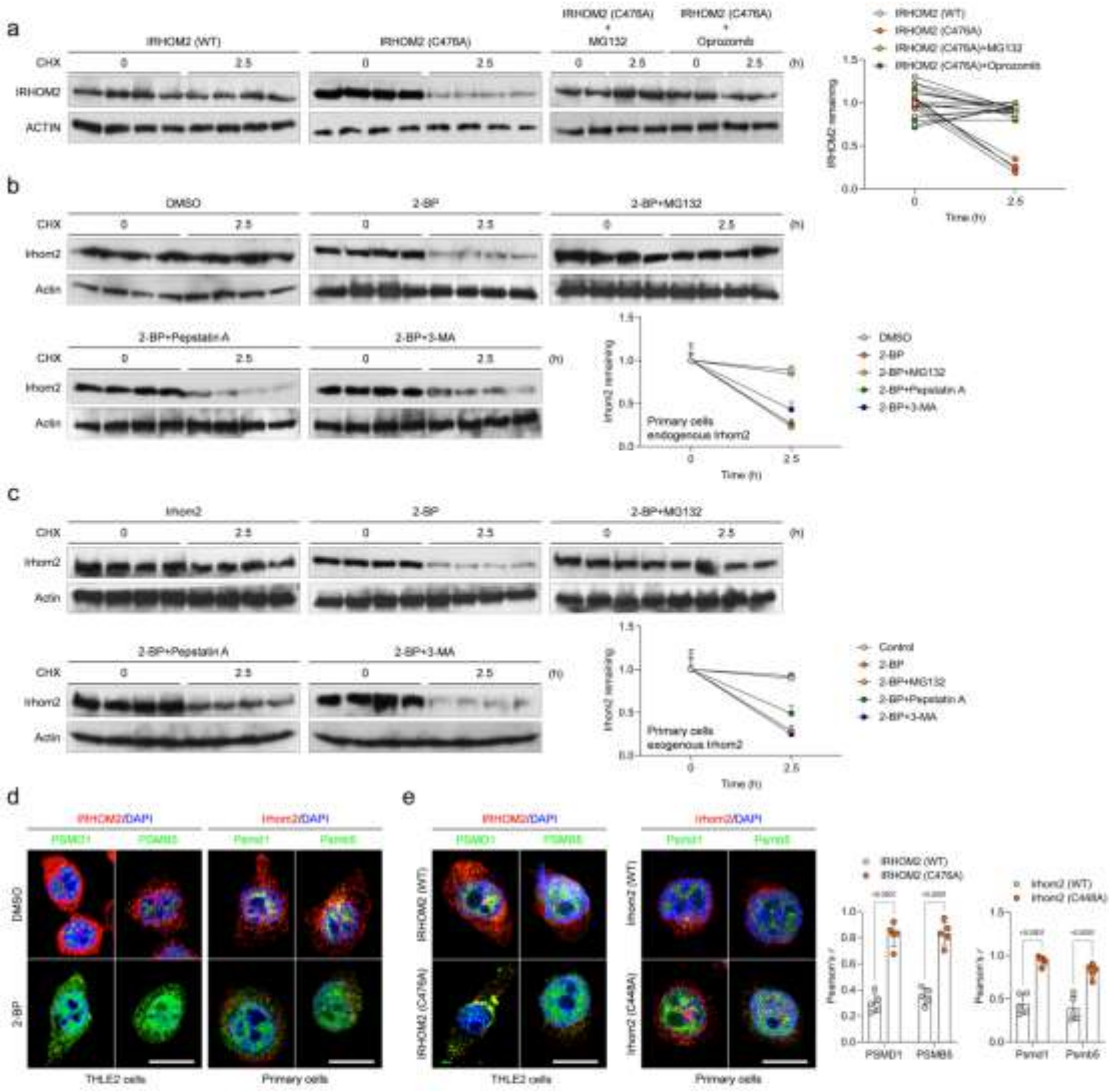

Supplementary figure 4. Palmitoylation maintains stabilization of IRHOM2 by blocking its proteasome degradation.

a, The THLE2 cells were transfected with IRHOM2 or IRHOM2 C476A mutant overexpression, followed by detection of IRHOM2 degradation under 2.5 h CHX treatment, in the presence or absence of proteasome inhibitor (MG132 and oprozomib). The right graph showing the relative IRHOM2 remaining ratio in the indicated time point (*n*=4 per group). **b**, The 2.5 h CHX treatment detection showing the effects of 2-BP on Irhom2 degradation in primary hepatocytes with/without different inhibitors (MG132 for proteasome, Pepstatin A for lysosome and 3-MA for autophagy). The right graph showing the relative Irhom2 remaining level in the indicated time point (*n*=4 per group). **c**, The 2.5 h CHX treatment detection showing the effects of different inhibitors (MG132 for proteasome, Pepstatin A for lysosome and 3-MA for autophagy) on the degradation of Irhom2 in primary hepatocytes. The right graph showing the relative Irhom2 remaining level in the indicated time point (*n*=4 per group). **d**, Representative immunofluorescence images showing the assembled proteasome markers, PSMD1 and PSMB5 expression levels (green), and IRHOM2 (red) coexpression in THLE2 cells and mouse primary hepatocytes in response to 2-BP treatment (*n*=5 images per group). Scale bars, 10 μm. **e**, Representative immunofluorescence images showing PSMD1 and PSMB5 expression levels (green), and IRHOM2 (red) coexpression in THLE2 cells and mouse primary hepatocytes with IRHOM2 WT and IRHOM2 C476A expression. Scale bars, 10 μm. The right bar graph showing the relative IRHOM2 levels in the indicated groups (*n*=5 images per group; *P* < 0.05 vs. WT group). Data are expressed as mean ± SEM. The relevant experiments presented in this part were performed independently at least three times. Significance determined by 2-sided Student's *t*-test analysis (**e**). The *P* value less than 0.05 was considered as significant difference.

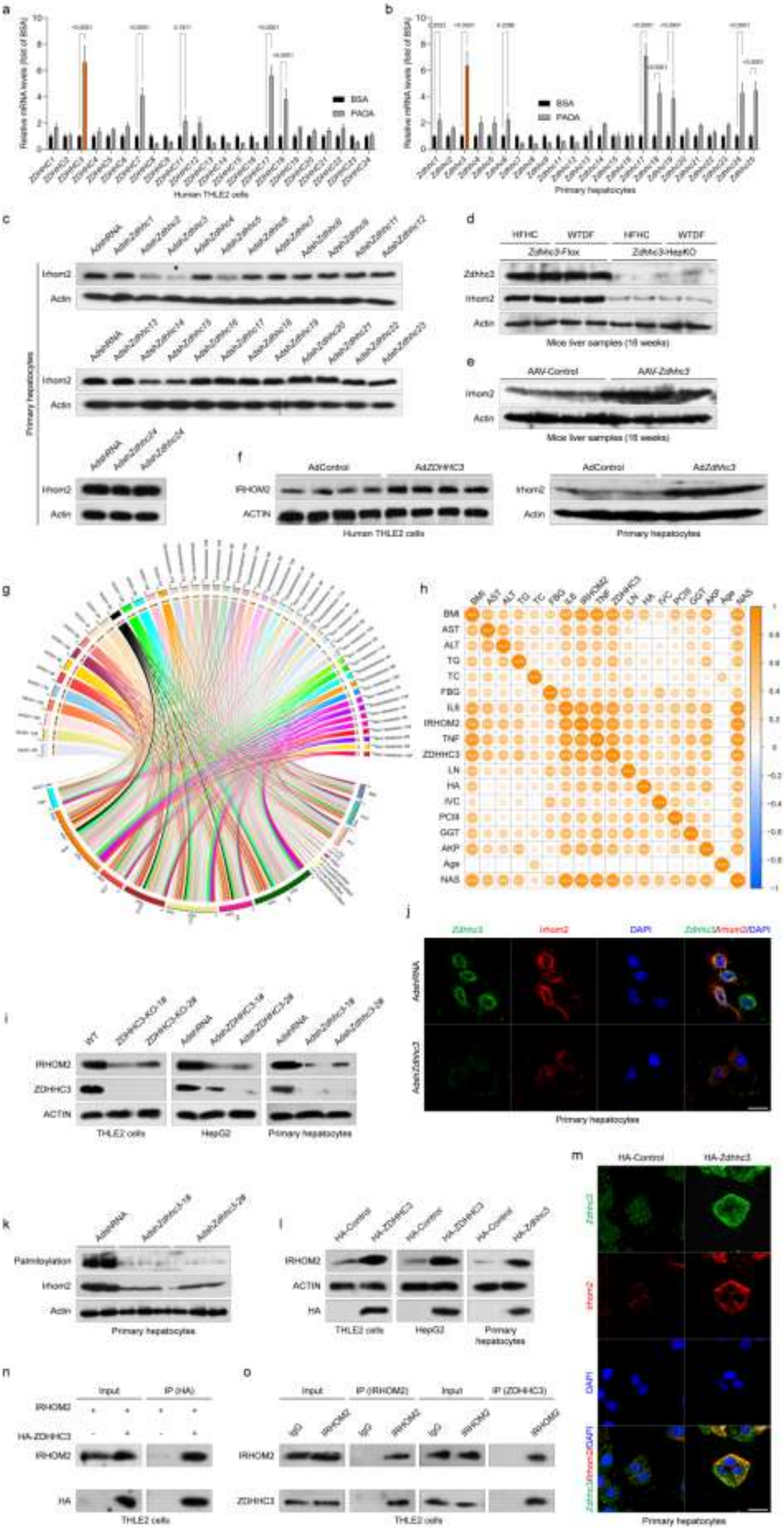
a
b
BSA
PADA
Human THLE2 cells
Primary hepatocytes
c
Primary hepatocytes
Irhom2
Actin
d
HFHC
WTDF
Zdhhc3-Flox
Zdhhc3-HepKO
Zdhhc3
Mice liver samples (16 weeks)
e
AAV-Control
AAV-Zdhhc3
f
AdControl
AdZDHHC3
IRHOM2
ACTIN
Human THLE2 cells
AdZdhhc3
g
h
BMI
AST
ALT
TG
TC
FBG
IL6
IRHOM2
TNF
ZDHHC3
LN
HA
IVC
PCIII
GGT
AKP
Age
NAS
i
THLE2 cells
HepG2
j
Zdhhc3
DAPI
k
Palmitoylation
l
HA-Control
HA-ZDHHC3
HA
m
HA-Zdhhc3
n
Input
IP (HA)
HA-ZDHHC3
THLE2 cells
o
IP (IRHOM2)
IP (ZDHHC3)
IgG
ZDHHC3

Supplementary figure 5. Identification of ZDHHC3 as a key palmitoyltransferase for IRHOM2 palmitoylation.

a, **b**, qPCR analysis showing the ZDHHCs mRNA expression levels in human THLE2 cells (**a**) and mouse primary hepatocytes (**b**) upon PAOA challenge. (*n*=4 per group; P < 0.05 vs. BSA group). **c**, Representative western blotting bands showing the Irhom2 expression changes in mouse primary hepatocytes with different adenovirus-mediated Zdhhcs (Ad*Zdhhc*s) transfection. The asterisk indicates the group with the most change among all the candidates (*n*=4 per group). **d**, Representative western blotting bands showing the Zdhhc3 and Irhom2 expression levels of mice liver samples in the indicated groups (*n*=4 per group). **e**, Representative western blotting bands showing the Irhom2 expression levels in AAV-*Zdhhc3*-mediated Zdhhc3 overexpressed mice after 16-weeks HFHC diet administration (*n*=4 per group). **f**, Human THLE2 cells or mouse primary hepatocytes transfected with Ad*ZDHHC3* or Ad*Zdhhc3* were then subjected to western blotting assay with IRHOM2 and ACTIN antibodies (*n*=4 per group). **g**, Circle correlation indicating the correlations between a series of indicators correlation in human subjects. P < 0.001 for all of these correlations (*n*=10 samples for non-steatosis phenotype; *n*=17 samples for simple steatosis phenotype; *n*=16 samples for NASH phenotype). **h**, Multiple pearson correlation analysis for human subjects exhibiting the comprehensive correlation between IRHOM2 and ZDHHC3 expression, and indicated parameter indexes (*n*=49 indices per parameter). **i**, Representative western blotting bands indicating IRHOM2 and ZDHHC3 expression changes in the indicated groups (*n*=4 per group). **j**, Representative immunofluorescence images showing Irhom2 and Zdhhc3 coexpression in Adsh*Zdhhc3*-transfected mouse primary hepatocytes (*n*=5 images per group). Scale bar, 50 μm. **k**, After Adsh*Zdhhc3*-transfection, mouse primary hepatocytes lysates were subjected to western blotting assay to show palmitoylation and Irhom2 abundance (*n*=4 per group). **l**, Representative western blotting bands indicating the interaction of IRHOM2 and ZDHHC3 in indicated groups. **m**, Representative immunofluorescence images showing Zdhhc3 and Irhom2 coexpression in mouse primary hepatocytes with HA-Zdhhc3 transfection (*n*=5 images per group). Scale bar, 50 μm. **n**, THLE2 cells were co-transfected with IRHOM2 and HA-ZDHHC3 and subjected to IP of HA. **o**, Mutual Co-IP of IRHOM2 and ZDHHC3 indicating binding between endogenous IRHOM2 and ZDHHC3 in THLE2 cells. Data are expressed as mean ± SEM. The relevant experiments presented in this part were performed independently at least three times. Significance determined by 2-sided Student's *t*-test analysis (**a**, **b**). The P value less than 0.05 was considered as significant difference.

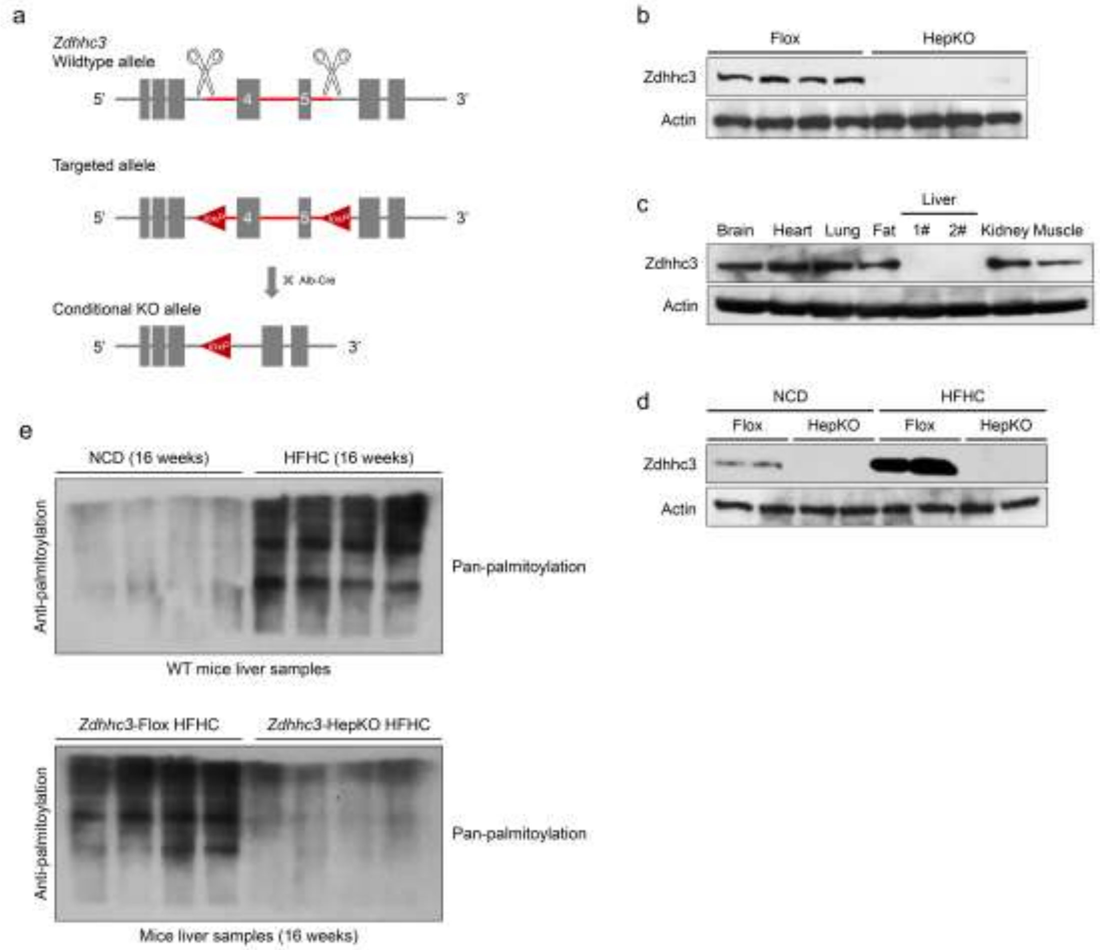

Supplementary figure 6. Schematic diagram for establishment of hepatocyte-specific *Zdhhc3* knockout in mice.

a, Experimental design for hepatocyte-specific *Zdhhc3* knockout in rodent model. **b**, **c**, Characterization and representative immunoblotting bands of Zdhhc3 expression levels in the liver samples of Flox mice and HepKO mice (**b**), and in different tissue (**c**) of HepKO mice (*n*=4 mice per group). **d**, Representative immunoblotting bands of Zdhhc3 protein levels in liver samples of HFHC-fed Flox mice and HepKO mice (*n*=4 mice per group). **e**, Representative western blotting analysis showing the pan-palmitoylation levels in liver samples of the 16 weeks NCD-fed WT mice and HFHC-fed WT mice (upper), and in liver tissue collected from the 16 weeks HFHC-challenged Flox mice and *Zdhhc3*-HepKO mice (lower) (*n*=4 mice per group). Data are expressed as mean ± SEM. The relevant experiments presented in this part were performed independently at least three times. *P* <0.05 indicates statistical significance.

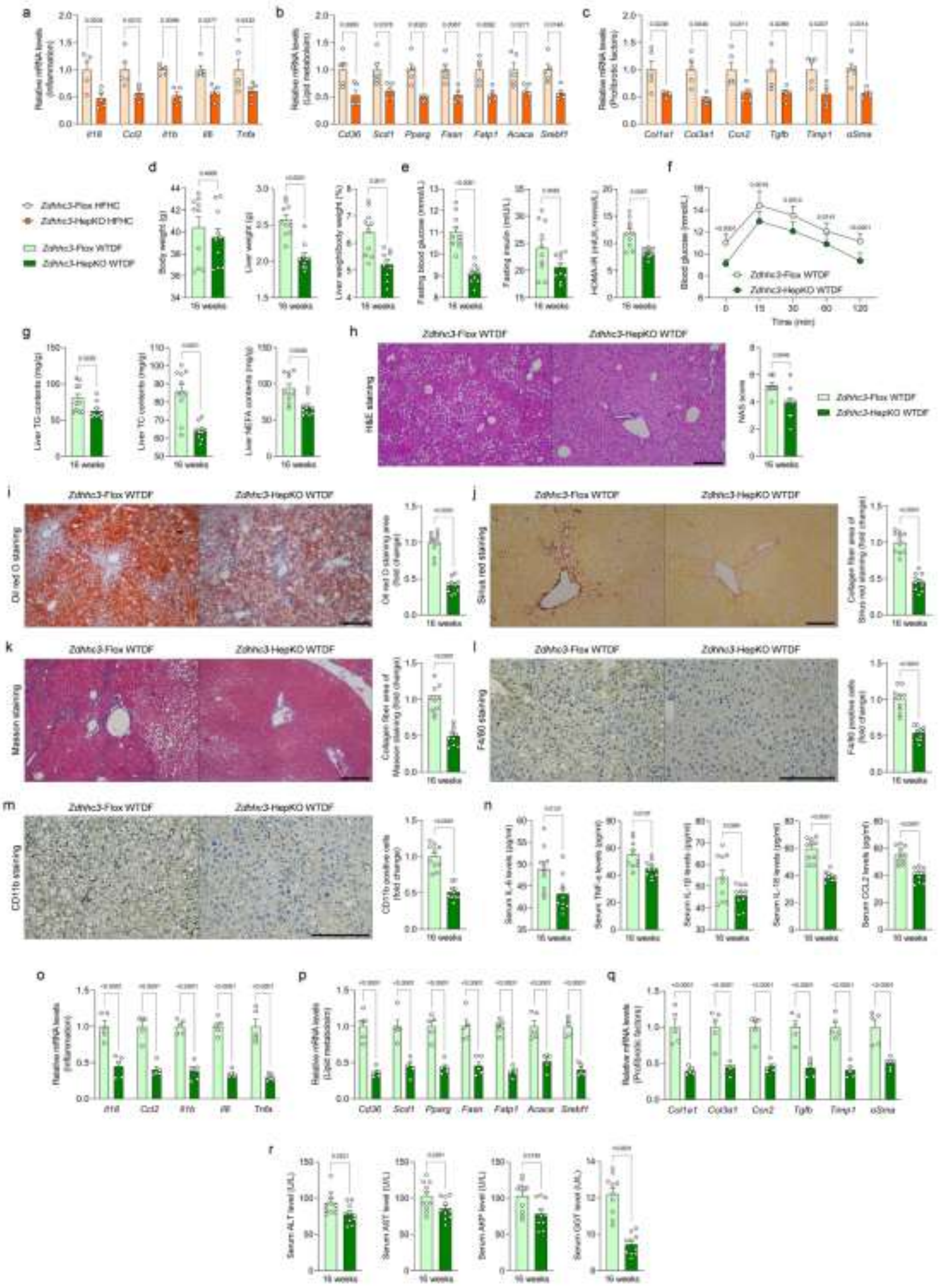

Supplementary figure 7. Hepatocyte-specific *Zdhhc3* deficiency retards WTDF-induced NASH pathogenesis.

a-c, Representative mRNA levels of pro-inflammation (**a**)-, fatty acid metabolism (**b**)- and profibrotic factors (**c**)-associated genes expression in livers from HFHC-fed HepKO or Flox mice (n=5 mice per group; P < 0.05 vs. *Zdhhc3*-Flox HFHC group). **d-g**, Records for the body weight, liver weight and the ratio of liver weight/body weight (%) (**d**), fasting blood glucose levels, fasting insulin levels and HOMA-IR index (**e**), glucose tolerance test (GTT) (**f**) and liver lipid contents including TG, NEFA and TC (**g**) in WTDF-fed HepKO or Flox mice (n=10 mice per group; P < 0.05 vs. *Zdhhc3*-Flox WTDF group). **h-m**, Representative pictures of H&E staining and histological NAS score changes (**h**), oil red O staining (**i**), sirius red staining (**j**), masson staining

(**k**) and F4/80 and CD11b positive cells expression (**l**, **m**) in indicated groups (magnification, 100× for H&E staining, oil red O staining, masson staining and sirius red staining; magnification, 200× for F4/80 and CD11b staining; *n*=10 images per group; $P < 0.05$ vs. *Zdhhc3*-Flox WTDF group). **n**, Records for inflammation-related cytokines profiles including IL-6, TNF-α, IL-1β, IL-18 and CCL2 in serum from WTDF-fed HepKO or Flox mice (*n*=10 mice per group; $P < 0.05$ vs. *Zdhhc3*-Flox WTDF group). **o-q**, Representative mRNA levels of pro-inflammation (**o**)-, fatty acid metabolism (**p**)- and profibrotic factors (**q**)-associated genes expression in livers from WTDF-fed HepKO or Flox mice (*n*=5 mice per group; $P < 0.05$ vs. *Zdhhc3*-Flox WTDF group). **r**, Records for liver function-related indicators including ALT, AST, AKP and GGT in serum from WTDF-fed HepKO or Flox mice (*n*=10 mice per group; $P < 0.05$ vs. *Zdhhc3*-Flox WTDF group). Data are expressed as mean ± SEM. The relevant experiments presented in this part were performed independently at least three times. Significance determined by 2-sided Student's *t*-test. The *P* value less than 0.05 was considered as significant difference.

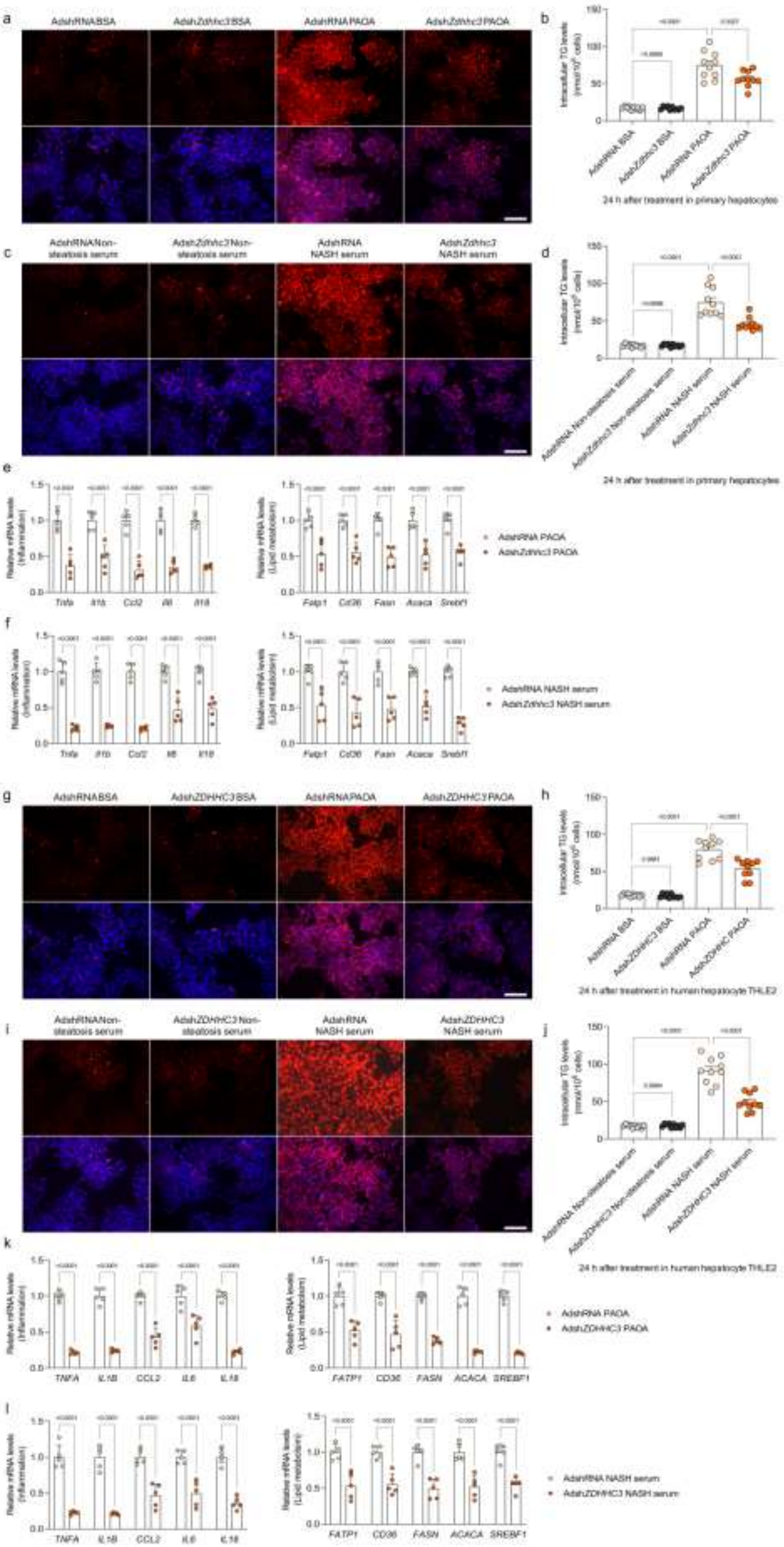

a
AdshRNA BSA
AdshZdhhc3 BSA
AdshRNA PAOA
AdshZdhhc3 PAOA
b
Intracellular TG levels (nmol/10^6 cells)
24 h after treatment in primary hepatocytes
c
AdshRNA Non-steatosis serum
AdshZdhhc3 Non-steatosis serum
AdshRNA NASH serum
AdshZdhhc3 NASH serum
d
24 h after treatment in primary hepatocytes
e
Relative mRNA levels (Inflammation)
Relative mRNA levels (Lipid metabolism)
Tnfa
Il1b
Ccl2
Il6
Il18
Fatp1
Cd36
Fasn
Acaca
Srebf1
AdshRNA PAOA
AdshZdhhc3 PAOA
f
AdshRNA NASH serum
AdshZdhhc3 NASH serum
g
AdshRNA BSA
AdshZDHHC3 BSA
AdshRNA PAOA
AdshZDHHC3 PAOA
h
24 h after treatment in human hepatocyte THLE2
i
AdshRNA Non-steatosis serum
AdshZDHHC3 Non-steatosis serum
AdshRNA NASH serum
AdshZDHHC3 NASH serum
j
24 h after treatment in human hepatocyte THLE2
k
TNFA
IL1B
CCL2
IL6
IL18
FATP1
CD36
FASN
ACACA
SREBF1
AdshRNA PAOA
AdshZDHHC3 PAOA
l
AdshRNA NASH serum
AdshZDHHC3 NASH serum

Supplementary figure 8. Downregulated ZDHHC3 suppresses lipid deposition, and inflammation *in vitro* upon challenge with metabolic insults.

a, **b**, Representative images (**a**) and intracellular triglyceride (TG) (**b**) analysis of the Nile red staining of primary hepatocytes that were transfected with Adsh*Zdhhc3* and co-treated with PAOA for 24 hours (*n*=10 images per group; P < 0.05 vs. AdshRNA group). Scale bar, 200 μm. **c**, **d**, Representative images (**c**) and intracellular triglyceride (TG) (**d**) analysis of the Nile red staining of primary hepatocytes that were transfected with Adsh*Zdhhc3* and co-treated with NASH serum for 24 hours (*n*=10 images per group; P < 0.05 vs. AdshRNA group). Scale bar, 200 μm. **e**, **f**, qPCR analysis showing the inflammation (**e**)- and lipid metabolism (**f**)-related key genes expression changes in indicated groups (*n*=5 per group; P < 0.05 vs. AdshRNA group). **g**, **h**, Representative images (**g**) and intracellular triglyceride (TG) (**h**) analysis of the Nile red staining of THLE2 cells that were transfected with Adsh*ZDHHC3* and co-treated with PAOA for 24 hours (*n*=10 images per group; P < 0.05 vs. AdshRNA group). Scale bar, 200 μm. **i**, **j**, Representative images (**i**) and intracellular triglyceride (TG) (**j**) analysis of the Nile red staining of THLE2 cells that were transfected with Adsh*ZDHHC3* and co-treated with NASH serum for 24 hours (*n*=10 images per group; P < 0.05 vs. AdshRNA BSA group). Scale bar, 200 μm. **k**, **l**, qPCR analysis showing the inflammation (**k**)- and lipid metabolism (**l**)-related key genes expression changes in indicated groups (*n*=5 per group; P < 0.05 vs. AdshRNA group). Data are expressed as mean ± SEM. The relevant experiments presented in this part were performed independently at least three times. Significance determined by one-way analysis of variance (ANOVA) followed by Dunnett's multiple comparisons test analysis (**b**, **d**, **h**, **j**) or 2-sided Student's *t*-test (**e**, **f**, **k**, **l**). The P value less than 0.05 was considered as significant difference.

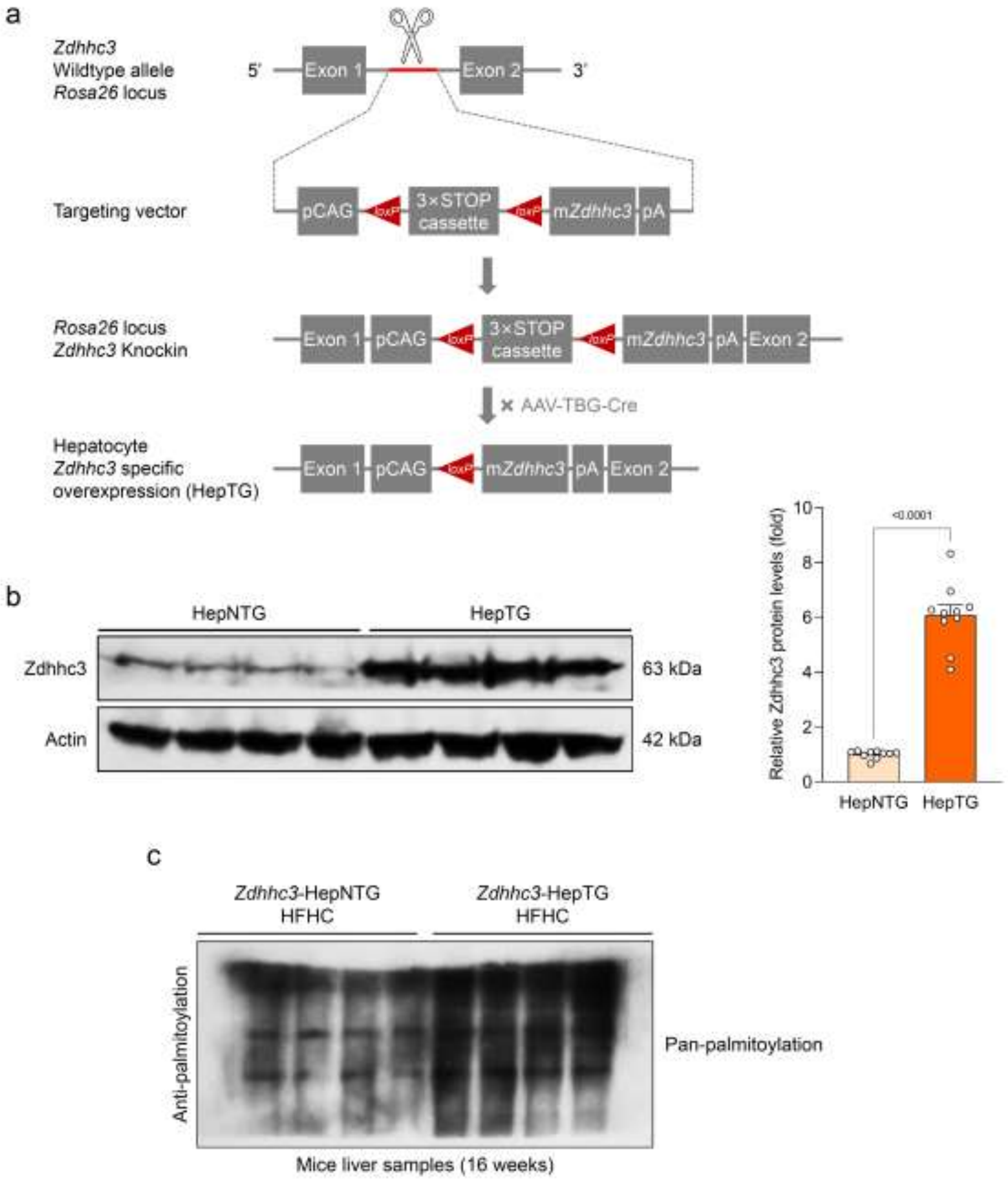

Supplementary figure 9. Establishment and experimental outline of hepatocyte-specific *Zdhhc3* overexpression mice.

a, Schematic diagram of the establishment of hepatocyte-specific *Zdhhc3* overexpression (HepTG) mouse strain. **b**, Characterization and representative western blotting bands of *Zdhhc3* expression in the liver samples of control mice (HepNTG) and HepTG mice (n=8 mice per group; $P < 0.05$ vs. HepNTG group). **c**, Representative western blotting analysis showing the pan-palmitoylation levels in liver samples collected from the 16 weeks HFHC-challenged *Zdhhc3*-HepNTG mice and *Zdhhc3*-HepTG mice (n=4 mice per group). Data are expressed as mean ± SEM. The relevant experiments presented in this part were performed independently at least three times. Significance determined by 2-sided Student's t-test (**b**). The P value less than 0.05 was considered as significant difference.

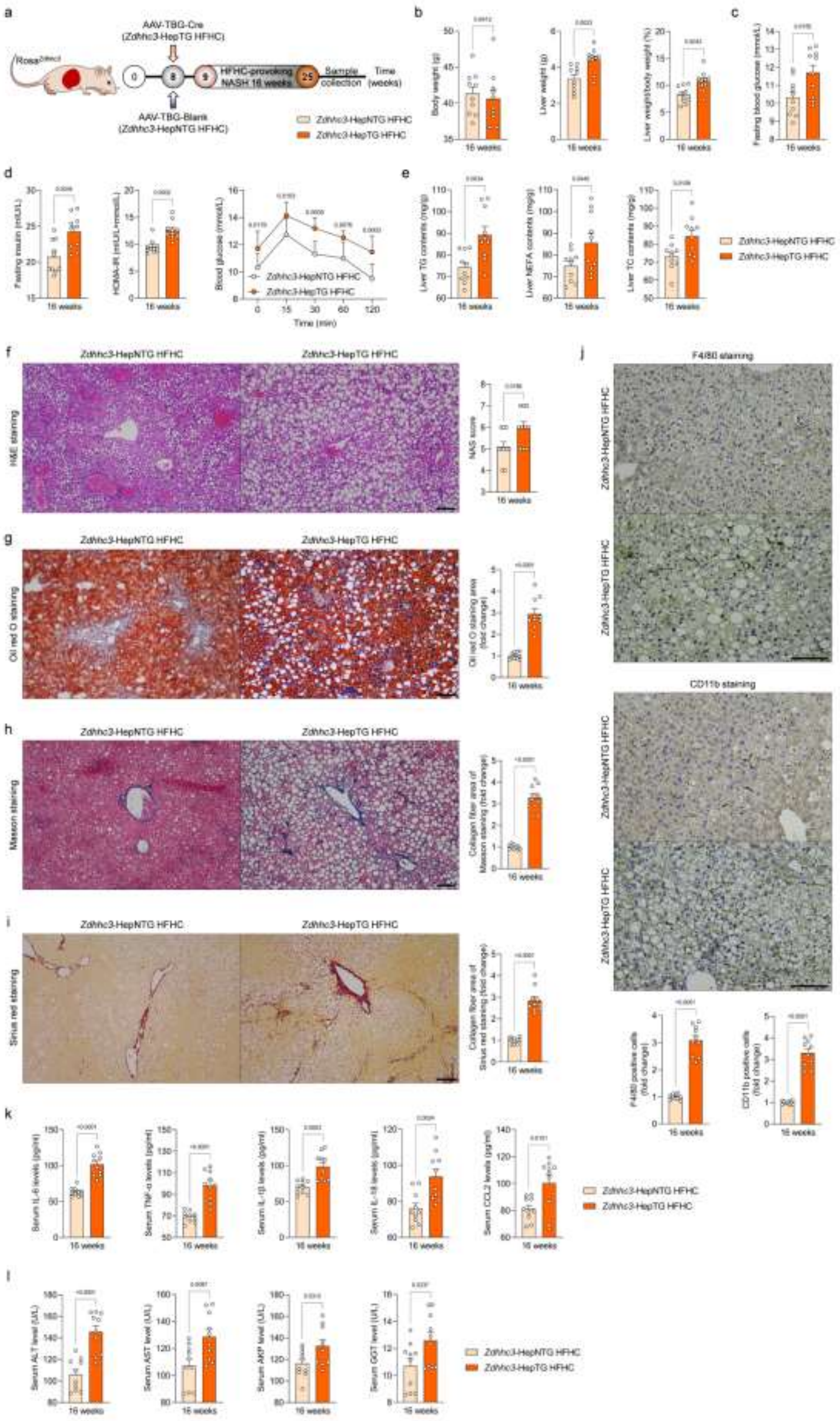

a
AAV-TBG-Cre
(Zdhhc3-HepTG HFHC)
AAV-TBG-Blank
(Zdhhc3-HepNTG HFHC)
HFHC-provoking NASH 16 weeks
Sample collection
Time (weeks)
Zdhhc3-HepNTG HFHC
Zdhhc3-HepTG HFHC
b
Body weight (g)
Liver weight (g)
Liver weight/body weight (%)
16 weeks
c
Fasting blood glucose (mmol/L)
d
Fasting insulin (mIU/L)
HOMA-IR (mIU/L×mmol/L)
Blood glucose (mmol/L)
Time (min)
e
Liver TG contents (mg/g)
Liver NEFA contents (mg/g)
Liver TC contents (mg/g)
f
H&E staining
NAS score
g
Oil red O staining
Oil red O staining area (fold change)
h
Masson staining
Collagen fiber area of Masson staining (fold change)
i
Sirius red staining
Collagen fiber area of Sirius red staining (fold change)
j
F4/80 staining
CD11b staining
F4/80 positive cells (fold change)
CD11b positive cells (fold change)
k
Serum IL-6 levels (pg/ml)
Serum TNF-α levels (pg/ml)
Serum IL-1β levels (pg/ml)
Serum IL-18 levels (pg/ml)
Serum CCL2 levels (pg/ml)
l
Serum ALT level (U/L)
Serum AST level (U/L)
Serum AKP level (U/L)
Serum GGT level (U/L)

Supplementary figure 10. Hepatocyte-specific *Zdhhc3* overexpression promotes HFHC-induced NASH pathologies.

a, Schematic plot of adeno-associated virus (serotype 8)-TBG-Cre (AAV-TBG-Cre)-mediated *Zdhhc3* overexpression in liver of HFHC-fed Rosa^{Zdhhc3} mice (*Zdhhc3*-HepTG HFHC). The AAV-TBG-Blank was used as control (*Zdhhc3*-HepNTG HFHC). **b-e**, Records for the body weight, liver weight and the ratio of liver weight/body weight (%) (**b**), fasting blood glucose levels (**c**), fasting insulin levels, HOMA-IR index and GTT test (**d**), and liver lipid contents including TG, NEFA and TC (**e**) in the *Zdhhc3*-HepTG HFHC and *Zdhhc3*-HepNTG HFHC group (*n*=10 mice per group; *P* < 0.05 vs. *Zdhhc3*-HepNTG HFHC group). **f-j**, Representative pictures of H&E staining and histological NAS score changes (**f**), oil red O staining (**g**), masson staining (**h**), sirius red staining (**i**), and F4/80 and CD11b positive cells expression (**j**) in indicated groups (magnification, 100× for H&E staining, oil red O staining, masson staining and sirius red staining; magnification, 200× for F4/80 and CD11b staining; *n*=10 images per group; *P* < 0.05 vs. *Zdhhc3*-HepNTG HFHC group). **k**, Records for inflammation-related cytokines profiles including IL-6, TNF-α, IL-1β, IL-18 and CCL2 in serum from *Zdhhc3*-HepTG HFHC and *Zdhhc3*-HepNTG HFHC mice (*n*=10 mice per group; *P* < 0.05 vs. *Zdhhc3*-HepNTG HFHC group). **l**, Records for liver function-related indicators including ALT, AST, AKP and GGT in serum from *Zdhhc3*-HepTG HFHC and *Zdhhc3*-HepNTG HFHC mice (*n*=10 mice per group; *P* < 0.05 vs. *Zdhhc3*-HepNTG HFHC group). Data are expressed as mean ± SEM. The relevant experiments presented in this part were performed independently at least three times. Significance determined by 2-sided Student's *t*-test. The *P* value less than 0.05 was considered as significant difference.

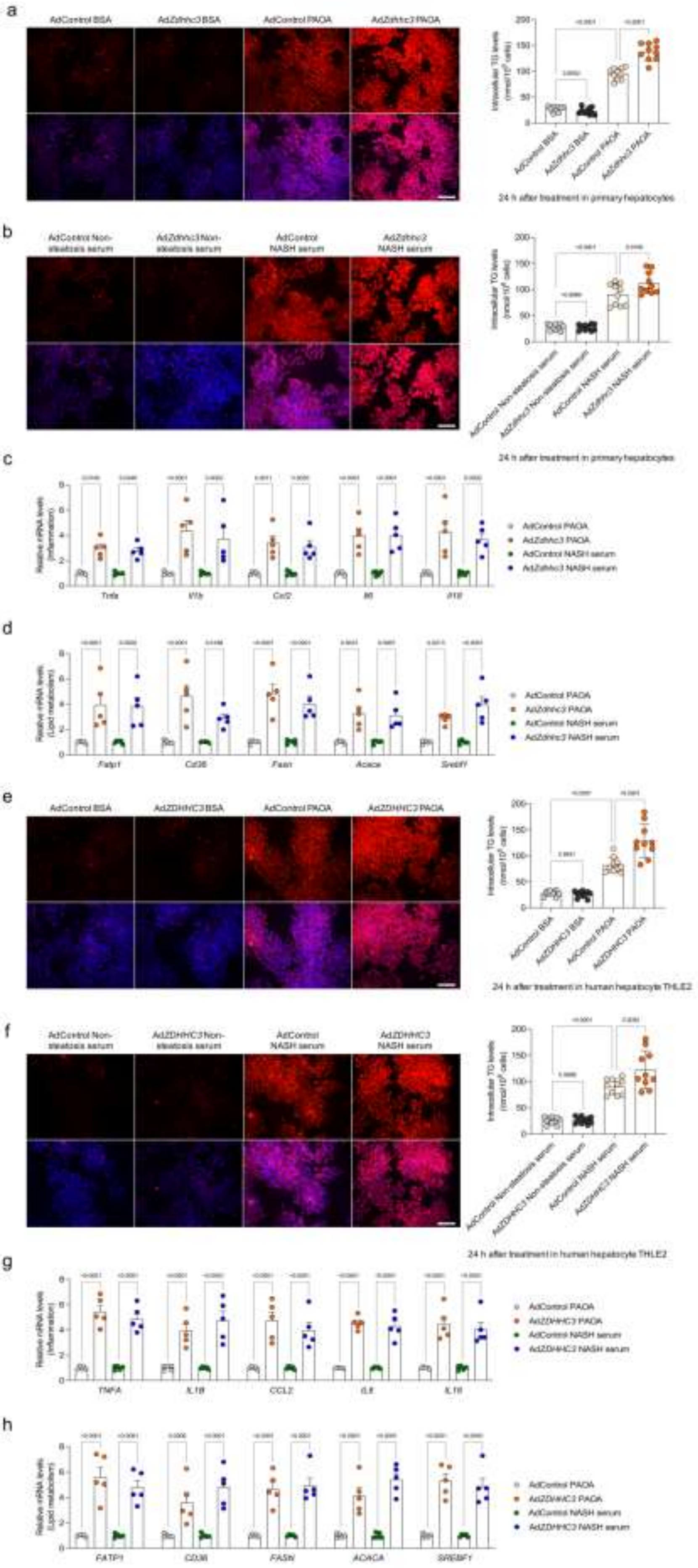
a
AdControl BSA
AdZdhhc3 BSA
AdControl PAOA
AdZdhhc3 PAOA
Intracellular TG levels
AdControl BSA
AdZdhhc3 BSA
AdControl PAOA
AdZdhhc3 PAOA
24 h after treatment in primary hepatocytes
b
AdControl Non-steatosis serum
AdZdhhc3 Non-steatosis serum
AdControl NASH serum
AdZdhhc3 NASH serum
Intracellular TG levels
24 h after treatment in primary hepatocytes
c
Relative mRNA levels (Inflammation)
Tnfa
Ccl2
AdControl PAOA
AdZdhhc3 PAOA
AdControl NASH serum
AdZdhhc3 NASH serum
d
Relative mRNA levels (Lipid metabolism)
Fatp1
Cd36
Fasn
Acaca
Srebf1
AdControl PAOA
AdZdhhc3 PAOA
AdControl NASH serum
AdZdhhc3 NASH serum
e
AdControl BSA
AdZDHHC3 BSA
AdControl PAOA
AdZDHHC3 PAOA
Intracellular TG levels
24 h after treatment in human hepatocyte THLE2
f
AdControl Non-steatosis serum
AdZDHHC3 Non-steatosis serum
AdControl NASH serum
AdZDHHC3 NASH serum
Intracellular TG levels
24 h after treatment in human hepatocyte THLE2
g
Relative mRNA levels (Inflammation)
TNFA
IL1B
CCL2
IL6
IL18
AdControl PAOA
AdZDHHC3 PAOA
AdControl NASH serum
AdZDHHC3 NASH serum
h
Relative mRNA levels (Lipid metabolism)
FATP1
CD36
FASN
ACACA
SREBF1
AdControl PAOA
AdZDHHC3 PAOA
AdControl NASH serum
AdZDHHC3 NASH serum

Supplementary figure 11. Increased ZDHHC3 accelerates lipid deposition, and inflammation *in vitro* upon challenge with metabolic insults.

a, Representative images and intracellular triglyceride (TG) analysis of the Nile red staining of primary hepatocytes that were transfected with Ad*Zdhhc3* and co-treated with PAOA for 24 hours (n=10 images per group; P < 0.05 vs. AdControl group). Scale bar, 200 μm. **b**, Representative images and intracellular triglyceride (TG) analysis of the Nile red staining of primary hepatocytes that were transfected with Ad*Zdhhc3* and co-treated with NASH serum for 24 hours (n=10 images per group; P < 0.05 vs. AdControl group). Scale bar, 200 μm. **c**, **d**, qPCR analysis showing the inflammation (**c**)- and lipid metabolism (**d**)-related key genes expression changes in indicated groups (n=5 per group; P < 0.05 vs. AdControl group). **e**, Representative images and intracellular triglyceride (TG) analysis of the Nile red staining of THLE2 cells that were transfected with Ad*ZDHHC3* and co-treated with PAOA for 24 hours (n=10 images per group; P < 0.05 vs. AdControl group). Scale bar, 200 μm. **f**, Representative images and intracellular triglyceride (TG) analysis of the Nile red staining of THLE2 cells that were transfected with Ad*ZDHHC3* and co-treated with NASH serum for 24 hours (n=10 images per group; P < 0.05 vs. AdControl group). Scale bar, 200 μm. **g**, **h**, qPCR analysis showing the inflammation (**g**)- and lipid metabolism (**h**)-related key genes expression changes in indicated groups (n=5 per group; P < 0.05 vs. AdControl group). Data are expressed as mean ± SEM. The relevant experiments presented in this part were performed independently at least three times. Significance determined by one-way analysis of variance (ANOVA) followed by Dunnett's multiple comparisons test analysis (**a**, **b**, **e**, **f**) or 2-sided Student's t-test (**c**, **d**, **g**, **h**). The P value less than 0.05 was considered as significant difference.

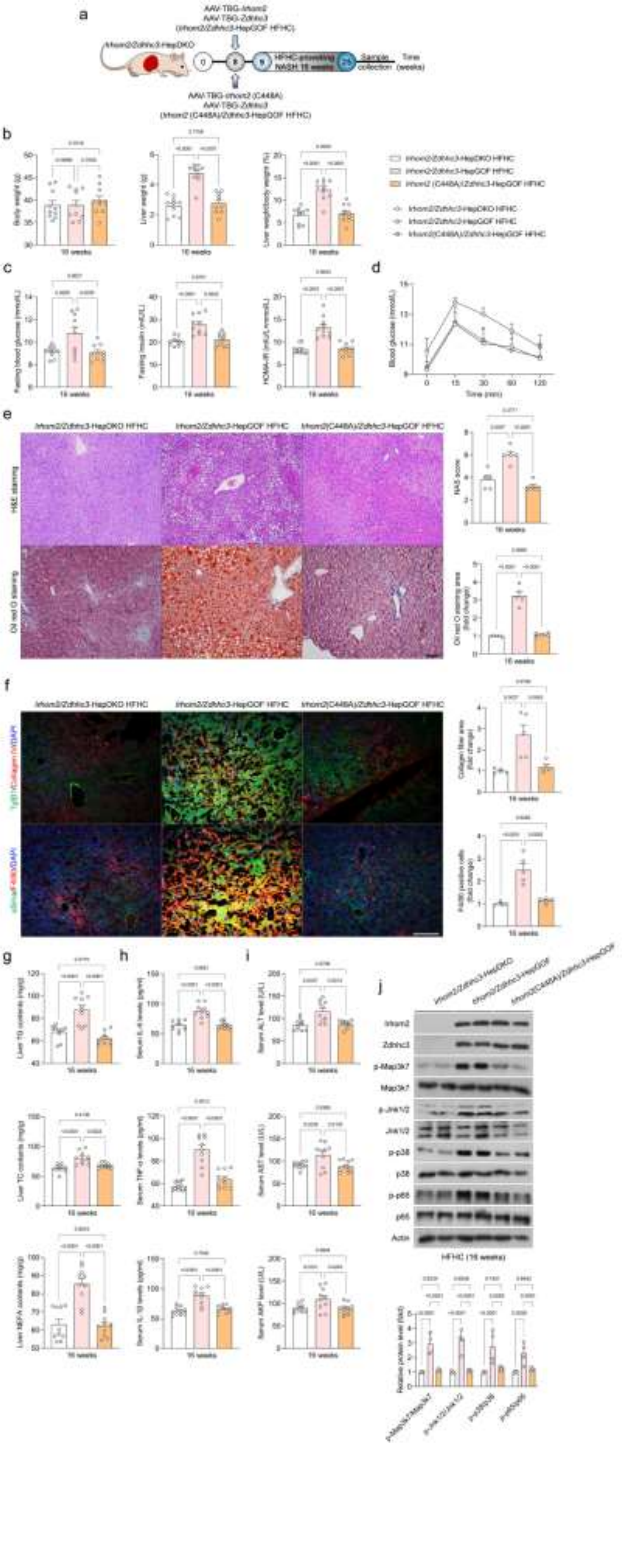

a
AAV-TBG-Irhom2
AAV-TBG-Zdhhc3
(Irhom2/Zdhhc3-HepGOF HFHC)
Irhom2/Zdhhc3-HepDKO
Sample collection
Time (weeks)
AAV-TBG-Irhom2 (C448A)
AAV-TBG-Zdhhc3
(Irhom2 (C448A)/Zdhhc3-HepGOF HFHC)
b
Body weight (g)
Liver weight (g)
Liver weight/body weight (%)
16 weeks
Irhom2/Zdhhc3-HepDKO HFHC
Irhom2/Zdhhc3-HepGOF HFHC
Irhom2 (C448A)/Zdhhc3-HepGOF HFHC
c
Fasting blood glucose (mmol/L)
Fasting insulin (mIU/L)
HOMA-IR
d
Blood glucose (mmol/L)
Time (min)
e
Irhom2/Zdhhc3-HepDKO HFHC
Irhom2/Zdhhc3-HepGOF HFHC
Irhom2(C448A)/Zdhhc3-HepGOF HFHC
H&E staining
Oil red O staining
NAS score
Oil red O staining area (fold change)
f
Collagen fiber area (fold change)
F4/80 positive cells (fold change)
g
Liver TG contents (mg/g)
Liver TC contents (mg/g)
Liver NEFA contents (mg/g)
h
Serum IL-6 levels (pg/ml)
Serum TNF-α levels (pg/ml)
Serum IL-1β levels (pg/ml)
i
Serum ALT level (U/L)
Serum AST level (U/L)
Serum AKP level (U/L)
j
Irhom2/Zdhhc3-HepDKO
Irhom2/Zdhhc3-HepGOF
Irhom2(C448A)/Zdhhc3-HepGOF
Irhom2
Zdhhc3
p-Map3k7
Map3k7
p-Jnk1/2
Jnk1/2
p-p38
p38
p-p65
p65
Actin
HFHC (16 weeks)
Relative protein level (fold)
p-Map3k7/Map3k7
p-Jnk1/2/Jnk1/2
p-p38/p38
p-p65/p65

Supplementary figure 12. Irhom2 with C448A mutant fails to exert acceleration in the setting of NASH pathogenesis.

a, Experimental design of AAV-mediated *Irhom2/Zdhhc3* or AAV-mediated *Irhom2* (C448A)*/Zdhhc3* restoration based on hepatocyte *Irhom2/Zdhhc3*-dual deficient mice, followed by feeding with HFHC diet for 16 weeks. **b-d**, Records for the body weight, liver weight and the ratio of liver weight/body weight (%) (LW/BW) (**b**), fasting blood glucose levels, fasting insulin levels, HOMA-IR index (**c**), glucose tolerance test (GTT) (**d**) of the HFHC-fed *Irhom2/Zdhhc3*-HepGOF mice and *Irhom2* (C448A)*/Zdhhc3*-HepGOF mice; HFHC-fed *Irhom2/Zdhhc3*-HepDKO mice were treated as corresponding control (*n*=10 mice per group; $P < 0.05$ vs. *Irhom2/Zdhhc3*-HepDKO HFHC group). **e**, **f**, Representative pictures of H&E staining, oil red O staining, histological NAS score (**e**) changes, Tgfβ1+collagen IV co-expression, and αSma+F4/80 co-expression (**f**) in indicated groups (magnification, 100× for H&E staining and oil red O staining; magnification, 200× for immunofluorescence staining; *n*=5 images per group; $P < 0.05$ vs. *Irhom2/Zdhhc3*-HepDKO HFHC group). **g-i**, Records for the liver TG, NEFA and TC contents (**g**), serum inflammation-related cytokines profiles including IL-6, TNF-α and IL-1β (**h**), and serum liver enzymes levels including ALT, AST and AKP (**i**) in the indicated groups (*n*=10 per parameter; $P < 0.05$ vs. *Irhom2/Zdhhc3*-HepDKO HFHC group). **j**, Representative western blotting bands showing the Irhom2, Zdhhc3, phosphorylated and total Map3k7, Jnk1/2, p38 and NF-κB p65 protein expression in the indicated groups (*n*=4 per parameter; $P < 0.05$ vs. *Irhom2/Zdhhc3*-HepDKO HFHC group). Data are expressed as mean ± SEM. The relevant experiments presented in this part were performed independently at least three times. Significance determined by one-way analysis of variance (ANOVA) followed by multiple comparisons test analysis. The *P* value less than 0.05 was considered as significant difference.

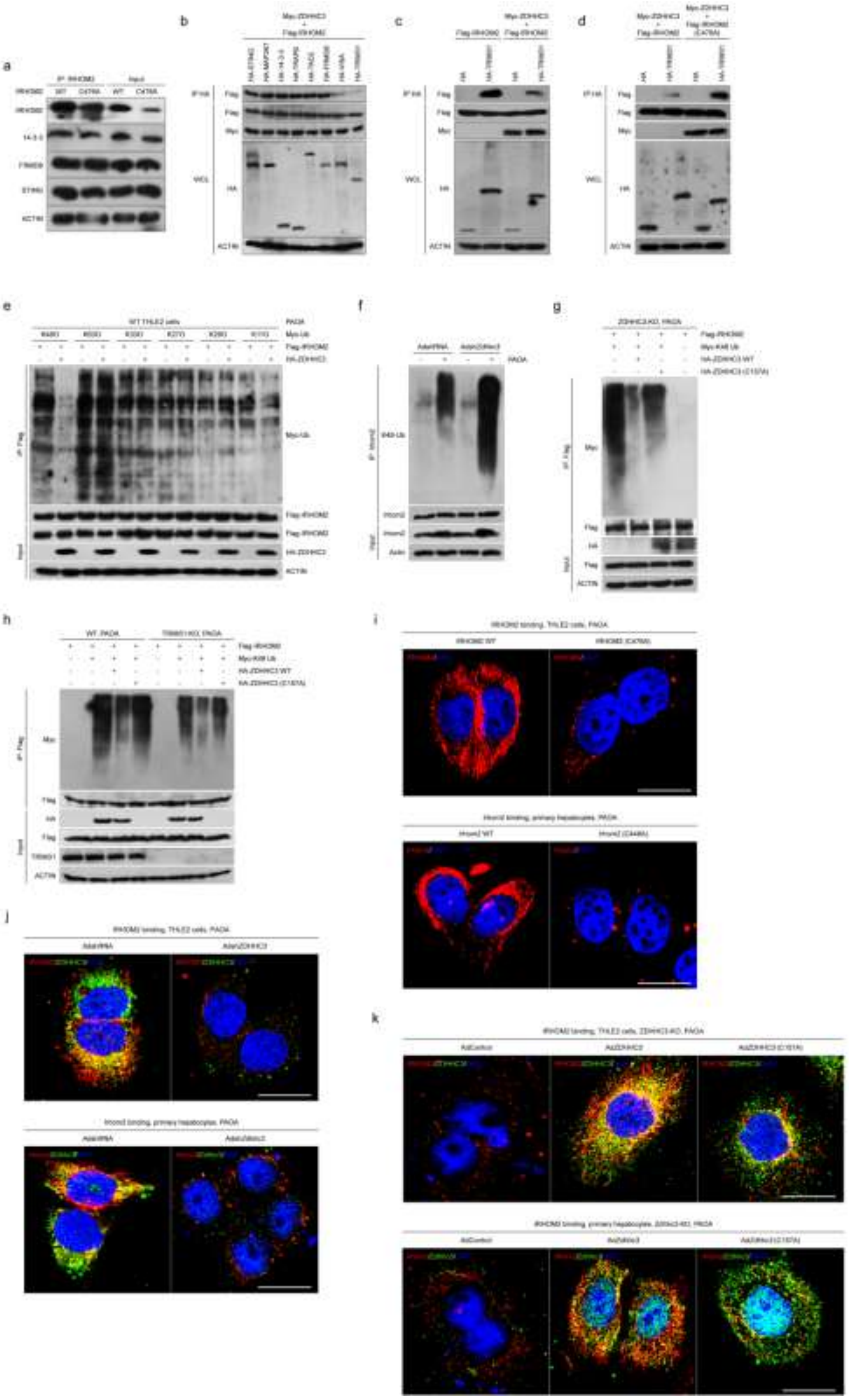

Supplementary figure 13. ZDHHC3-mediated palmitoylation blocks interaction between TRIM31 and IRHOM2.

a, IP detection indicating the interaction between IRHOM2 and other functional proteins associated with IRHOM2. Overexpressed IRHOM2 WT and IRHOM2 C476A mutant in THLE2 cells was immunoprecipitated with antibody for IRHOM2, followed by immunoblotting assay for the determination of interacting 14-3-3, FRMD8 and STING. **b**, IP analysis showing the effects of ZDHHC3 on binding between IRHOM2 and STING, FRMD8, 14-3-3, TRAPβ, TACE, VISA, MAP3K7 and TRIM31 in indicated vectors-transfected THLE2 cells, followed by immunoblotting

assay with Flag, HA, Myc and ACTIN antibodies. **c**, **d**, IP assay indicating the effects of ZDHHC3 on interaction between TRIM31 and IRHOM2 WT (**c**), and TRIM31 and IRHOM2 C476A mutant (**d**) in indicated plasmids-transfected THLE2 cells, followed by western blotting detection with Flag, HA, Myc and ACTIN antibodies. **e**, Ubiquitination levels of IRHOM2 after HA-ZDHHC3 overexpression and in response to PAOA challenge in THLE2 cells co-transfected with Flag-IRHOM2 and the indicated Myc-tagged ubiquitin vectors (K48O, K63O, K33O, K27O, K29O, K11O). Tail label K48 "O" means ubiquitin in which all lysines except K48 were mutated. **f**, K48-linked ubiquitin (K48-Ub) and corresponding Irhom2 expression in mouse primary hepatocytes transfected with Adsh*Zdhhc3* in the presence or absence of PAOA challenge. **g**, Representative immunoprecipitation and western blotting bands exhibiting the indicated protein in the *ZDHHC3*-knockout THLE2 cells transfected with Flag-IRHOM2, HA-ZDHHC3 WT, HA-ZDHHC3 C157A and Myc-tagged K48 ubiquitin constructs under PAOA administration. **h**, The levels of ubiquitinated and Flag-IRHOM2 after IP of Flag and immunoblotting of Flag-IRHOM2 in input lysates of WT THLE2 cells and *TRIM31*-deficient THLE2 cells transfected with Flag-IRHOM2, HA-ZDHHC3 WT, HA-ZDHHC3 C157A and Myc-tagged K48 ubiquitin vectors in response to PAOA challenge. **i**, Representative immunofluorescence images showing the effects of PAOA on IRHOM2 binding to cell surface. Upper, THLE2 cells were overexpressed with IRHOM2 WT or IRHOM2 C476A mutant. Lower, mouse primary hepatocytes were similarly transfected with Irhom2 WT or Irhom2 C448A mutant. The fixed sections treated with human IRHOM2 or mouse Irhom2 recombinant protein were subjected to immunofluorescence assay of IRHOM2 (red) and DAPI (blue) (*n*=10 images per group). Scale bar, 10 μm. **j**, Representative immunofluorescence images showing the effects of ZDHHC3 on IRHOM2 binding to cell surface in response to PAOA challenge. Upper, THLE2 cells were transfected with Adsh*ZDHHC3* or AdshRNA control. Lower, mouse primary hepatocytes were similarly transfected with Adsh*Zdhhc3* or AdshRNA control. The fixed sections treated with human IRHOM2 or mouse Irhom2 recombinant protein, and ZDHHC3 antibody were subjected to immunofluorescence assay of IRHOM2 (red), ZDHHC3 (green) and DAPI (blue) (*n*=10 images per group). Scale bar, 10 μm. **k**, Representative immunofluorescence images showing the effects of ZDHHC3 or ZDHHC3 mutant on IRHOM2 binding to cell surface upon PAOA administration. Upper, *ZDHHC3*-deleted THLE2 cells were transfected with Ad*ZDHHC3* or Ad*ZDHHC3* C157A to restore ZDHHC3 expression. Lower, mouse primary hepatocytes with *Zdhhc3* ablation were similarly transfected with Ad*Zdhhc3* or Ad*Zdhhc3* C157A. The fixed sections treated with human IRHOM2 or mouse Irhom2 recombinant protein, and ZDHHC3 antibody were subjected to immunofluorescence assay of IRHOM2 (red), ZDHHC3 (green) and DAPI (blue) (*n*=10 images per group). Scale bar, 10 μm. The relevant experiments presented in this part were performed independently at least three times.

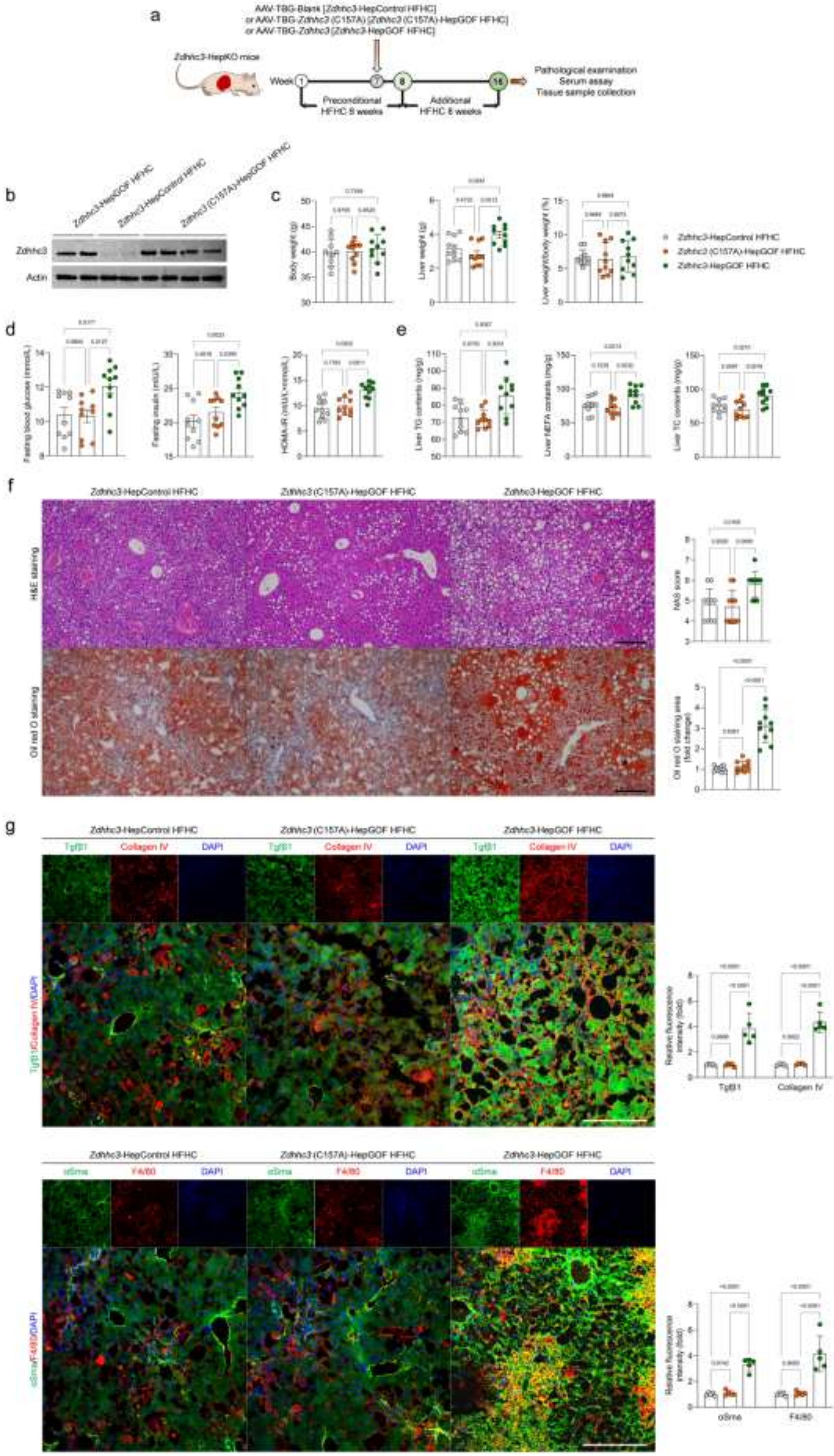
a
AAV-TBG-Blank [Zdhhc3-HepControl HFHC]
or AAV-TBG-Zdhhc3 (C157A) [Zdhhc3 (C157A)-HepGOF HFHC]
or AAV-TBG-Zdhhc3 [Zdhhc3-HepGOF HFHC]
Zdhhc3-HepKO mice
Week
Preconditional HFHC 8 weeks
Additional HFHC 8 weeks
Pathological examination
Serum assay
Tissue sample collection
b
Zdhhc3-HepGOF HFHC
Zdhhc3-HepControl HFHC
Zdhhc3 (C157A)-HepGOF HFHC
Zdhhc3
Actin
c
Body weight (g)
Liver weight (g)
Liver weight/body weight (%)
Zdhhc3-HepControl HFHC
Zdhhc3 (C157A)-HepGOF HFHC
Zdhhc3-HepGOF HFHC
d
Fasting blood glucose (mmol/L)
Fasting insulin (mIU/L)
HOMA-IR
e
Liver TG contents (mg/g)
Liver NEFA contents (mg/g)
Liver TC contents (mg/g)
f
Zdhhc3-HepControl HFHC
Zdhhc3 (C157A)-HepGOF HFHC
Zdhhc3-HepGOF HFHC
H&E staining
Oil red O staining
NAS score
Oil red O staining area (fold change)
g
Zdhhc3-HepControl HFHC
Zdhhc3 (C157A)-HepGOF HFHC
Zdhhc3-HepGOF HFHC
Tgfβ1
Collagen IV
DAPI
Tgfβ1/Collagen IV/DAPI
Relative fluorescence intensity (fold)
Tgfβ1
Collagen IV
Zdhhc3-HepControl HFHC
Zdhhc3 (C157A)-HepGOF HFHC
Zdhhc3-HepGOF HFHC
αSma
F4/80
DAPI
αSma/F4/80/DAPI
Relative fluorescence intensity (fold)
αSma
F4/80

Supplementary figure 14. *Zdhhc3* with C157A mutant restoration of DHHC domain fails to facilitate NASH diet-triggered progression of steatohepatitis.

a, Schematic diagram of adeno-associated virus (serotype 8)-TBG-Cre (AAV-TBG-Cre)-mediated *Zdhhc3* restoration (*Zdhhc3*-HepGOF HFHC) and *Zdhhc3* (C157A) restoration [*Zdhhc3* (C157A)-HepGOF HFHC] in liver of HFHC-fed *Zdhhc3*-HepKO mice. The AAV-TBG-Blank was used as control (*Zdhhc3*-HepControl HFHC). **b**, Characteristic of Zdhhc3 expression in indicated groups (*n*=4 mice per group). **c-e**, Records for the body weight, liver weight and the ratio of liver weight/body weight (%) (**c**), fasting blood glucose levels, fasting insulin levels and HOMA-IR index (**d**), and liver TG, NEFA and TC contents (**e**) in the indicated mice (*n*=10 mice per group; $P < 0.05$ vs. *Zdhhc3*-HepControl HFHC). **f**, Representative pictures of H&E staining, corresponding NAS score and oil red O staining showing the hepatosteatosis, inflammatory infiltration and liver injury levels in the indicated mice (*n*=10 mice per group; $P < 0.05$ vs. *Zdhhc3*-HepControl HFHC). **g**, Representative pictures of immunofluorescence analysis of Tgfβ & Collagen IV, and αSma & F4/80 coexpression, respectively (*n*=10 images per group; $P < 0.05$ vs. *Zdhhc3*-HepControl HFHC). Scale bars, 100 μm. Data are expressed as mean ± SEM. The relevant experiments presented in this part were performed independently at least three times. Significance determined by one-way analysis of variance (ANOVA) followed by Dunnett’s multiple comparisons test analysis. The *P* value less than 0.05 was considered as significant difference.

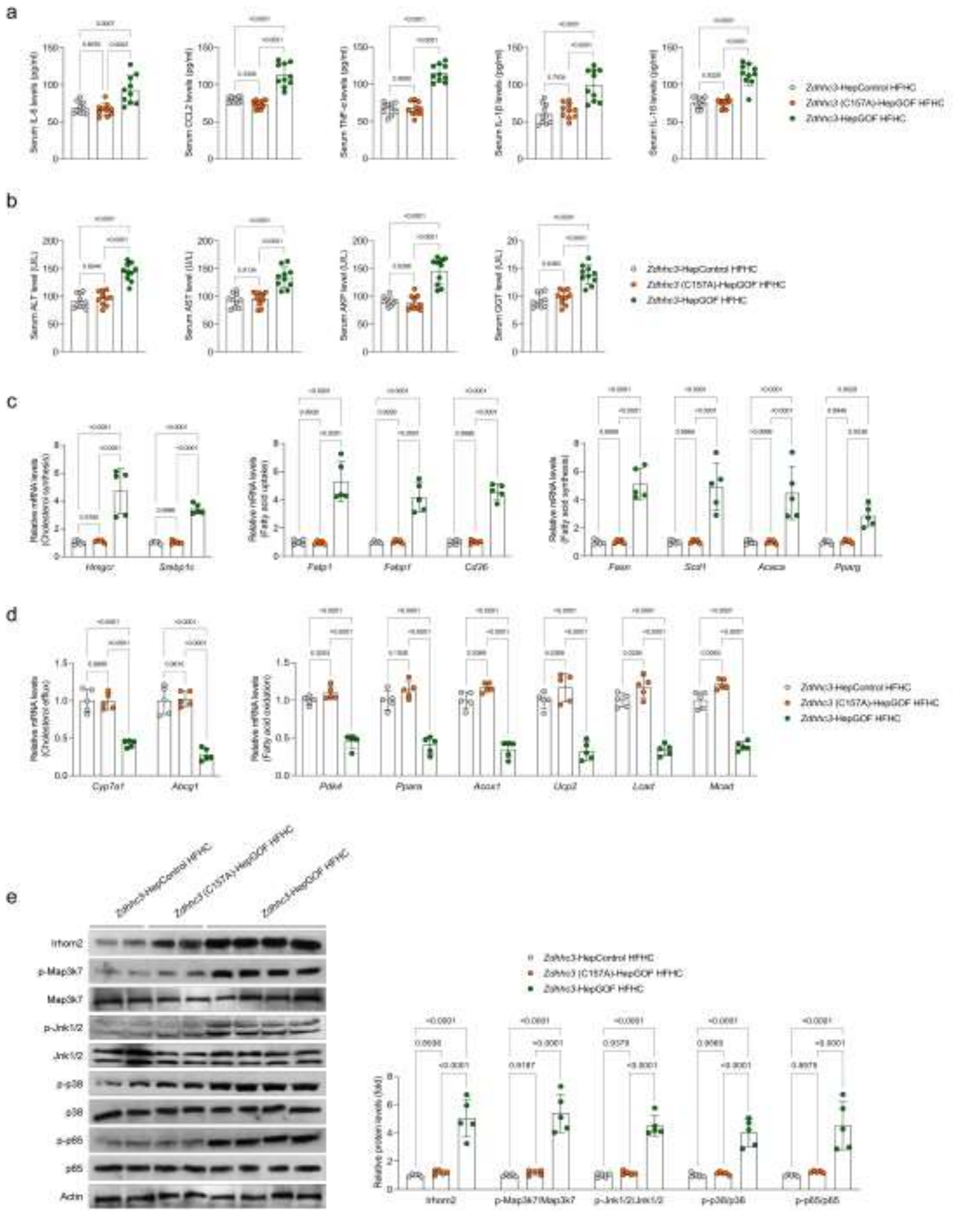

Supplementary figure 15. *Zdhhc3* with C157A mutant restoration in DHHC domain did not facilitate NASH diet-triggered progression of steatohepatitis by activating Irhom2-Map3k7 signaling axis.

a, **b**, Records for serum pro-inflammatory cytokines IL-6, TNF-α, IL-18, CCL2 and IL-1β contents (**a**) and liver function indicators including serum ALT, AST, AKP and GGT contents (**b**) in indicated groups (*n*=10 mice per group; *P* < 0.05 vs. *Zdhhc3*-HepControl HFHC). **c**, Representative mRNA levels of cholesterol synthesis (*Hmgcr* & *Srebp1c*)-, fatty acid uptake (*Fatp1*, *Fabp1* & *Cd36*)- and fatty acid synthesis (*Fasn*, *Scd1*, *Acaca* & *Pparg*)-associated genes expression in livers from indicated groups (*n*=5 mice per group; *P* < 0.05 vs. *Zdhhc3*-HepControl HFHC). **d**, Representative mRNA levels of cholesterol efflux (*Cyp7a1* & *Abcg1*)-, and fatty acid oxidation (*Pdk4*, *Ppara*, *Acox1*, *Ucp2*, *Lcad* & *Mcad*)-associated genes expression in livers from indicated groups (*n*=5 mice per group; *P* < 0.05 vs. *Zdhhc3*-HepControl HFHC). **e**, Representative

western blotting bands showing Irhom2-Map3k7 signaling axis including Irhom2, p-Map3k7, p-Jnk1/2, p-p38 and p-p65 activation in liver samples from the indicated groups (*n*=5 mice per group; $P < 0.05$ vs. *Zdhhc3*-HepControl HFHC). Data are expressed as mean ± SEM. The relevant experiments presented in this part were performed independently at least three times. Significance determined by one-way analysis of variance (ANOVA) followed by Dunnett's multiple comparisons test analysis. The *P* value less than 0.05 was considered as significant difference.

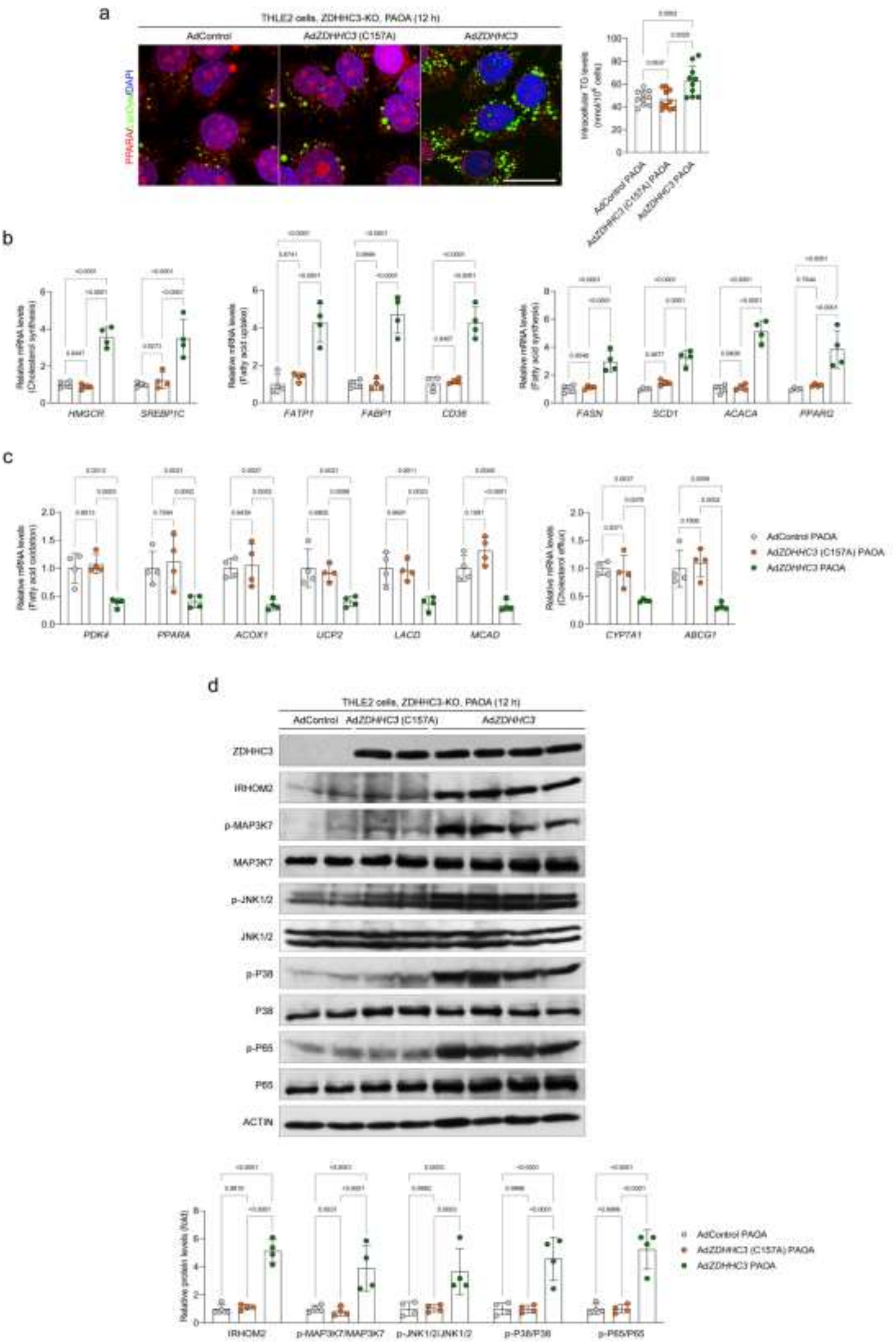

Supplementary figure 16. *Zdhhc3* with C157A mutant restoration in DHHC domain cannot promote activation of Irhom2-Map3k7 signaling axis over the course of PAOA challenge.

a, Representative immunofluorescence images showing the effects of ZDHHC3 or ZDHHC3 mutant on lipid deposition upon PAOA administration. *ZDHHC3*-deleted THLE2 cells were transfected with Ad*ZDHHC3* or Ad*ZDHHC3* C157A to restore ZDHHC3 expression. The fixed sections treated with PPARA antibody and LipiDye were subjected to immunofluorescence assay

of PPARA (red), lipid droplets formation (green) and DAPI (blue) (*n*=10 images per group). Scale bar, 10 μm. The right graph showing the intracellular TG contents in indicated groups (*n*=10 samples per group; *P* < 0.05 vs. AdControl PAOA). **b**, Representative mRNA levels of cholesterol synthesis (*HMGCR* & *SREBP1C*)-, fatty acid uptake (*FATP1*, *FABP1* & *CD36*)- and fatty acid synthesis (*FASN*, *SCD1*, *ACACA* & *PPARG*)-associated genes expression in indicated groups (*n*=4 per group; *P* < 0.05 vs. AdControl PAOA). **c**, Representative mRNA levels of fatty acid oxidation (*PDK4*, *PPARA*, *ACOX1*, *UCP2*, *LCAD* & *MCAD*)-, and cholesterol efflux (*CYP7A1* & *ABCG1*)-associated genes expression in indicated groups (*n*=4 mice per group; *P* < 0.05 vs. AdControl PAOA). **d**, Representative western blotting bands showing ZDHHC3 and IRHOM2-MAP3K7 signaling axis including IRHOM2, p-MAP3K7, p-JNK1/2, p-P38 and p-P65 activation in the indicated groups (*n*=4 per group; *P* < 0.05 vs. AdControl PAOA). Data are expressed as mean ± SEM. The relevant experiments presented in this part were performed independently at least three times. Significance determined by one-way analysis of variance (ANOVA) followed by Dunnett's multiple comparisons test analysis. The *P* value less than 0.05 was considered as significant difference.

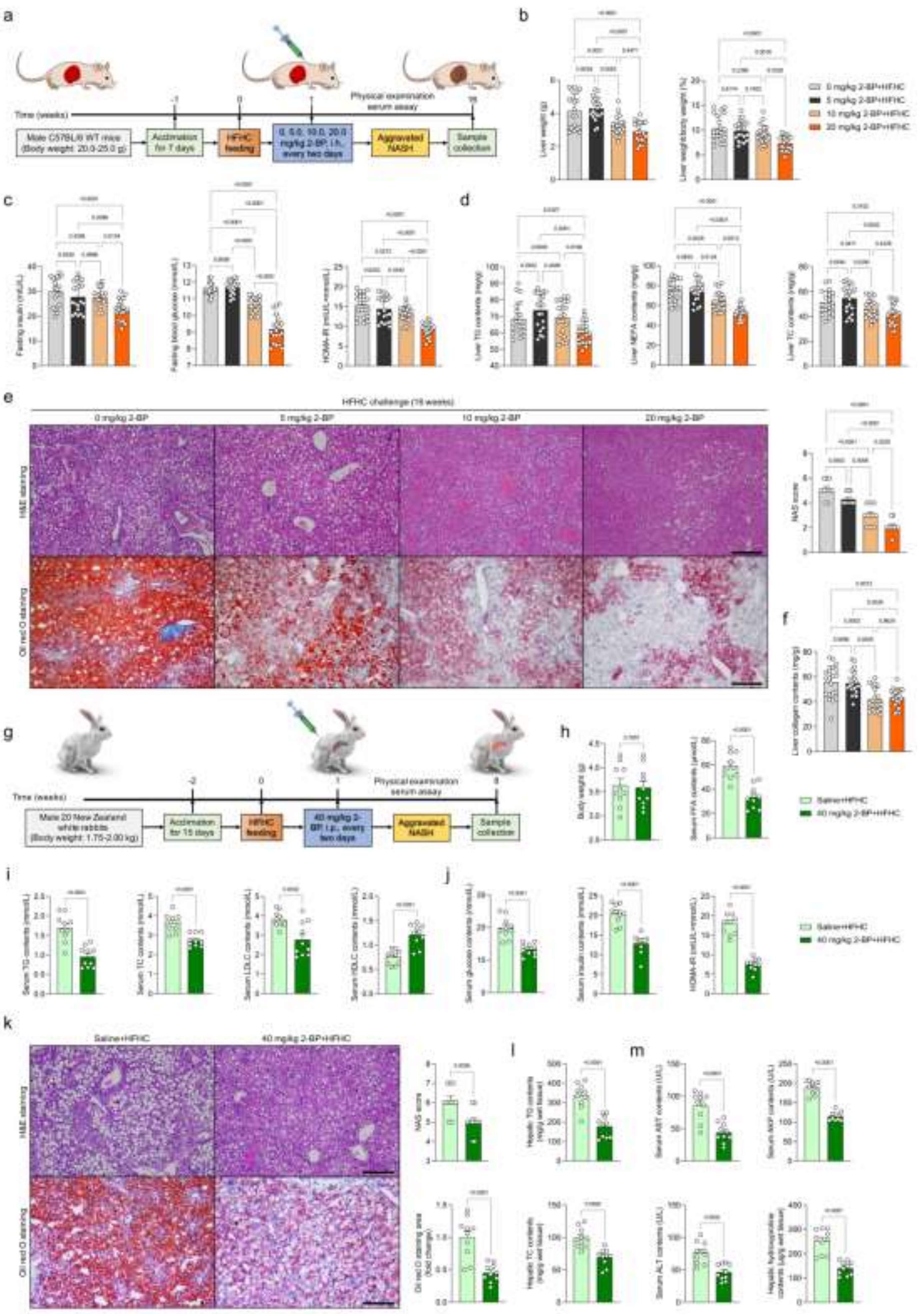

Supplementary figure 17. Pharmacologic effects of palmitoylation inhibitor 2-bromopalmitate (2-BP) on NASH in rodent and rabbit model.

a, Experimental design outline of male WT mice with 0-20 mg/kg 2-BP administration, followed by 16-weeks HFHC diet-induced NASH model. **b**, **c**, Records for the liver weight and liver weight/body weight ratio (%) (**b**), and fasting insulin, fasting blood glucose and corresponding HOMA-IR index (**c**) in indicated groups (*n*=20 mice per group; *P* < 0.05 vs. 0 mg/kg

2-BP+HFHC). **d**, Records for liver TG, NEFA and TC contents in indicated groups (*n*=20 mice per group; *P* < 0.05 vs. 0 mg/kg 2-BP+HFHC). **e**, **f**, Representative images of H&E staining, Oil red O staining, and corresponding NAS score (**e**) in indicated mice (magnification, 100×; *n*=10 images per group; *P* < 0.05 vs. 0 mg/kg 2-BP+HFHC). The hepatic collagen contents (**f**) were recorded for the indicated mice (*n*=20 per group; *P* < 0.05 vs. 0 mg/kg 2-BP+HFHC). **g**, Experimental design outline of male New Zealand white rabbits with 40 mg/kg 2-BP administration, followed by 8-weeks HFHC diet-induced NASH model (40 mg/kg 2-BP+HFHC). The rabbits treated with saline were used as control (Saline+HFHC). (**h**-**j**) Records for the body weight, serum FFA contents (**h**), and serum TG contents, TC contents, LDLC contents, HDLC levels (**i**), serum glucose levels, insulin levels and HOMA-IR levels (**j**) in Saline+HFHC and 40 mg/kg 2-BP+HFHC groups (*n*=10 rabbits per group; *P* < 0.05 vs. Saline+HFHC). **k**, **l**, Representative images of H&E staining and Oil red O staining (**k**), and corresponding NAS score, and hepatic TG & TC contents (**l**) in indicated groups (magnification, 100×; *n*=10 images per group; *P* < 0.05 vs. Saline+HFHC). **m**, Records for serum AST, ALT and AKP contents, and liver hydroxyproline contents in Saline+HFHC and 40 mg/kg 2-BP+HFHC groups (*n*=10 rabbits per group; *P* < 0.05 vs. Saline+HFHC). Data are expressed as mean ± SEM. The relevant experiments presented in this part were performed independently at least three times. Significance determined by one-way analysis of variance (ANOVA) followed by Dunnett's multiple comparisons test analysis (**b-f**) or 2-sided Student's *t*-test (**h-m**). The *P* value less than 0.05 was considered as significant difference.

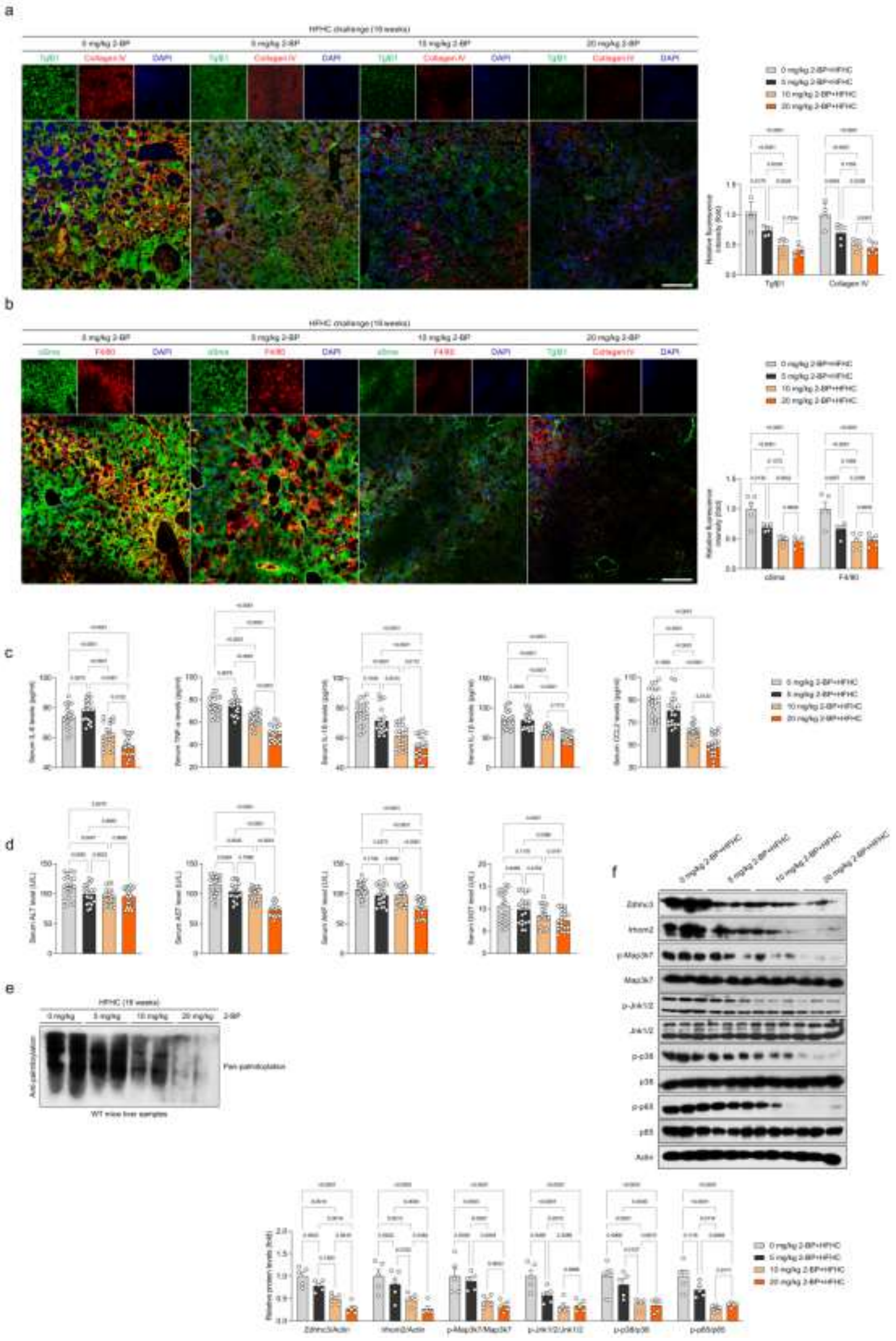

Supplementary figure 18. Treatment with 2-BP significantly protects against steatohepatitis by suppressing Irhom2-Map3k7 signaling axis in response to HFHC diet administration.

a, **b**, Representative pictures of immunofluorescence analysis of Tgfβ & Collagen IV (**a**), and αSma & F4/80 (**b**) coexpression, respectively (n=5 images per group; P < 0.05 vs. 0 mg/kg 2-BP+HFHC). Scale bars, 100 μm. **c**, **d**, Records for serum pro-inflammatory cytokines IL-6,

TNF-α, IL-18, CCL2 and IL-1β contents (**c**) and liver function indicators including serum ALT, AST, AKP and GGT contents (**d**) in indicated groups (*n*=20 samples per group; $P < 0.05$ vs. 0 mg/kg 2-BP+HFHC). **e**, Representative western blotting analysis showing the pan-palmitoylation levels in liver samples collected from the indicated mice groups (*n*=4 images per group). **f**, Representative western blotting bands showing Zdhhc3 and Irhom2-Map3k7 signaling axis including Irhom2, p-Map3k7, p-Jnk1/2, p-p38 and p-p65 activation in the indicated groups (*n*=5 per group; $P < 0.05$ vs. 0 mg/kg 2-BP+HFHC). Data are expressed as mean ± SEM. The relevant experiments presented in this part were performed independently at least three times. Significance determined by one-way analysis of variance (ANOVA) followed by Dunnett's multiple comparisons test analysis. The *P* value less than 0.05 was considered as significant difference.
